# Nuclear pore heterogeneity influences HIV-1 infection and the antiviral activity of MX2

**DOI:** 10.7554/eLife.35738

**Published:** 2018-08-07

**Authors:** Melissa Kane, Stephanie V Rebensburg, Matthew A Takata, Trinity M Zang, Masahiro Yamashita, Mamuka Kvaratskhelia, Paul D Bieniasz

**Affiliations:** 1 Laboratory of RetrovirologyThe Rockefeller UniversityNew YorkUnited States; 2 Division of Infectious DiseasesUniversity of Colorado School of MedicineAuroraUnited States; 3 Howard Hughes Medical InstituteNew YorkUnited States; 4 Aaron Diamond AIDS Research CenterNew YorkUnited States; Icahn School of Medicine at Mount SinaiUnited States; National Institute of Biological SciencesChina

**Keywords:** HIV, nuclear import, integration, nucleoporin, Virus

## Abstract

HIV-1 accesses the nuclear DNA of interphase cells via a poorly defined process involving functional interactions between the capsid protein (CA) and nucleoporins (Nups). Here, we show that HIV-1 CA can bind multiple Nups, and that both natural and manipulated variation in Nup levels impacts HIV-1 infection in a manner that is strikingly dependent on cell-type, cell-cycle, and cyclophilin A (CypA). We also show that Nups mediate the function of the antiviral protein MX2, and that MX2 can variably inhibit non-viral NLS function. Remarkably, both enhancing and inhibiting effects of cyclophilin A and MX2 on various HIV-1 CA mutants could be induced or abolished by manipulating levels of the Nup93 subcomplex, the Nup62 subcomplex, NUP88, NUP214, RANBP2, or NUP153. Our findings suggest that several Nup-dependent ‘pathways’ are variably exploited by HIV-1 to target host DNA in a cell-type, cell-cycle, CypA and CA-sequence dependent manner, and are differentially inhibited by MX2.

## Introduction

Access to the chromosomal DNA contained within the nucleus of target cells is critical for retroviral integration and replication. Among retroviruses, the lentiviruses are uniquely efficient in their ability to enter the nucleus of interphase cells, in which the nuclear membrane is intact. The viral capsid (CA) is the key viral determinant of the ability of HIV-1 to infect non-dividing cells (Yamashita and Emerman, 2004), and CA mutants can specifically disrupt the ability of HIV-1 to infect non-dividing cells (Yamashita et al., 2007). In addition to CA itself, cellular CA-interacting proteins have been suggested to regulate or otherwise influence HIV-1 nuclear import and infection, including the peptidylprolyl isomerase cyclophilin A (CypA) and the mRNA processing protein cleavage and polyadenylation specificity factor 6 (CPSF6) (De Iaco et al., 2013; Lee et al., 2010; Price et al., 2012). CA can also directly interact with two nucleoporins (Nups) NUP153 and RANBP2(NUP358), that appear to play a major role in HIV-1 nuclear import (Bichel et al., 2013; Di Nunzio et al., 2013; Lin et al., 2013; Matreyek et al., 2013; Schaller et al., 2011; Zhang et al., 2010), A number of other Nups or nuclear transport receptors have also been implicated as co-factors for HIV-1 infection in genome-wide RNA interference screens (Brass et al., 2008; König et al., 2008; Yeung et al., 2009; Zhou et al., 2008). CA-dependent interactions with Nups also appear to play a role in determining where HIV-1 proviral DNA is integrated into the host genome (Di Nunzio et al., 2013; Koh et al., 2013; Ocwieja et al., 2011; Schaller et al., 2011), perhaps by directing HIV-1 preintegration complexes to specific nuclear import pathways or through effects on chromatin architecture (Lelek et al., 2015; Marini et al., 2015; Wong et al., 2015).

Approximately, 30 distinct Nups are present in the nuclear pore complex (NPC) that is organized in repetitively arranged subcomplexes with 8-fold rotational symmetry. The NPC has a tripartite architecture, with a central major architectural scaffold, that includes the transmembrane Nups, the Nup107, Nup93, and Nup62 subcomplexes. The NPC is appended with cytoplasmic filaments, and the nuclear basket (an assembly of 8 intranuclear filaments joined into a basket) (Fernandez-Martinez and Rout, 2012; Hoelz et al., 2011). Although NPCs are generally depicted as static structures, some components are dynamic. Specifically, the scaffold Nups remain associated with the NPC for hours to days while other components, including the phenylalanine-glycine (FG) repeat Nups, are associated with the NPC only for minutes at a time (Morchoisne-Bolhy et al., 2015; Rabut et al., 2004a). Recent structural studies suggest the existence of conformational flexibility in the main architectural elements of the NPC (the Nup107 or Y complex, the Nup62 complex, and the Nup93 complex or linker Nups) (reviewed in (Knockenhauer and Schwartz, 2016)). Moreover, a number of reports have also pointed to compositional variability of the NPC both within and between cells (D'Angelo et al., 2009; Gomez-Cavazos and Hetzer, 2015; Lowe et al., 2015; Lupu et al., 2008; Ori et al., 2013), and altered expression under certain conditions, such as upon IFN stimulation (Enninga et al., 2002). These reports raise the intriguing possibility that NPCs with different compositions may be poised for the transport of different cargos. Transport of substrates through the NPC is regulated by members of a family of nuclear transport receptors (NTRs) known as karyopherins (and/or importins/transportins). NTRs bind cargoes via the recognition of a nuclear localization signal (NLS) or nuclear export signal (NES) to form a transport complex, which is generally regulated by the GTPase, RAN. The distinction between a ‘stationary’ Nup and a ‘mobile’ NTR has been recently blurred by findings of structural and functional similarities between some Nups and NTRs (Andersen et al., 2013), and evidence that importin-β (KPNB1) can stably associate with the NPC and modulate nuclear pore permeability. Notably, diversity in NLS/NES sequences, intra/intermolecular masking of NLS/NES, variation in NTR expression and NPC composition (Chatel and Fahrenkrog, 2012; Sorokin et al., 2007; Terry et al., 2007) all have the potential to influence the rate of nuclear transport.

MX2, an antiviral protein whose expression is strongly upregulated by type 1 interferon, localizes to the NPC (Goujon et al., 2013; Kane et al., 2013; King et al., 2004; Liu et al., 2013). Like the better-studied MX1 protein, MX2 is comprised of a GTPase domain connected to a carboxy-terminal stalk domain via a tripartite bundle-signaling element (BSE) (Haller et al., 2015). Unlike MX1, MX2 contains an NLS-like sequence in its first 25 amino acids that is required for NPC localization (Kane et al., 2013; King et al., 2004; Melén et al., 1996). MX1 can inhibit a number of viruses, but not retroviruses (Haller et al., 2015), while MX2 was recently shown to inhibit infection by HIV-1 and other primate lentiviruses. (Goujon et al., 2013; Kane et al., 2013; Liu et al., 2013). The antiretroviral activity and specificity of MX2 is governed by the N-terminal domain that is absent in MX1 (Busnadiego et al., 2014; Goujon et al., 2014). Moreover, GTPase function and higher order oligomerization are generally required for the antiviral activity of MX1 (Haller et al., 2015) but are dispensable for the antiviral activity of MX2 (Dicks et al., 2016; Fribourgh et al., 2014; Goujon et al., 2014, 2013; Kane et al., 2013; Matreyek et al., 2014). Thus, the targets and mechanisms underlying inhibition of viral infection by MX1 and MX2 are quite distinct.

MX2 inhibits HIV-1 infection after the completion of reverse transcription but prior to the chromosomal integration of viral DNA (Goujon et al., 2013; Kane et al., 2013; Matreyek et al., 2014). For this reason, and because MX2 inhibits HIV-1 infection more potently in non-dividing cells (Kane et al., 2013; Yamashita and Emerman, 2009), current models suggest that MX2 acts by preventing nuclear import of the viral preintegration complex. Additionally, the viral capsid is the major viral determinant of MX2 sensitivity, and several single amino-acid substitutions in CA have been identified that confer partial or complete resistance to MX2 (Busnadiego et al., 2014; Goujon et al., 2013; Kane et al., 2013; Liu et al., 2013, 2015). MX2 has also been found to directly bind the HIV-1 CA, but the relevance of this binding for viral inhibition is unclear, as MX2-resistant CA proteins are also bound by MX2 (Fribourgh et al., 2014; Fricke et al., 2014). Notably, many of the CA mutations conferring partial or complete MX2 resistance, such as G89V, N57A/S, N74D, and A92E, are also known to affect binding to and/or requirements for cellular proteins implicated in HIV-1 nuclear import including CypA, CPSF6, NUP153, and RANBP2 (Hatziioannou et al., 2005; Lee et al., 2010; Matreyek et al., 2013; Rihn et al., 2013; Schaller et al., 2011; Sokolskaja et al., 2004). These mutants alter infection in non-dividing cells, and/or altere integration site selection, suggesting the possibility that they may utilize distinct pathways to access nuclear DNA.

MX2 antiviral activity is also affected by CypA, a ubiquitous and abundantly expressed member of a family of peptidyl-prolyl isomerases, whose role in HIV-1 infection is enigmatic (Luban et al., 1993). Disruption of the interaction between HIV-1 and CypA, either genetically or by cyclosporine A (CsA) addition, reduces the efficiency of HIV-1 infection (reviewed in (Campbell and Hope, 2015)), and some studies have indicated that CypA-CA interactions influence nuclear entry (De Iaco and Luban, 2014; Schaller et al., 2011). However, defining a precise role for CypA in HIV-1 infection has proved difficult because CsA treatment has diverse enhancing and inhibiting effects on infection that are determined by CA mutations and target cell type (reviewed in (Campbell and Hope, 2015; Yamashita and Engelman, 2017)).

Here, we explore the functional interactions between HIV-1 CA, Nups, MX2, and CypA. We find that HIV-1 CA sequence, target cell-type, cell-cycle, CypA, and multiple Nups functionally interact in complex ways to affect the efficiency of viral infection and the antiviral activity of MX2. Indeed, we find that Nup levels (and presumably NPC composition) are quite variable in cell lines used to investigate HIV-1 infection, and also variable among NPCs within an individual cell. We further show, using biochemical assays that HIV-1 CA can bind to multiple Nups. Crucially, we show that diverse effects of CypA and CA mutations on infection, as well as MX2 antiviral activity, can be induced or abolished by altering Nup levels and NPC composition. Overall, our findings are consistent with a model in which several Nup-dependent ‘pathways’ to HIV-1 nuclear entry and integration exist. These pathways are differentially exploited by HIV-1 CA mutants and in different cell lines in a manner that is influenced by CypA. Moreover, these Nup-dependent nuclear entry pathways appear to be differently inhibited by MX2. Heterogeneity in Nup levels and or NPC composition likely, therefore, explains the differential dependence of HIV-1 on CA-binding cofactors and susceptibility to CA-binding antiviral proteins in different cellular contexts.

## Results

### HIV-1 infectivity and MX2 antiviral potency is influenced in complex ways by cell-type, cell-cycle, CypA, and viral capsid sequence

**Figure 1.**
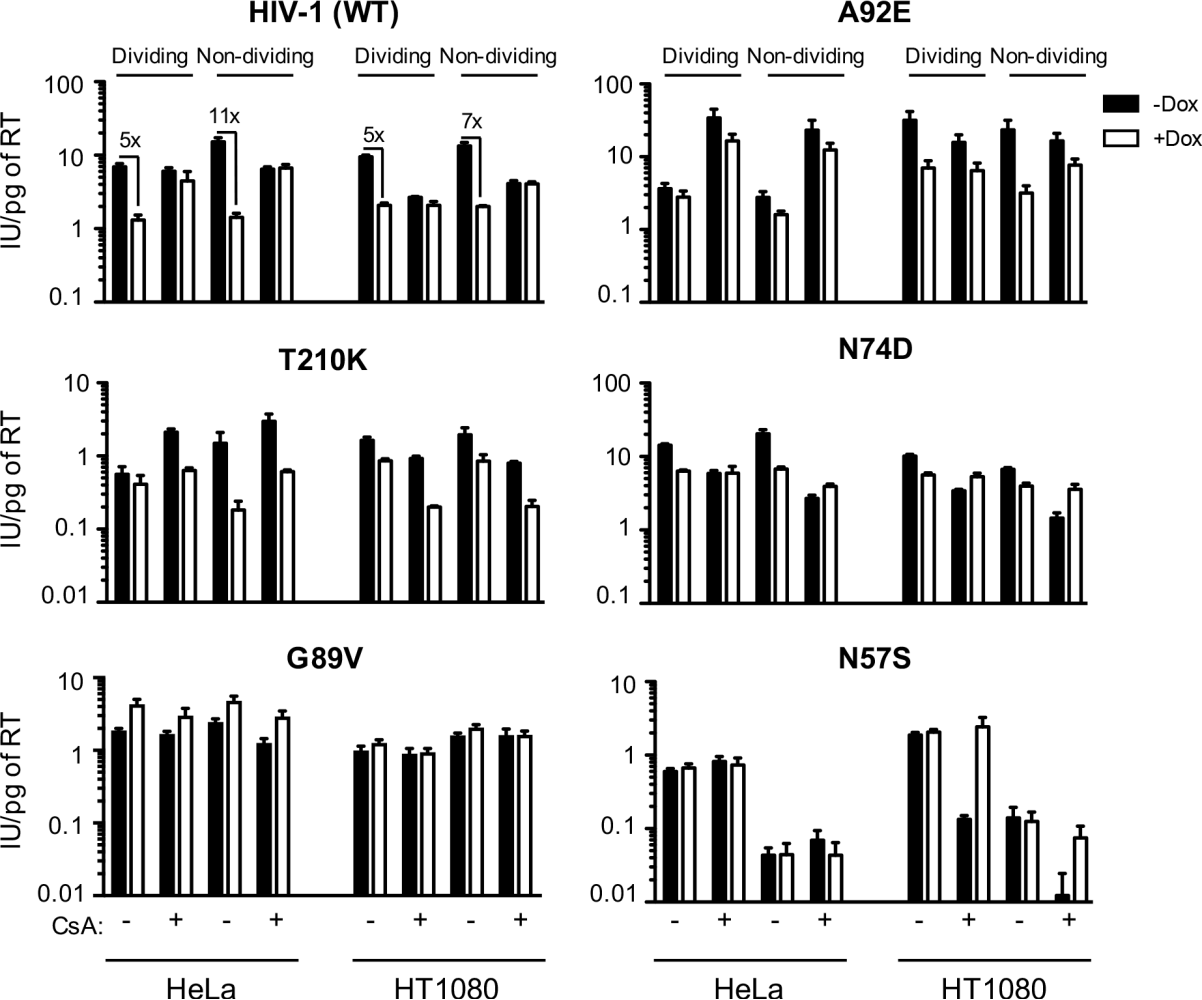
MX2 sensitivity is affected by the viral CA, the cell-cycle, and CypA/CsA in a cell-type dependent manner. Wild-type (WT) or CA-mutant HIV-1-GFP reporter virus infection of dividing or non-dividing (aphidicolin treated) HeLa or HT1080 cells expressing doxycycline-inducible MX2 in the presence (white bars) or absence (black bars) of pretreatment with doxycycline (Dox) and the presence or absence of CsA. Titers are represented as mean +sem of infectious units per pg of reverse transcriptase (RT), n ≥ 3 technical replicates, representative of five independent experiments.

For initial experiments to simultaneously assess the role of cell-type, CypA and cell-cycle in HIV-1 infection and its inhibition by MX2, we generated HeLa and HT1080 cell lines that expressed MX2 in a doxycycline-inducible manner and challenged these cells with HIV-1_WT_, derivatives encoding CA mutations (some of which had been selected for MX2-resistance), and other lentiviruses (Figure 1 and Figure 1—figure supplements 1 and 2). Cells were also treated with aphidicolin prior to infection to arrest the cell-cycle, and/or the CypA inhibitor CsA at the time of infection to abolish CypA:CA interactions. In a third cell line (HOS), MX2 was not well expressed using the inducible vector and therefore only effects of cell-cycle and CypA:CA interaction on infection were measured therein (Figure 1—figure supplement 3).

**Figure 1—figure supplement 1.**
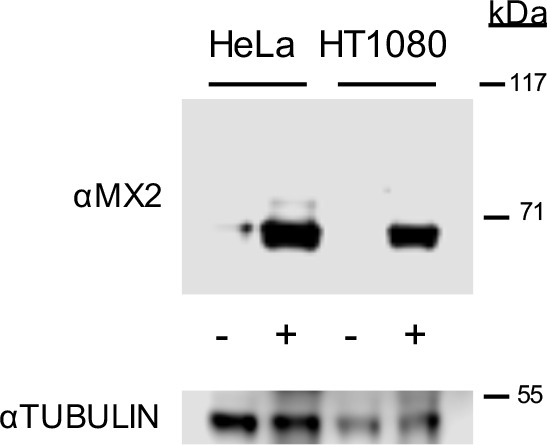
Western blot analysis of doxycycline inducible MX2 and tubulin loading control in HeLa and HT1080 cells.

**Figure 1—figure supplement 2.**
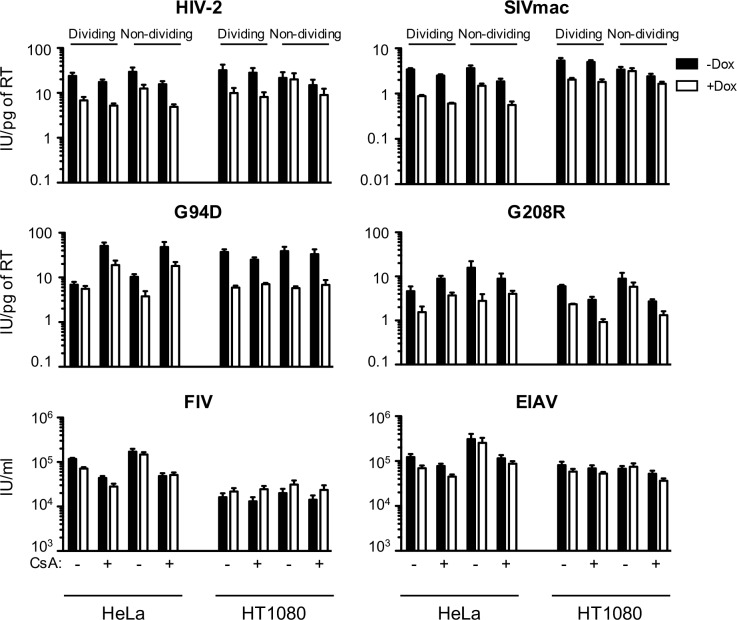
MX2 sensitivity is altered by the viral CA, the cell-cycle, and cyclophilin A in a cell-type dependent manner. Infection of dividing or non-dividing (aphidicolin treated) HeLa or HT1080 cells expressing doxycycline-inducible MX2 in the presence (white bars) or absence (black bars) of pretreatment with doxycycline (Dox) and the presence or absence of CsA with various GFP reporter viruses. Titers are represented as mean +sem of infectious units per pg of reverse transcriptase (RT) or infectious units per ml (for FIV and EIAV), n ≥ 3 technical replicates, representative of five independent experiments. FIV, feline immunodeficiency virus; EIAV, equine infectious anemia virus.

**Figure 1—figure supplement 3.**
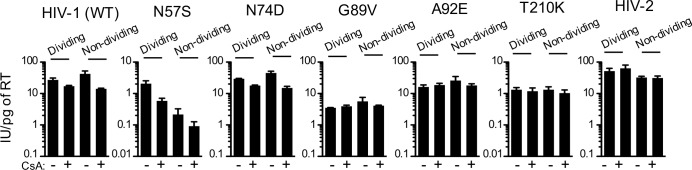
CsA sensitivity of HIV-1 CA mutants in HOS cells. WT, CA-mutant HIV-1, or HIV-2-GFP reporter virus infection of dividing or non-dividing (aphidicolin treated) HOS cells expressing doxycycline-inducible MX2 in the presence or absence of CsA. Titers are represented as mean +sem of infectious units per pg of reverse transcriptase (RT), n = 3 technical replicates, representative of four independent experiments.

For HIV-1_WT_, MX2 antiviral potency was enhanced by growth-arrest in HeLa cells, similar to our previous findings in HOS and K562 cells (Kane et al., 2013). However, growth-arrest caused only a marginal increase in MX2 potency in HT1080 cells. As expected from previous work (Hatziioannou et al., 2005; Sokolskaja et al., 2004), HIV-1_WT_ was largely unaffected by CsA addition in HeLa cells but its infectivity was reduced upon CsA addition in both HT1080 and HOS cells (Figure 1 and Figure 1—figure supplement 3). CsA addition abolished the anti-viral activity of MX2 in both HeLa and HT1080 cells, suggesting that MX2 is active only against CypA bound or modified HIV-1_WT_ capsids in these contexts.

HIV-2 and the simian immunodeficiency virus SIVmac, were less sensitive to MX2 in dividing HeLa, HOS, and HT1080 cells than HIV-1 ((Figure 1—figure supplement 2) and (Kane et al., 2013)). Moreover, and in contrast to effects on HIV-1_WT_, arresting cell division in HeLa, and especially in HT1080 cells, reduced rather than enhanced MX2 antiviral potency against HIV-2 and SIVmac. As expected, CsA addition (Figure 1—figure supplement 2) did not affect HIV-2 or SIV mac infection and did not affect MX2 potency against these viruses. Thus, the enhancing effects of cell-cycle arrest and CypA on MX2 activity were dependent on the incoming virus, and on the particular target cell line.

We next examined effects of MX2, cell cycle arrest, and CypA/CsA on HIV-1 infection using CA mutants with reported alterations in MX2 and CypA/CsA sensitivity. The HIV-1_G94D_ and HIV-1_A92E_ CA mutations confer CsA dependence (i.e. their infection is inhibited by CA:CypA binding) during early replication steps in some cell lines, including HeLa, but confer CsA resistance in others, including HOS (Hatziioannou et al., 2005; Sokolskaja et al., 2004) and HT1080 cells (Figure 1 and Figure 1—figure supplements 2 and 3). Both HIV-1_G94D_ and HIV-1_A92E_ exhibited reduced sensitivity to MX2 compared to HIV-1_WT_ in HeLa (Figure 1 and Figure 1—figure supplement 2) and HOS cells (Kane et al., 2013). Conversely, HIV-1_WT_, HIV-1_G94D_, and HIV-1_A92E_ exhibited equivalent MX2 sensitivity in HT1080 cells. Moreover, unlike HIV-1_WT_, the HIV-1_G94D_ and HIV-1_A92E_ mutants retained residual sensitivity to MX2 in the presence of CsA (Figure 1 and Figure 1—figure supplement 2).

The HIV-1_T210K_ CA mutant was selected by passage in MT4 cells expressing MX2 and exhibits resistance to inhibition by MX2 in that context (Busnadiego et al., 2014), HIV-1_T210K_ was similarly MX2-resistant in HeLa cells but, surprisingly, became sensitive to MX2 when HeLa cells were growth arrested. HIV-1_T210K_ was CsA-dependent, in that it was rendered more infectious upon CsA addition in HeLa cells, but not in HT1080 or HOS cells. However, unlike HIV-1_WT_, CsA addition conferred increased sensitivity of HIV-1_T210K_ to MX2 in both HeLa and HT1080 cells (Figure 1 and Figure 1—figure supplement 2). Thus, although the HIV-1_T210K_ mutant arose under selective pressure from MX2, the acquired resistance is cell-type-, cell-cycle-, and CypA-dependent. Similarly, the G208R mutant, which also arose under selective pressure imposed by MX2 in MT4 cells (Busnadiego et al., 2014) was only partly resistant to MX2 in HeLa or HT1080 cells (Figure 1—figure supplement 2).

HIV-1_N74D_, whose CA does not interact with CPSF6 (Lee et al., 2010), was less sensitive than HIV-1_WT_ to MX2 in HeLa and HT1080 cells (as well as in HOS cells (Goujon et al., 2013; Kane et al., 2013)). HIV-1_N74D_ was also CsA sensitive in all three cell lines (Ambrose et al., 2012), particularly when cells were growth arrested. However, CsA treatment abolished the MX2 sensitivity of HIV-1_N74D_, and even caused MX2 to modestly increase HIV-1_N74D_ infection, particularly in non-dividing HT1080 cells (Figure 1). Modest enhancement of infection by MX2 was also observed for the HIV-1_G89V_ whose capsid does not bind CypA (Wiegers et al., 1999), in both HeLa and HT1080 cells, and irrespective of cell-division (Figure 1).

A particularly striking phenotype was observed with the HIV-1_N57S_ CA mutant, which is unable to efficiently infect non-dividing cells (Rihn et al., 2013) and is MX2 resistant. While HIV-1_N57S_ infection was unaffected by either CsA or MX2 in HeLa cells, HIV-1_N57S_ was exquisitely sensitive to CsA (~20 fold inhibition) in HT1080 cells. Remarkably, this dramatic reduction in infectivity was completely reversed by MX2. In other words, MX2 increased infection by the HIV-1_N57S_ mutant when CypA:CA interaction was inhibited (Figure 1).

These experiments demonstrate that MX2 and CypA can either inhibit or enhance HIV-1 infection. Their effects clearly varied in a cell-type, cell-cycle and CA-sequence dependent manner. Thus, both the viral capsid, and some feature of the cellular environment that varies in the aforementioned cell lines, presumably other cellular factor(s), determine whether MX2 and CypA inhibit or enhance HIV-1 infection.

### Variation in Nup expression among cell types

Both CA sequence and CypA have been reported to affect the requirement for particular Nups during HIV-1 infection (Matreyek and Engelman, 2011; 2013). Moreover, MX2 localizes to nuclear pores and appears to act at the nuclear entry step of HIV-1 infection. Mutants of MX2 have also been reported to broadly affect nuclear import (King et al., 2004). For these reasons and because the nuclear envelope fragments during cell division, we next considered whether the varied effects of CA sequence, MX2, CypA, cell-type and cell-cycle on HIV-1 infection might be mediated through Nups or NTRs.

**Figure 2.**
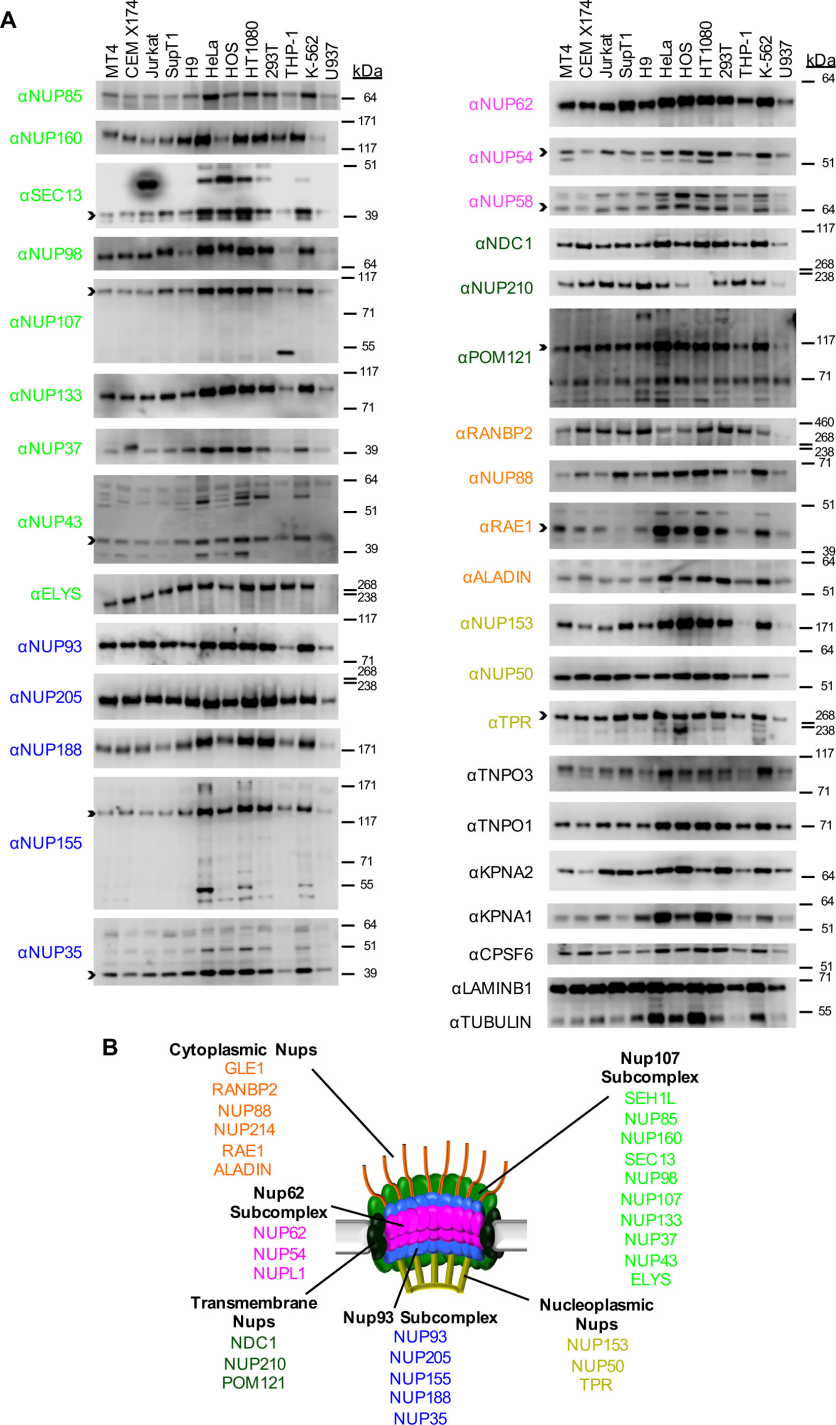
Nup and NTR expression in immortalized cell lines. (**A**) Western blot analysis of expression of Nups (color-coded by subcomplex as indicated in panel B), selected NTRs, and CPSF6 in T cell (MT4, CEM X174, Jurkat, SupT1, H9), adherent cell (HeLa, HOS, HT1080, 293T), or monocytic cell (THP-1, K-562, U937) lines. Where with multiple bands were detected, arrowheads indicate the band whose migration most closely matched the predicted molecular weight of the Nup/NTR. The indicated bands were utilized for quantification in Figure 2—figure supplement 1, and correspond to the band highlighted in Figure 5. Each well was loaded with 10 μL of lysate containing 10^4^ cells. Each blot represents one of at least three replicates produced from two or three separately generated cell lysates. (**B**) Schematic representation of the nuclear pore complex listing individual members of each subcomplex, color-coded to correspond to the labels for the western blot in panel (**A**).

To begin to examine whether Nups or NTRs might govern the differential effects of the aforementioned factors on HIV-1 infection, we examined the levels of each Nup and a subset of NTRs in a panel of cell lines, including a number of T-cell derived, monocyte derived, and adherent cell lines commonly utilized in HIV-1 research. Certain Nups (SEH1, GLE1, and NUP214) that we were unable to detect by western blotting were excluded from this analysis. We found several striking differences in the levels of individual Nups among this panel of cell lines (Figure 2 and Figure 2—figure supplement 1). For example, the monocytic THP-1 and U937 cells expressed lower levels of almost all Nups than other cell lines, suggesting that they may have fewer nuclear pores. Additionally, HT1080 cells did not express detectable levels of NUP210. We also found that natural target cells of HIV-1 infection, namely primary CD4^+^ T cells and macrophages exhibited differences in nucleoporin expression levels (Figure 2—figure supplements 2 and 3). For example, dramatically higher levels of NUP37 were found in primary macrophages as compared to MT4, THP-1, and primary CD4^+^ T cells, while lower levels of NUP210 were present in macrophages. The appearance of unanticipated anti-Nup antibody reactive species in some cell lines and in primary cells suggested potential differences in the expression of alternative isoforms of several Nups (Figure 2 and Figure 2—figure supplement 2). However, this cannot be definitely determined without more extensive investigation, as non-specific binding by the Nup-specific antibodies may underlie this variability. These results are consistent with previous studies reporting cell-type dependent differences in Nup expression (D'Angelo et al., 2009; Gomez-Cavazos and Hetzer, 2015; Lowe et al., 2015; Lupu et al., 2008; Ori et al., 2013) and suggest that marked compositional heterogeneity of NPCs occurs between cell types. Alternatively, it is possible that the fraction of each Nup protein that is associated with nuclear pores varies with cell type. In such a scenario the capsids could interact with Nups in the cytoplasm, and both the NPC-associated and cytoplasmic forms of a given Nup could have an effect on viral infection. In any case, variation in Nup levels has the potential to affect HIV-1 infection and its modulation by cell-cycle, CypA, and MX2.

**Figure 2—figure supplement 1.**
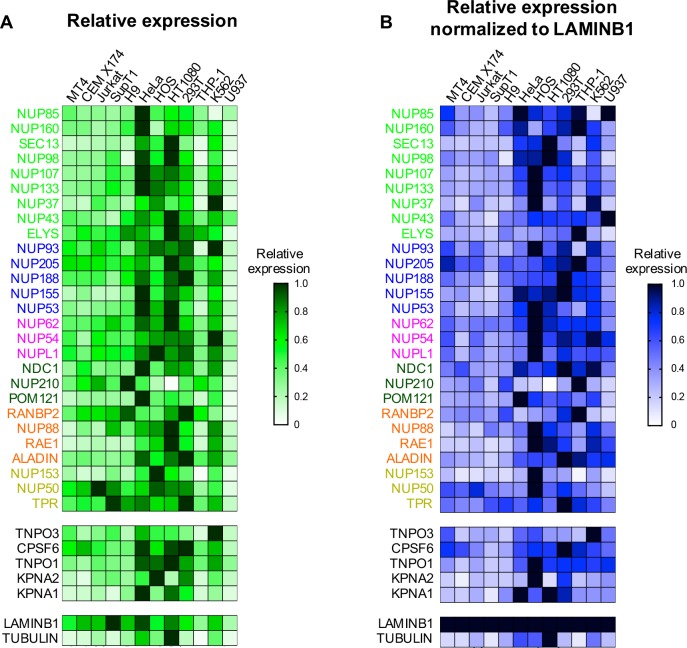
Nup and NTR expression in immortalized cell lines. Heat map representing Nup, selected NTRs, and CPSF6 protein levels in T cell (MT4, CEM X174, Jurkat, SupT1, ) adherent cell (HeLa, HOS, HT1080, 293T), or monocytic cell (THP-1, K-562, U937) lines (color-coded by subcomplex as in Figure 2) from the individual blots shown in Figure 2 (n = 1). Heat maps are normalized for each protein such that the highest expressing cell line is assigned a value of 1.0 (black), while undetectable expression is assigned a zero value (white). (**B**) Heat map representing protein levels from the blots shown in Figure 2 normalized to LAMIN B1 expression (determined by dividing Nup/NTR expression by LAMIN B1 expression and assigning the maximum for each Nup/NTR a value of 1.0).

**Figure 2—figure supplement 2.**
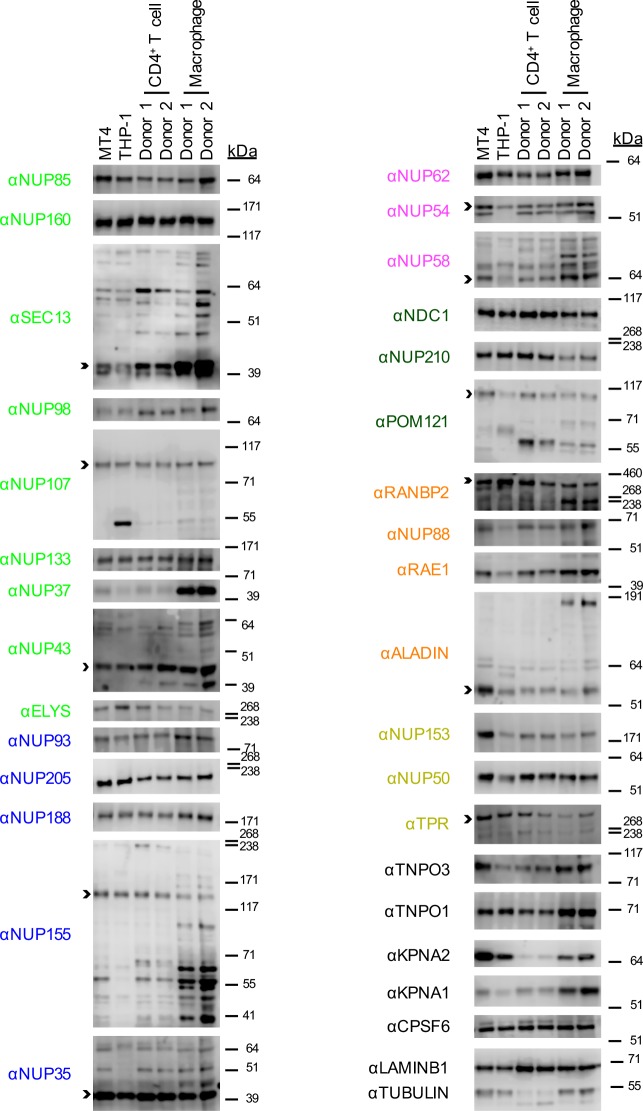
Nup and NTR expression in primary cells. Western blot analysis of expression of Nups, selected NTRs, and CPSF6 in MT4, THP-1, primary CD4^+^ T cells, and macrophages from two donors (color coded by subcomplex as in Figure 2B). Arrowheads indicate the band whose migration most closely matched predicted molecular weight of the Nup/NTR. The indicated bands were utilized for quantification in Figure 2—figure supplement 2 and correspond to the band highlighted in Figure 5. Each blot represents one of at least two replicates.

**Figure 2—figure supplement 3.**
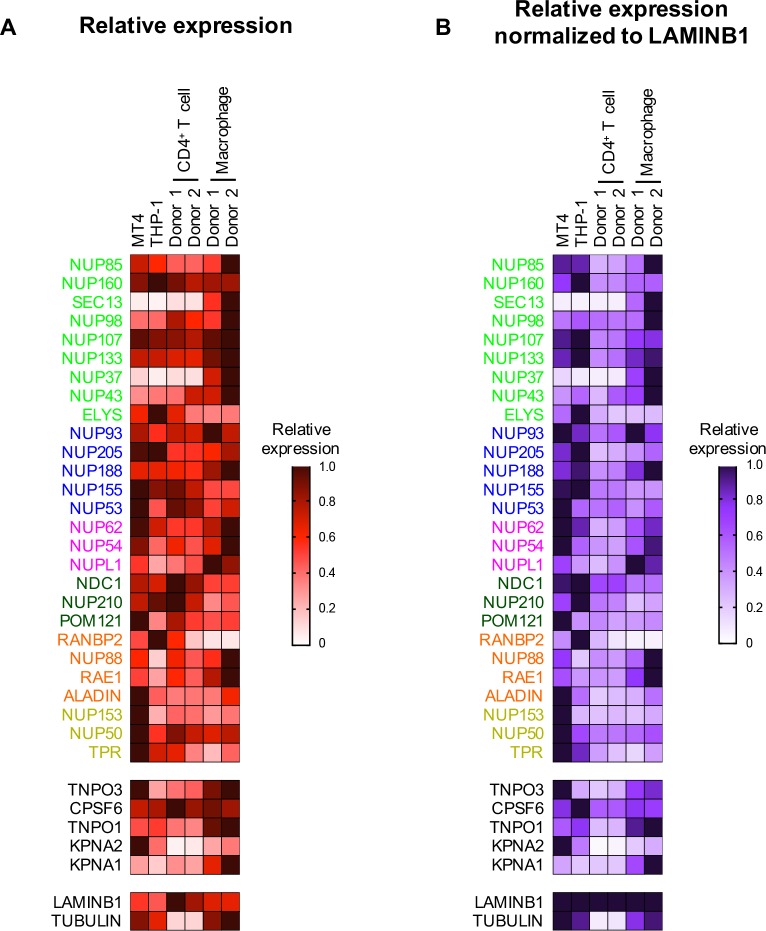
Nup and NTR expression in primary cells. (**A**) Heat map representing Nup, selected NTRs, and CPSF6 protein levels in MT4, THP-1, primary CD4^+^ T cells, and macrophages from two donors (color coded by subcomplex as in Figure 2) from the individual blots shown in Figure 2—figure supplement 2 (n = 1). Heat maps are normalized for each protein such that the highest expressing cell line is assigned a value of 1.0 (black), while undetectable expression is assigned a zero value (white). (**B**) Heat map representing protein levels from the blots shown in Figure 2—figure supplement 2 normalized to LAMIN B1 expression (determined by dividing Nup/NTR expression by LAMIN B1 expression and assigning the maximum for each Nup/NTR a value of 1.0).

### Multiple Nups bind HIV-1 CA tubes in vitro

**Figure 3.**
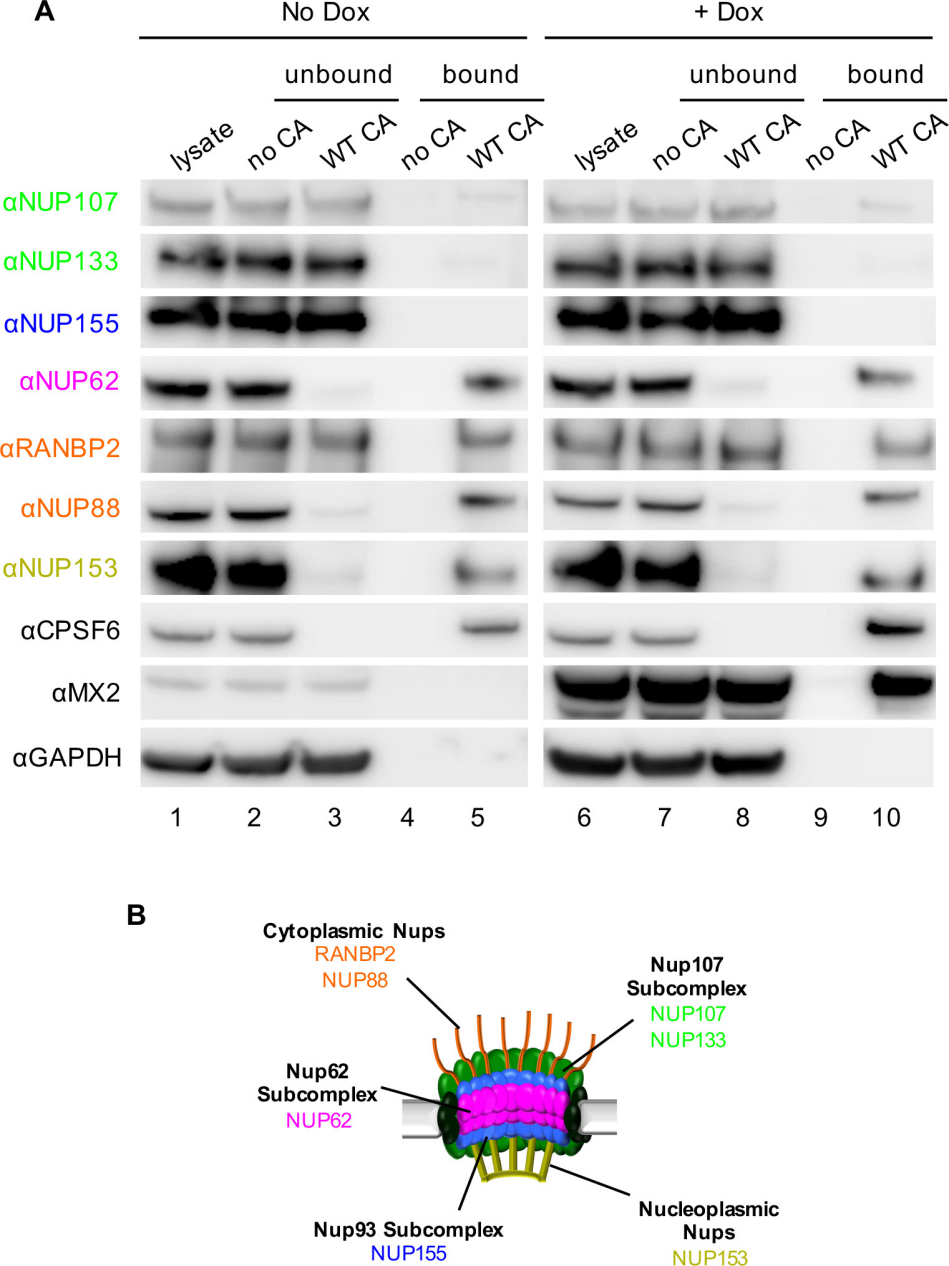
Interactions between CA tubes and cellular Nups. (**A**) CA tubes were assembled in vitro and incubated with lysates of HeLa cells without (lanes 1–5) or with (lanes 6–10) Dox dependent expression of MX2. The reaction mixtures were subjected to centrifugation to separate pulled-down (or bound) fractions from supernatant (unbound) proteins. Lanes 1 and 6: cellular lysates; Lanes 2 and 7: supernatants from control experiments without CA tubes; Lanes 3 and 8: supernatants after incubating cellular lysates with CA tubes; Lanes 4 and 9: pulled-down fractions from control experiments in the absence of CA tubes; Lanes 5 and 10: proteins bound to CA tubes. Bands shown in each blot correspond to those indicated in Figure 2. Representative of two independent experiments. (**B**) Schematic representation of the nuclear pore complex listing individual members of each subcomplex tested in (**A**).

Nups have been found to interact with the HIV-1 CA in genetic assays, and NUP153 and NUP98 have been shown to bind to assembled CA-nucleocapsid (NC) tubes in vitro (Di Nunzio et al., 2013; Matreyek et al., 2013). In order to monitor binding interactions between CA tubes and Nups, we tested binding to CA nanotubes assembled in high salt, as done previously for CypA (Liu et al., 2016). These viral capsid-mimics were then incubated with lysates of HeLa cells, which contained matching NaCl concentrations to maintain the integrity of CA tubes during the binding assay. Subsequent centrifugation allowed us to separate CA tubes together with cognate binding partners in pelleted fractions from unbound cellular proteins and monomeric CA in supernatants (Figure 3 and Figure 3—figure supplement 1A). Indeed, comparison of unbound versus bound fractions revealed that the vast majority of cellular proteins were not pulled-down by CA tubes (Figure 3—figure supplement 1A). Additionally, the failure of CA tubes containing the N74D mutation to bind to CPSF6 (Figure 3—figure supplement 1B) further indicates that the assay detects specific protein-protein interactions. We used western blot assays to test for interactions between CA tubes and CA tube binding partners (CPSF6 and MX2) as well as representative Nups (NUP107, NUP133, NUP155, NUP62, RANBP2, NUP88, NUP153)

**Figure 3—figure supplement 1.**
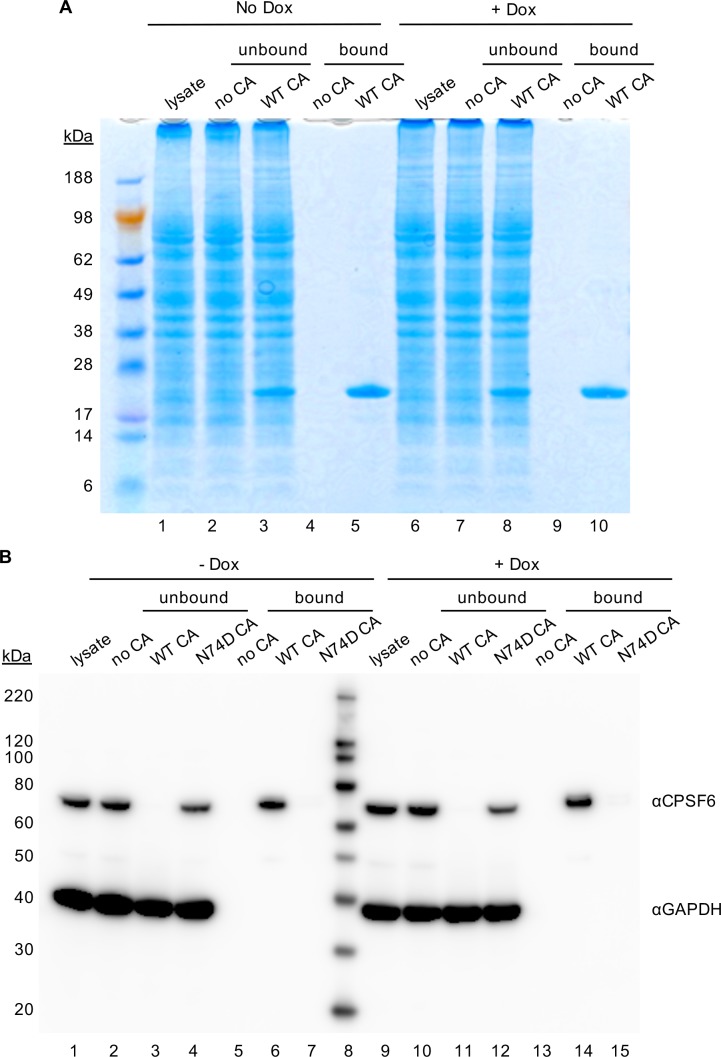
Specificity of CA binding assay. (**A**) Coomassie stained SDS-PAGE image for pull-down experiments with in vitro assembled CA tubes and HeLa cell lysates. Lanes 1–10 correspond to those in Figure 3. In addition, molecular weight markers are shown. Representative of two independent experiments. (**B**) WT and N74D CA tubes were assembled in vitro and incubated with lysates of HeLa cells without (lanes 1–7) or with (lanes 9–15) Dox dependent expression of MX2. The reaction mixtures were subjected to centrifugation to separate pulled-down (or bound) fractions from supernatant (unbound) proteins. Lanes 1 and 9: cellular lysates; Lanes 2 and 10: supernatants from control experiments without CA tubes; Lanes 3 and 11: supernatants after incubating cellular lysates with WT CA tubes; Lanes 4 and 12: supernatants after incubating cellular lysates with N74D CA tubes; Lanes 5 and 13: pulled-down fractions from control experiments in the absence of CA tubes; Lanes 6 and 14: CPSF6 bound to WT CA tubes; Lanes 7 and 15: No CPSF6 bound to N74D CA tubes. Representative of two independent experiments.

Notably, CPSF6 was detected in the CA-tube bound fractions (lane 5 Figure 3) and, in fact, was entirely depleted from the cellular lysates by WT CA tubes (compare lanes 2 and 3, Figure 3). Similarly, almost complete depletion from cell lysates was observed for NUP62, NUP88, and NUP153, suggesting that these proteins are tightly bound to the CA tubes. RANBP2 was also bound to CA tubes, but a significant fraction of this protein remained in the unbound supernatant. It should be noted that there are numerous interactions between individual Nups; therefore, binding to CA tubes by individual Nups in this assay may not necessarily reflect direct binding in all cases. Nevertheless, not all Nups interacted with CA tubes, as little or no binding was detected for NUP107, NUP133, and NUP155. Finally, expression of MX2 following doxycycline addition also confirmed a robust interaction between MX2 and the viral CA tubes (lane 10, Figure 3). MX2 expression did not detectably affect the interaction between Nups and CA tubes (compare lanes 5 and 10, Figure 3). However, because the CA tubes are in large excess in this assay, this finding does not exclude the possibility that MX2 could inhibit infection by competitively inhibiting interactions between CA and Nups.

### Effects of depleting individual Nups on NPC integrity

**Figure 4.**
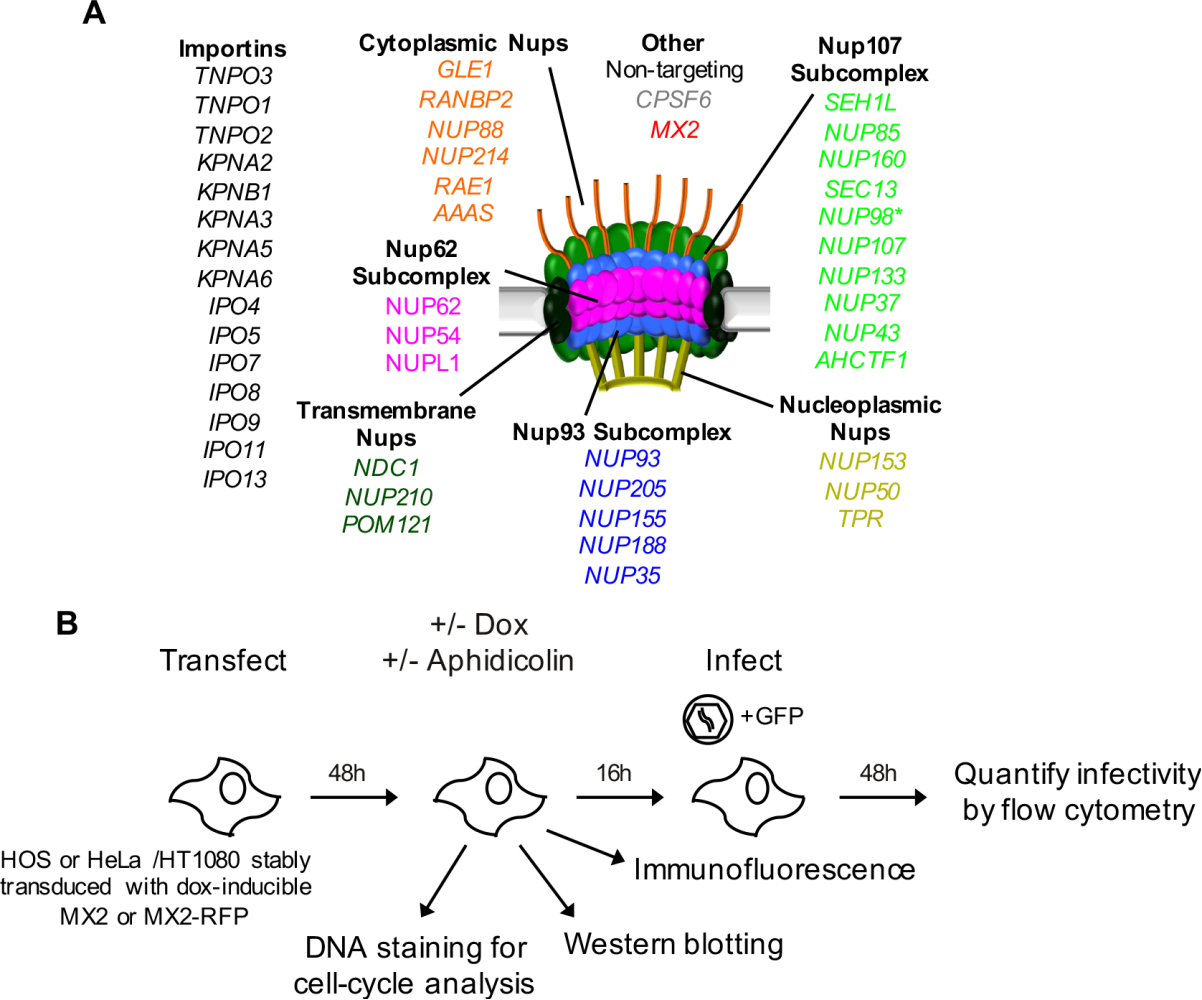
An siRNA-based knockdown approach to investigate the role of Nups and NTRs in MX2 localization and anti-viral activity. (**A**) Schematic representation of the nuclear pore complex and genes included in siRNA library color coded by subcomplex. (*NUP98 is listed as a member of Nup107 subcomplex, however. NUP98 and NUP96 are produced following autoproteolytic cleavage of a polyprotein precursor (Fontoura et al., 1999; Rosenblum and Blobel, 1999), the siRNA used herein targets both Nups). Importins/nuclear transport receptors (NTRs) included in the siRNA library are listed in black. Also included, siRNA targeting MX2 or CPSF6 and a non-targeting control siRNA. (**B**) Experimental strategy to investigate the roles of Nups and NTRs involved in HIV-1 infection, MX2 subcellular localization, and anti-viral activity of MX2. For a detailed description, refer to the Materials and methods.

**Figure 5.**
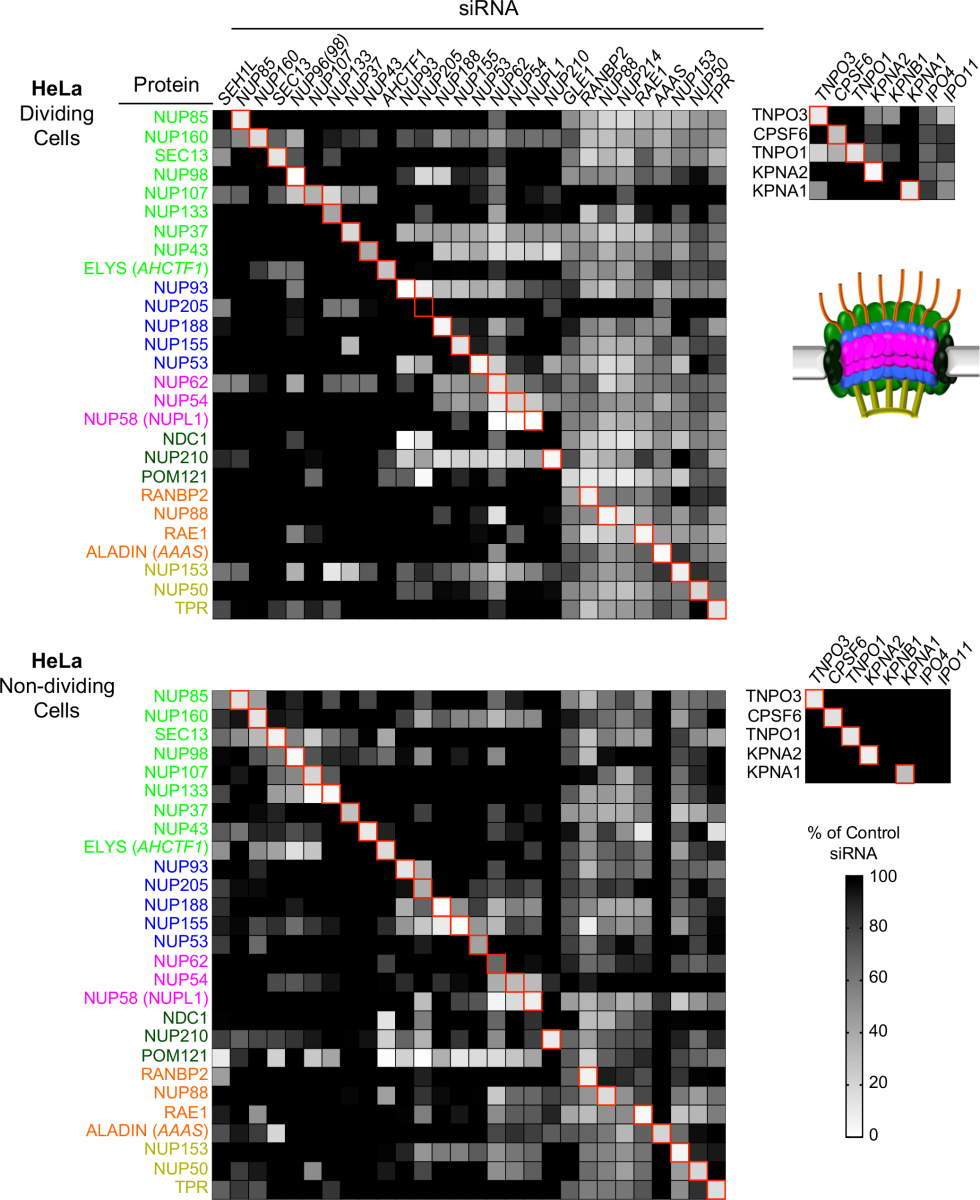
Efficient siRNA-mediated knockdown of Nups and pleiotropic effects of Nup depletion in HeLa cells. Heat map representing Nup and NTR protein levels (color coded by subcomplex as in Figure 4A and according to the included schematic) in dividing (top) and non-dividing (bottom) HeLa cells 64 hr after transfection with the indicated siRNA. Protein levels are expressed as ratios of Nup/NTR expression:LAMIN B1 expression, based on the blots shown in Figure 5—figure supplement 3 and 4 normalized to control siRNA transfected cells that were assigned a value of 1.0 (black). Reduced expression is indicated in gray-white, with no detectable expression assigned a zero value (white). Red boxes highlight corresponding antibody-siRNA pairs.

We next sought to determine the importance of Nups and NTRs on HIV-1 infection and the antiviral activity of MX2 in variable cell contexts. To accomplish this, we used a panel of siRNAs targeting human Nups and NTRs, as well as CPSF6 and MX2 as controls (Figure 4A–B). We used HeLa, HT1080, and HOS cells in these experiments because (i) there were notable differences between them in the effects of cell cycle, CsA and CA mutations on HIV-1 infection (Figure 1 and Figure 1—figure supplements 2 and 3) and (ii) siRNA transfection and knockdown was efficient in these cells. Cells were transfected with siRNAs before being split into replicate wells for MX2 induction (doxycycline treatment) and growth arrest (aphidicolin treatment). The cells in one replicate well were subjected to western blot analysis to confirm that knockdown efficiency was the same for each condition in each experiment. Overall, Nup and NTR depletion was efficient in all cell lines, including under conditions of growth arrest, with a few exceptions (Figure 5 and Figure 5—figure supplements 1–6). Nups whose protein levels were not reduced to <30% of control levels following siRNA transfection were excluded from subsequent analyses.

**Figure 5—figure supplement 1.**
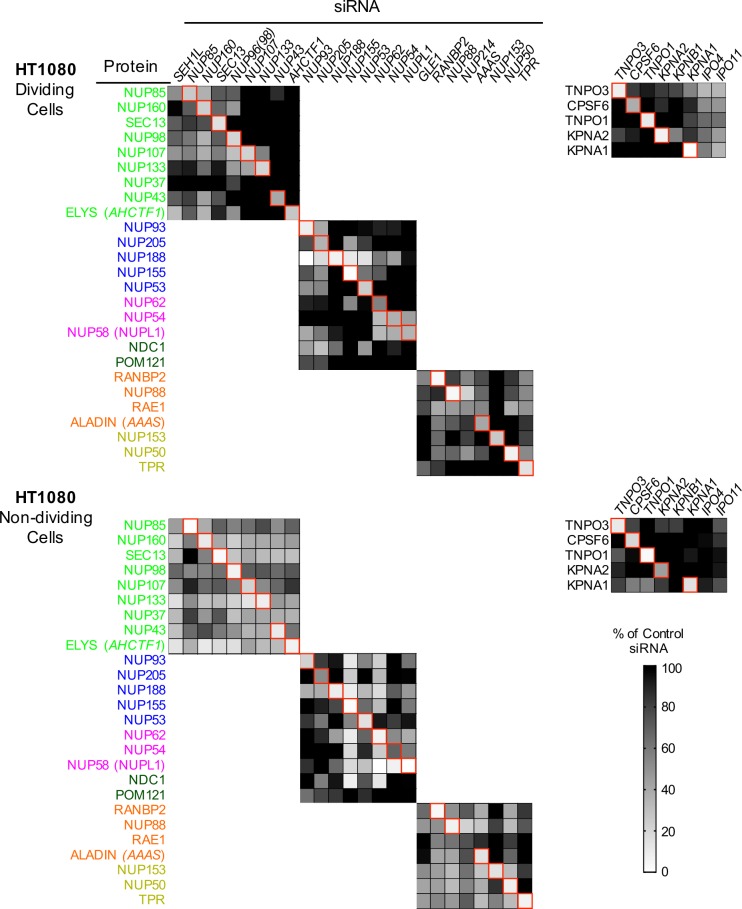
Efficient siRNA-mediated knockdown of Nups and pleiotropic effects of Nup depletion in HT1080 cells. Heat map representing protein levels of Nups or NTRs (color coded by subcomplex as in Figure 4A) in dividing (top) and non-dividing (bottom) HT1080 cells 64 hr after transfection with the indicated siRNA. Protein levels are expressed as ratios of Nup/NTR expression:LAMIN B1 expression, based on the blots shown in Figure 5—figure supplement 5, normalized to control siRNA transfected cells that were assigned a value of 1.0 (black). Reduced expression is indicated in gray-white, with no detectable expression assigned a zero value (white). Red boxes highlight corresponding antibody-siRNA pairs.

**Figure 5—figure supplement 2.**
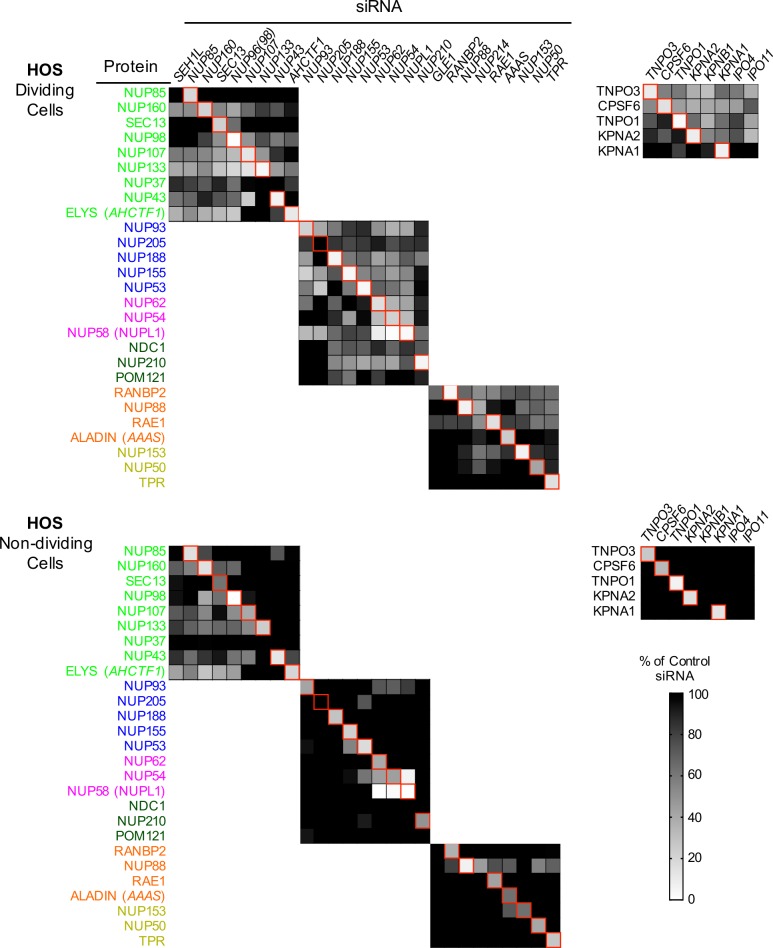
Efficient siRNA-mediated knockdown of Nups and pleiotropic effects of Nup depletion in HOS cells. Heat map representing protein levels of Nups or NTRs (color coded by subcomplex as in Figure 4A) in dividing (top) and non-dividing (bottom) HOS cells 64 hr after transfection with the indicated siRNA. Protein levels are expressed as ratios of Nup/NTR expression:LAMIN B1 expression, based on the blots shown in Figure 5—figure supplement 6, normalized to control siRNA transfected cells that were assigned a value of 1.0 (black). Reduced expression is indicated in gray-white, with no detectable expression assigned a zero value (white). Red boxes highlight corresponding antibody-siRNA pairs.

**Figure 5—figure supplement 3.**
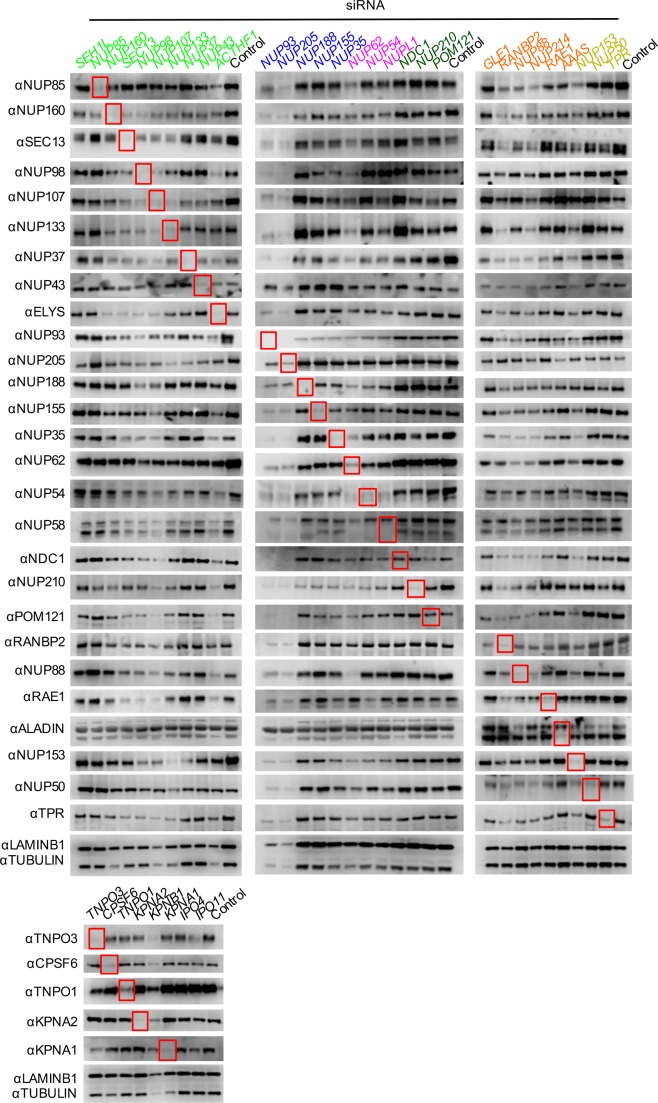
Knockdown of Nups and NTRs in dividing HeLa cells. Western blot analysis of Nup and NTR expression levels in HeLa cells stably transduced with doxycycline-inducible MX2 64 hr after transfection with the indicated siRNA color coded by subcomplex as in Figure 4A. Red boxes highlight lanes for corresponding antibody-siRNA pairs. Bands for each Nup/NTR correspond to those indicated in Figure 2. Each blot was generated from an experiment in which the effect of siRNA knockdown on the subcellular localization of MX2 and Nups, as well as on WT HIV-1 infection was similar to that shown below. The complete array of blots was conducted once, although randomly selected blotting experiments carried out in triplicate (approximately 10), as well as infectivity data indicated both consistent levels of knockdown and pleiotropic effects across experiments.

**Figure 5—figure supplement 4.**
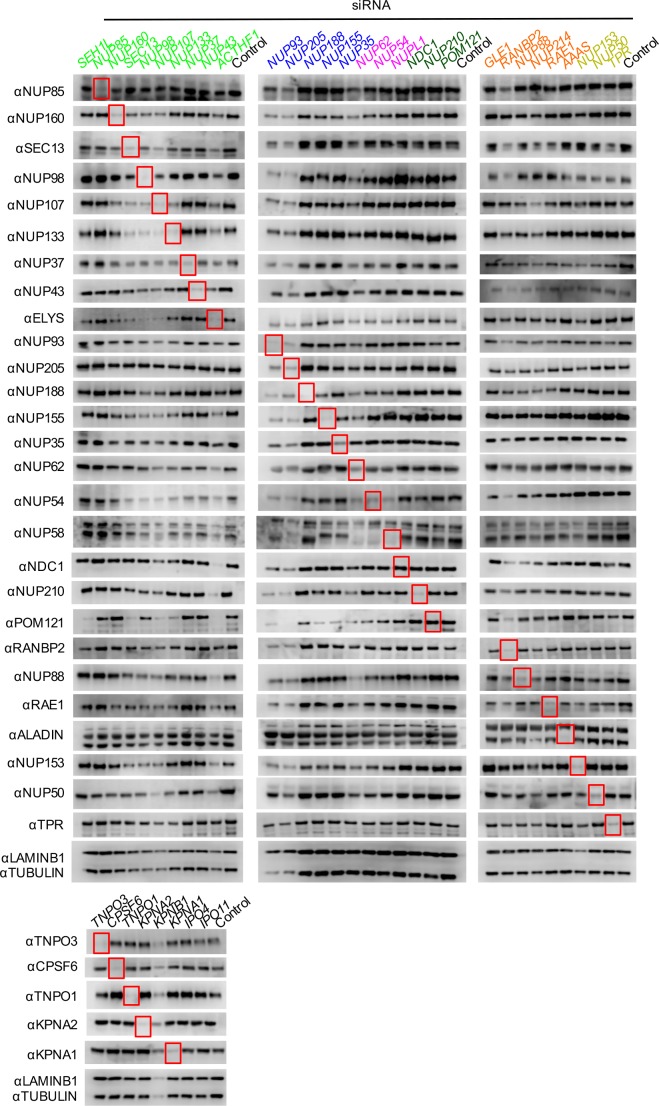
Knockdown of Nups and NTRs in non-dividing HeLa cells. Western blot analysis of Nup and NTR expression levels in growth arrested (aphidicolin treated) HeLa cells stably transduced with doxycycline-inducible MX2 64 hr after transfection with the indicated siRNA color coded by subcomplex as in Figure 4A. Red boxes highlight lanes for corresponding antibody-siRNA pairs. Bands for each Nup/NTR correspond to those indicated in Figure 2. Each blot is generated from an experiment in which the effect of siRNA knockdown on WT HIV-1 infection was similar to that shown in . The complete array of blots was conducted once, although randomly selected blotting experiments carried out in triplicate (approximately 10), as well as infectivity data indicated both consistent levels of knockdown and pleiotropic effects across experiments.

**Figure 5—figure supplement 5.**
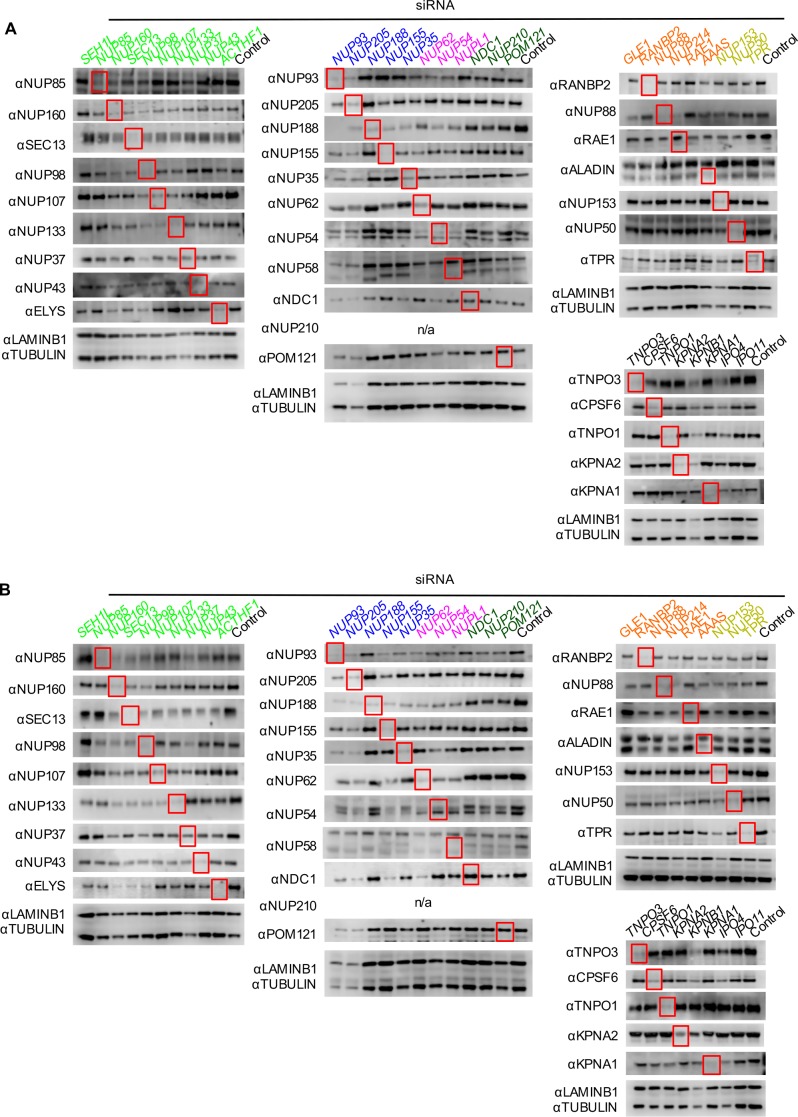
Knockdown of Nups and NTRs in HT1080 cells. Western blot analysis of Nup and NTR expression levels in A) dividing and B) non-dividing (aphidicolin treated) HT1080 cells stably transduced with doxycycline-inducible MX2 64 hr after transfection with the indicated siRNA color coded by subcomplex as in Figure 4A. Red boxes highlight lanes for corresponding antibody-siRNA pairs. (n/a – not tested since HT1080 cells do not express NUP210, see Figure 2—figure supplement 1). Bands for each blot correspond to those indicated in Figure 2. Each blot was generated from an experiment in which the effect of siRNA knockdown on the subcellular localization of MX2 and Nups, as well as on WT HIV-1 infection was similar to that shown below. The complete array of blots was conducted once, although blotting experiments carried out in triplicate (approximately 5), as well as infectivity data indicated both consistent levels of knockdown and pleiotropic effects across experiments.

**Figure 5—figure supplement 6.**
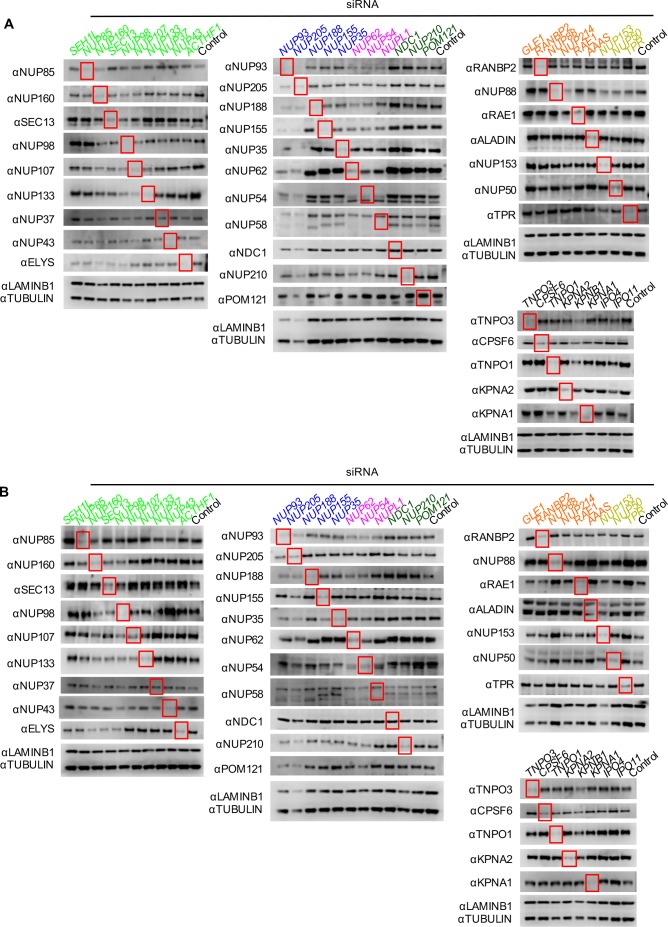
Knockdown of Nups and NTRs in HOS cells. Western blot analysis of Nup and NTR expression levels in A) dividing and B) non-dividing (aphidicolin treated) HOS cells 64 hr after transfection with the indicated siRNA color coded by subcomplex as in Figure 4A. Red boxes highlight lanes for corresponding antibody-siRNA pairs. Bands for each blot correspond to those indicated in Figure 2. Each blot was generated from an experiment in which the effect of siRNA knockdown on WT HIV-1 infection was similar to that shown below. The complete array of blots was conducted once, although blotting experiments carried out in triplicate (approximately 5), as well as infectivity data indicated both consistent levels of knockdown and pleiotropic effects across experiments.

**Figure 5—figure supplement 7.**
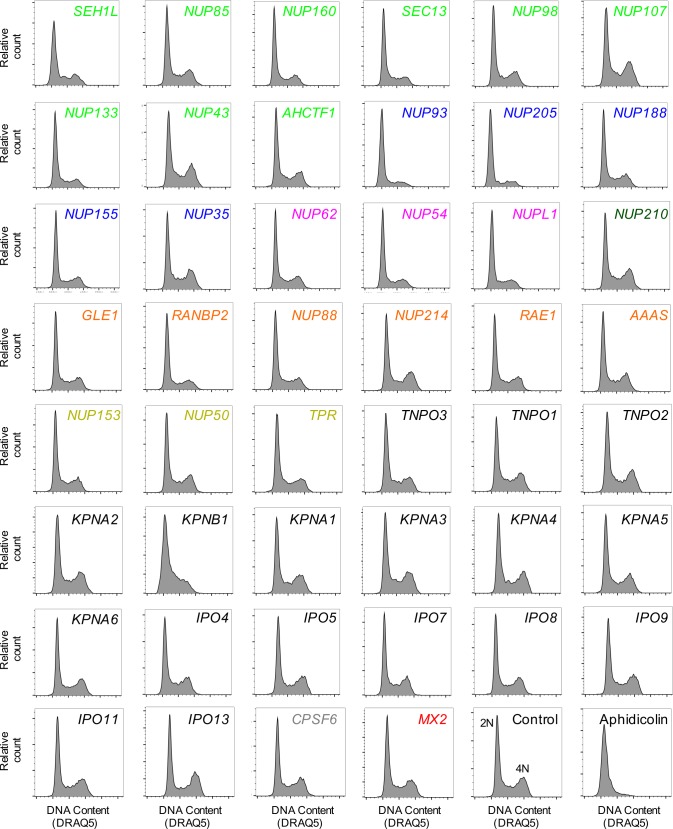
Cell-cycle profile of HOS cells following transfection with Nup/NTR targeting siRNA. Cell-cycle profile of HOS cells 64 hr after transfection with the indicated siRNA or following aphidicolin treatment, color coded by subcomplex as in Figure 4A. DNA content was determined by flow cytometry following staining with the DNA dye DRAQ5. For each plot, 3000–12000 events were collected. Representative of two independent experiments. 2N and 4N DNA content are indicated for the Control siRNA transfected sample.

**Figure 5—figure supplement 8.**
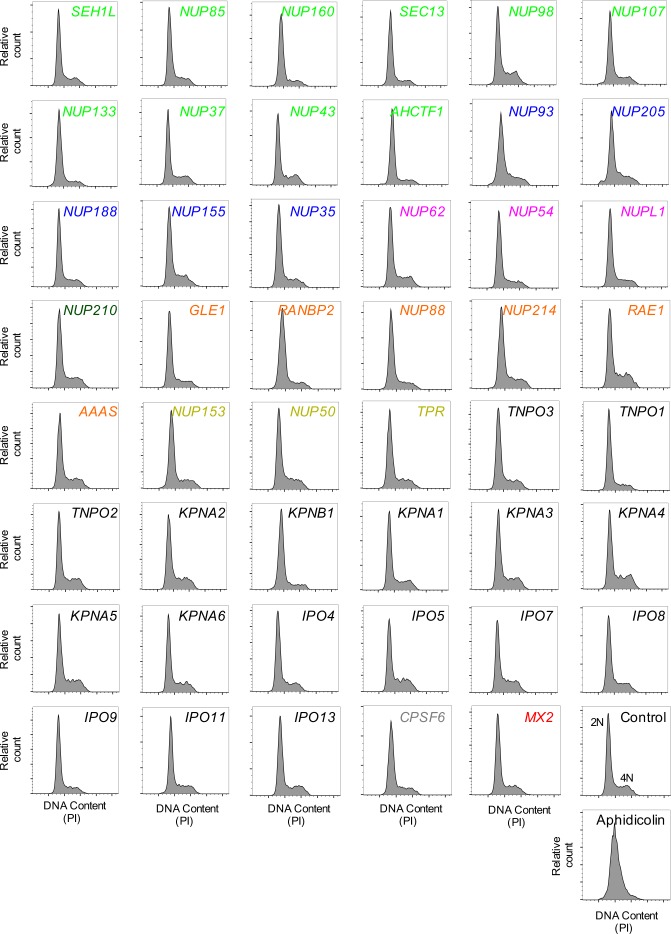
Cell-cycle profile of HeLa cells following transfection with Nup/NTR targeting siRNA. Cell-cycle profile of HeLa cells 64 hr after transfection with the indicated siRNA or following aphidicolin treatment, color coded by subcomplex as in Figure 4A. DNA content was determined by flow cytometry following staining with the DNA dye propidium iodide (PI). For each plot, 3000–12000 events per sample were collected. Representative of two independent experiments. 2N and 4N DNA content are indicated for the Control siRNA transfected sample.

To monitor potential pleiotropic effects following Nup depletions we examined the levels of Nups other than the one targeted by each specific siRNA. In the case of HeLa cells this analysis was done for all Nups for which antibodies were available. In the case of HT1080 and HOS cells, we monitored pleiotropic effects on Nups in the same subcomplex as the siRNA target. For the purposes of this analysis, the Nup93 subcomplex, the Nup62 subcomplex, the transmembrane subcomplexes, as well as the cytoplasmic and nuclear basket Nups were considered separate subcomplexes. We found quite dramatic changes in the levels of Nups other than the targeted Nup that varied depending on cell-type and cell-cycle (Figure 5 and Figure 5—figure supplements 1–6). The widespread pleiotropic effects of Nup or NTR depletion likely reflect the remarkable complexity of nuclear pore structure and formation, and the tight transcriptional, post-transcriptional, and post-translational controls of Nup expression levels [reviewed in (Doucet and Hetzer, 2010). We additionally assessed the impact of Nup or NTR depletion on cellular physiology by DNA content mediated cell-cycle analysis in HOS and HeLa cells (HT1080 cells were not amenable to reproducible measurements of DNA content) (Figure 5—figure supplements 7 and 8). While a few Nup/NTR depletions affected the cell-cycle profile, decreasing the proportion of cells in G2 (in particular NUP93, NUP205, and KPNB1), and decreased the overall levels of nucleoporins, most knockdowns did not affect the cell cycle, and none caused complete growth arrest, suggesting that at least at the time of infection, cell physiology was not grossly disrupted by Nup/NTR depletion, in most cases.

**Figure 6.**
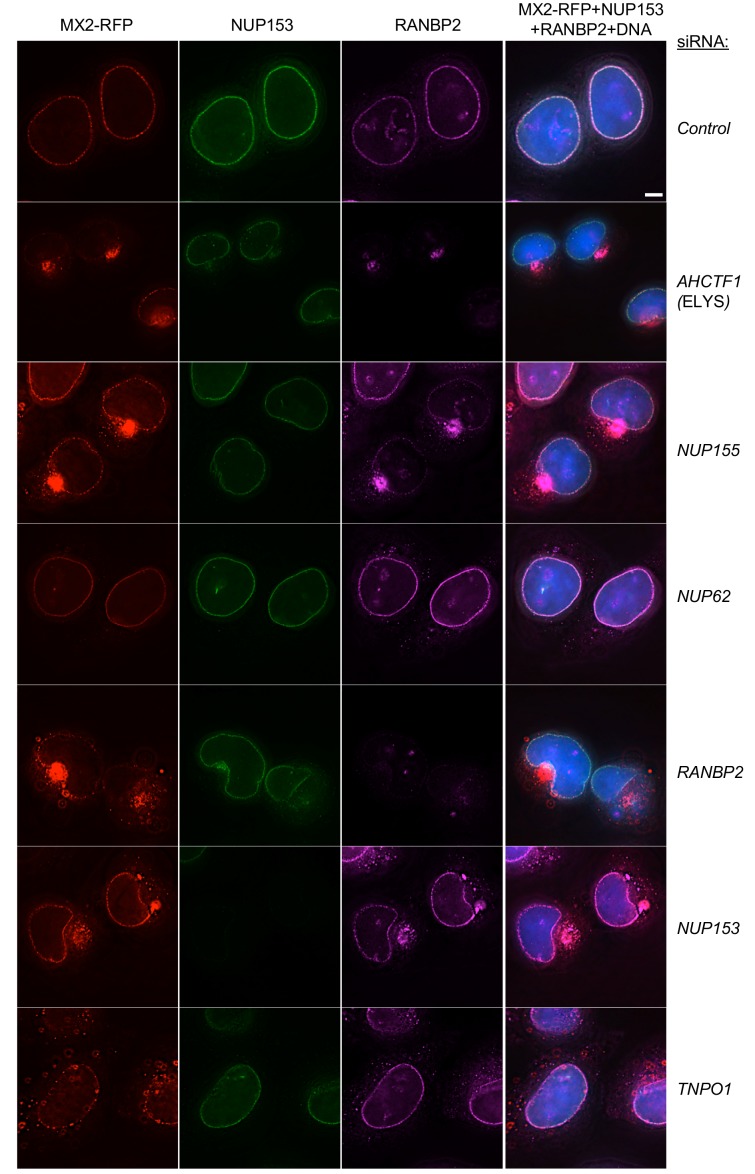
Effects of depleting individual Nups on NPC integrity and function, and MX2 localization. Deconvolution microscopic images (single optical sections) of HeLa cells expressing MX2-RFP (red, stably transduced with a doxycycline inducible vector), immunoflourescently stained NUP153 (green), RANBP2 (purple), and Hoechst-stained DNA. Cells were fixed and stained 64 hr after transfection with the indicated siRNA. Optical sections are approximately through the center of the vertical dimension on the nucleus. Scale bar = 5 μm. Representative of two independent experiments, with at least three images acquired per experiment.

We also determined whether the recruitment of several representative Nups to the NPC was altered upon knockdown of each individual Nup. Specifically, the localizations of NUP153, RANBP2, NUP98, NUP62 and NUP214 were assessed following siRNA knockdown of all Nups in HeLa and/or HT1080 cells (Figure 6 and Figure 6—figure supplements 1–14). These Nups were chosen for assessment as representatives of the majority of the NPC subcomplexes, and based on the availability of antibodies that worked well in immunofluorescence assays.

**Figure 6—figure supplement 1.**
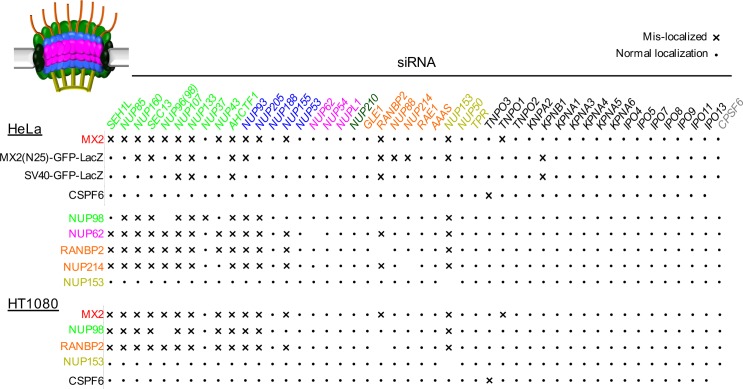
Effects of depleting individual Nups on NPC integrity and function, and MX2 localization. Summary of the localization of Nups, MX2-RFP, CPSF6-RFP, or NLS-GFP-LacZ fusions upon siRNA transfection of HeLa or HT1080 cells shown in Figure 6, and Figure 6—figure supplements 1–18. Aberrant localization following siRNA transfection in ≥~80% of cells is indicated by an ‘x’ and normal localization is indicated by a dot.

**Figure 6—figure supplement 2.**
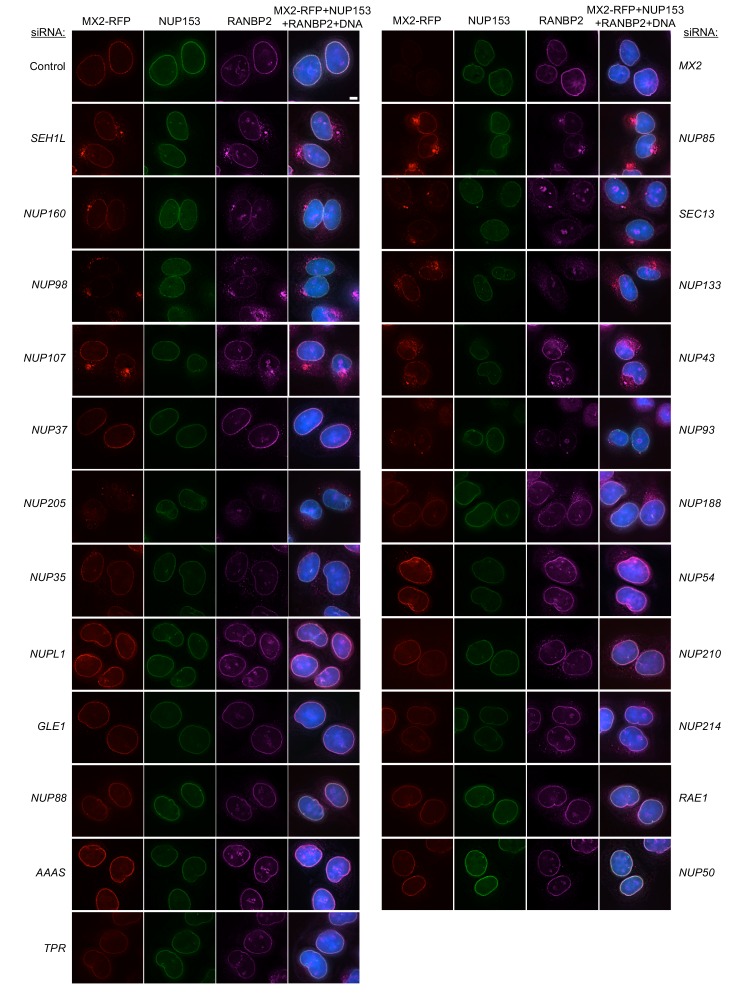
Localization of MX2, NUP153, and RANBP2 in HeLa cells following transfection with Nup/NTR targeting siRNA. Deconvolution microscopic images (single optical sections) of HeLa cells expressing MX2-RFP (red, stably transduced with a doxycycline inducible vector), immunoflourescently stained NUP153 (green), RANBP2 (purple), and Hoescht-stained DNA. Cells were fixed and stained 64 hr after transfection with the indicated siRNA. Optical sections are approximately through the center of the vertical dimension on the nucleus. Scale bar = 5 μm. Representative of two independent experiments, with at least three images acquired per experiment.

**Figure 6—figure supplement 3.**
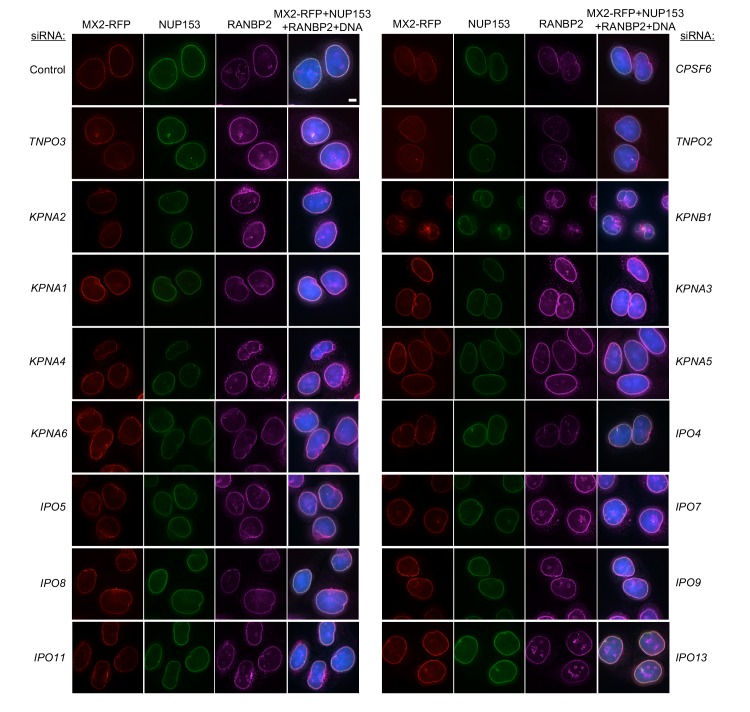
Localization of MX2, NUP153, and RANBP2 in HeLa cells following transfection with Nup/NTR targeting siRNA continued. Deconvolution microscopic images (single optical sections) of HeLa cells expressing MX2-RFP (red, stably transduced with a doxycycline inducible vector), immunoflourescently stained NUP153 (green), RANBP2 (purple), and Hoescht-stained DNA. Cells were fixed and stained 64 hr after transfection with the indicated siRNA. Optical sections are approximately through the center of the vertical dimension on the nucleus. Scale bar = 5 μm. Representative of two independent experiments, with at least three images acquired per experiment.

**Figure 6—figure supplement 4.**
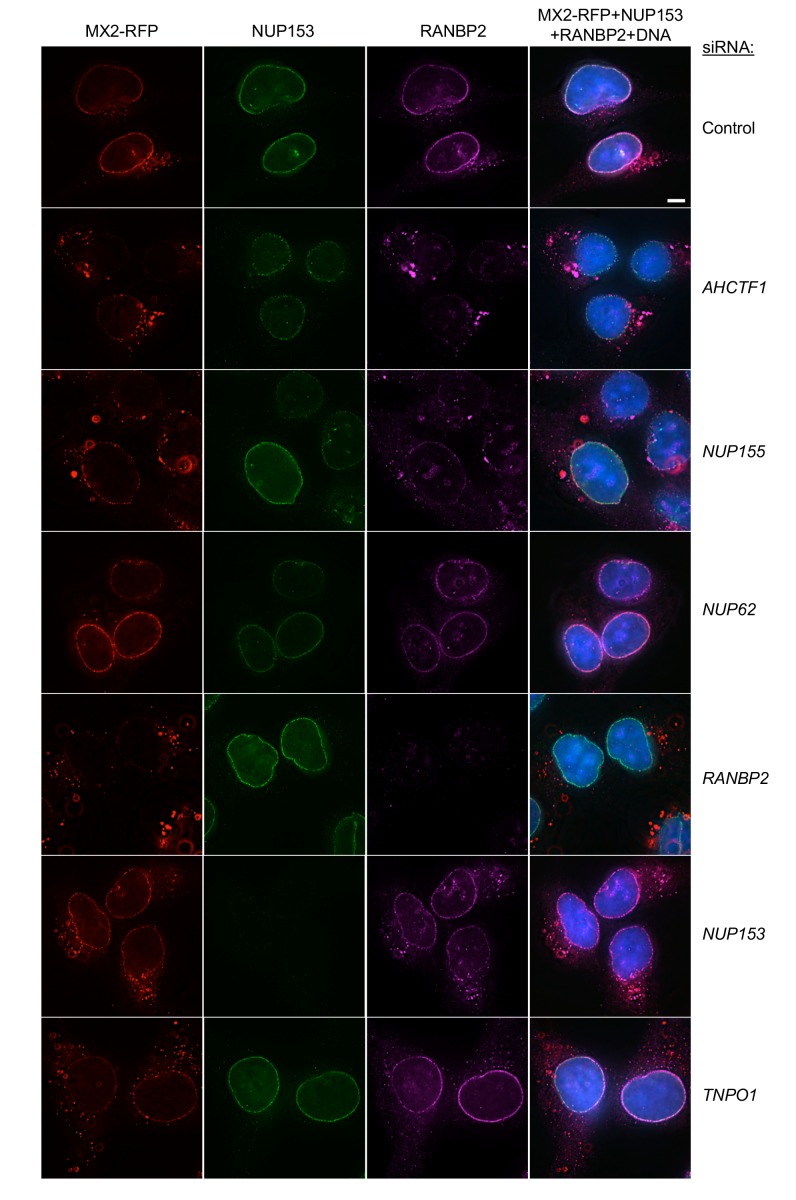
Localization of MX2, NUP153, and RANBP2 in HT1080 cells following transfection with Nup/NTR targeting siRNA. Deconvolution microscopic images (single optical sections) of HT1080 cells expressing MX2-RFP (red, stably transduced with a doxycycline inducible vector), immunoflourescently stained NUP153 (green), RANBP2 (purple), and Hoescht-stained DNA. Cells were fixed and stained 64 hr after transfection with the indicated siRNA. Optical sections are approximately through the center of the vertical dimension on the nucleus. Scale bar = 5 μm. Representative of two independent experiments, with at least three images acquired per experiment.

**Figure 6—figure supplement 5.**
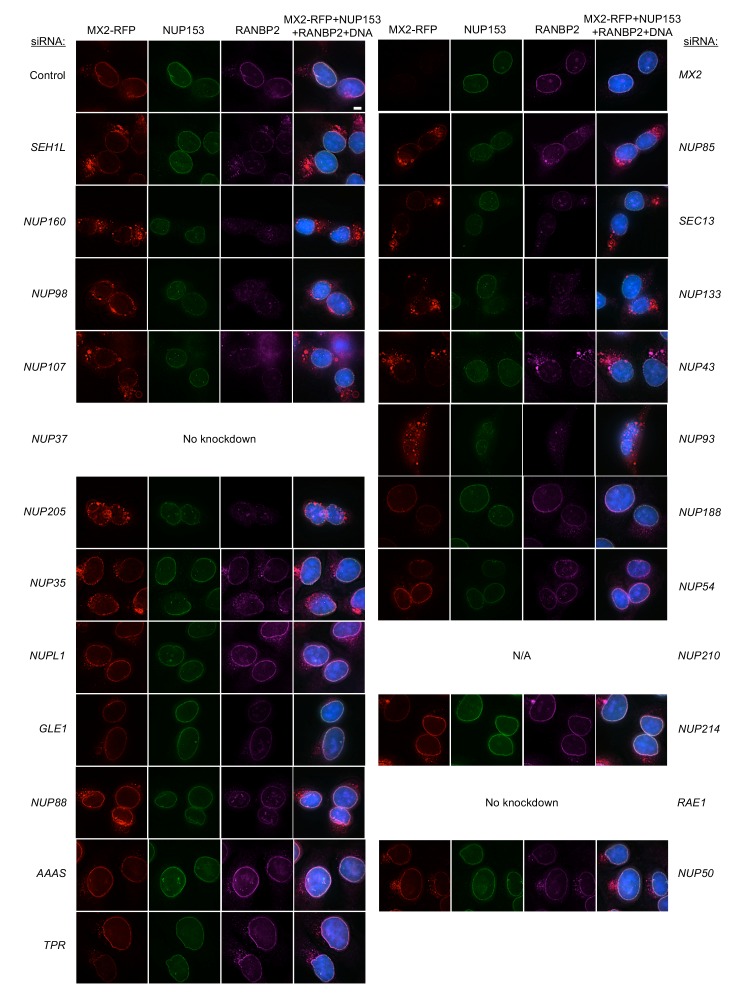
Localization of MX2, NUP153, and RANBP2 in HT1080 cells following transfection with Nup/NTR targeting siRNA continued. Deconvolution microscopic images (single optical sections) of HT1080 cells expressing MX2-RFP (red, stably transduced with a doxycycline inducible vector), immunoflourescently stained NUP153 (green), RANBP2 (purple), and Hoescht-stained DNA. Cells were fixed and stained 64 hr after transfection with the indicated siRNA. Optical sections are approximately through the center of the vertical dimension on the nucleus. Scale bar = 5 μm. Representative of two independent experiments, with at least three images acquired per experiment.

**Figure 6—figure supplement 6.**
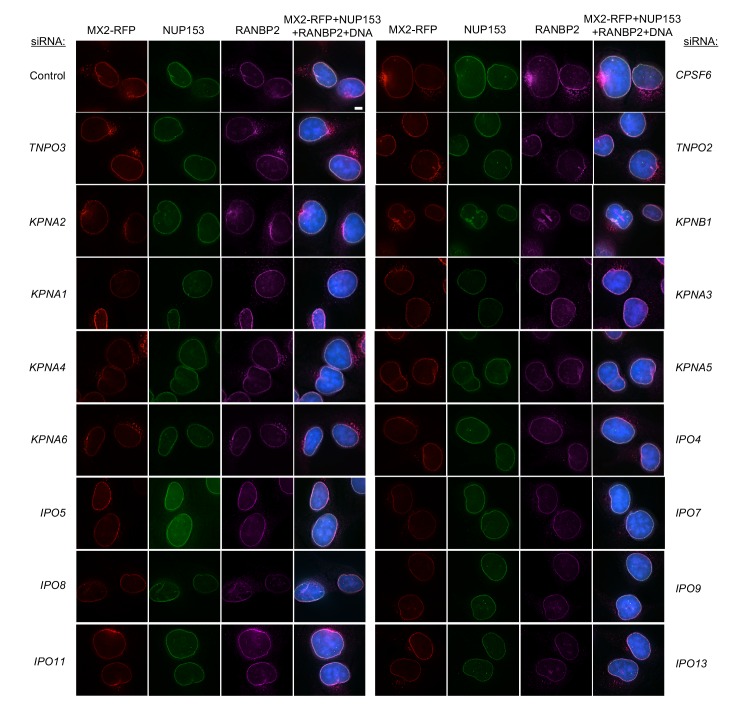
Localization of MX2, NUP153, and RANBP2 in HT1080 cells following transfection with Nup/NTR targeting siRNA continued. Deconvolution microscopic images (single optical sections) of HT1080 cells expressing MX2-RFP (red, stably transduced with a doxycycline inducible vector), immunoflourescently stained NUP153 (green), RANBP2 (purple), and Hoescht-stained DNA. Cells were fixed and stained 64 hr after transfection with the indicated siRNA. Optical sections are approximately through the center of the vertical dimension on the nucleus. Scale bar = 5 μm. Representative of two independent experiments, with at least three images acquired per experiment.

**Figure 6—figure supplement 7.**
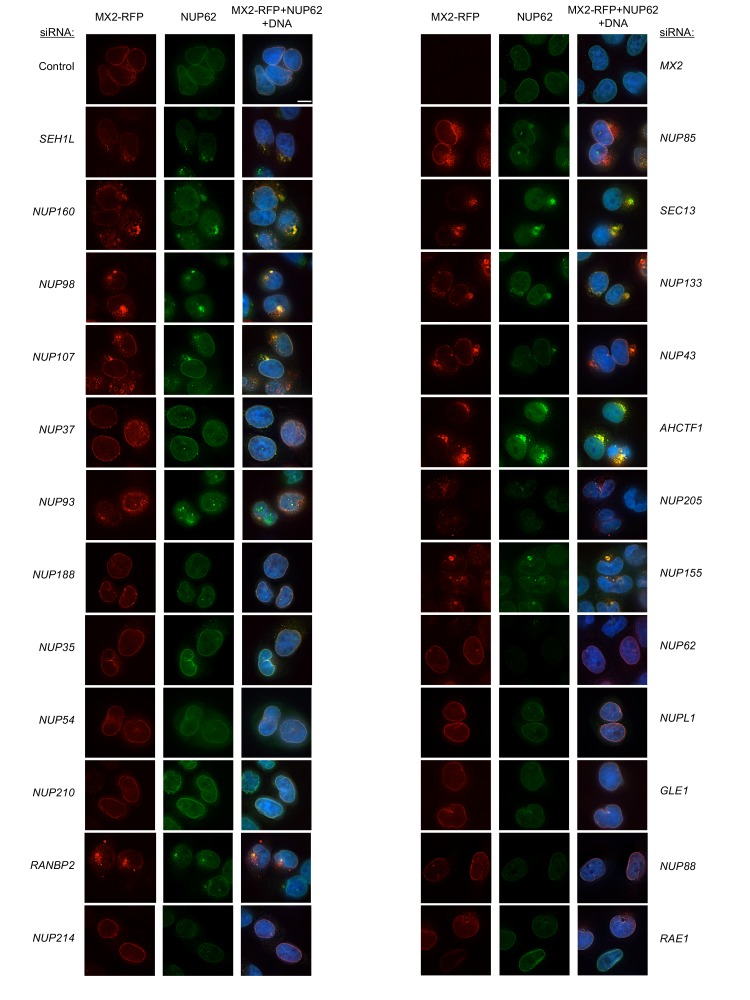
Localization of MX2 and NUP62 following transfection with Nup/NTR targeting siRNA. Deconvolution microscopic images (single optical sections) of HeLa cells expressing MX2-RFP (red, stably transduced with a doxycycline inducible vector), immunoflourescently stained NUP62 (green), and Hoechst-stained DNA. Cells were fixed and stained 64 hr after transfection with the indicated siRNA. Optical sections are approximately through the center of the vertical dimension on the nucleus. Scale bar = 10 μm. Representative of two independent experiments, with at least three images acquired per experiment.

**Figure 6—figure supplement 8.**
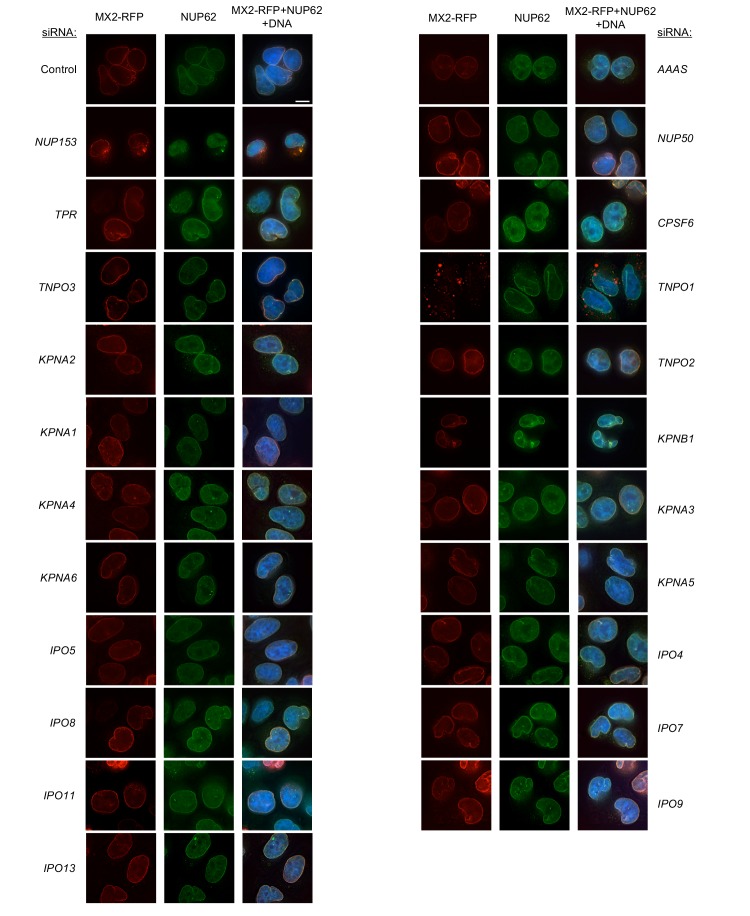
Localization of MX2 and NUP62 following transfection with Nup/NTR targeting siRNA continued. Deconvolution microscopic images (single optical sections) of HeLa cells expressing MX2-RFP (red, stably transduced with a doxycycline inducible vector), immunoflourescently stained NUP62 (green), and Hoechst-stained DNA. Cells were fixed and stained 64 hr after transfection with the indicated siRNA. Optical sections are approximately through the center of the vertical dimension on the nucleus. Scale bar = 10 μm. Representative of two independent experiments, with at least three images acquired per experiment.

**Figure 6—figure supplement 9.**
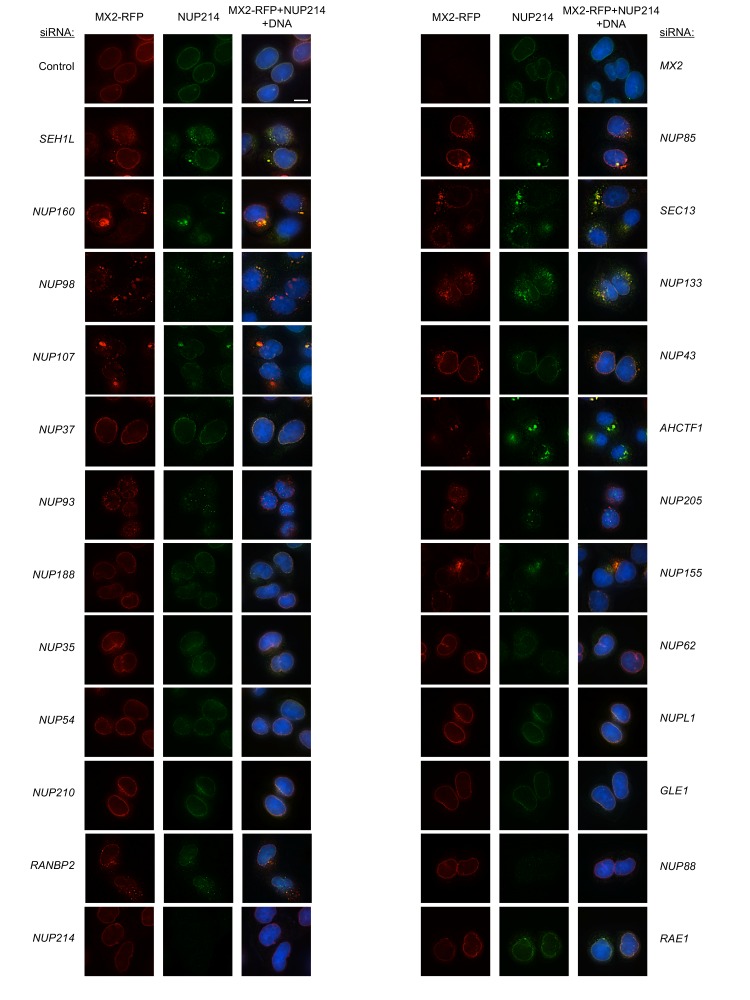
Localization of MX2 and NUP214 following transfection with Nup/NTR targeting siRNA. Deconvolution microscopic images (single optical sections) of HeLa cells expressing MX2-RFP (red, stably transduced with a doxycycline inducible vector), immunoflourescently stained NUP214 (green), and Hoescht-stained DNA. Cells were fixed and stained 64 hr after transfection with the indicated siRNA. Optical sections are approximately through the center of the vertical dimension on the nucleus. Scale bar = 10 μm. Representative of two independent experiments, with at least three images acquired per experiment.

**Figure 6—figure supplement 10.**
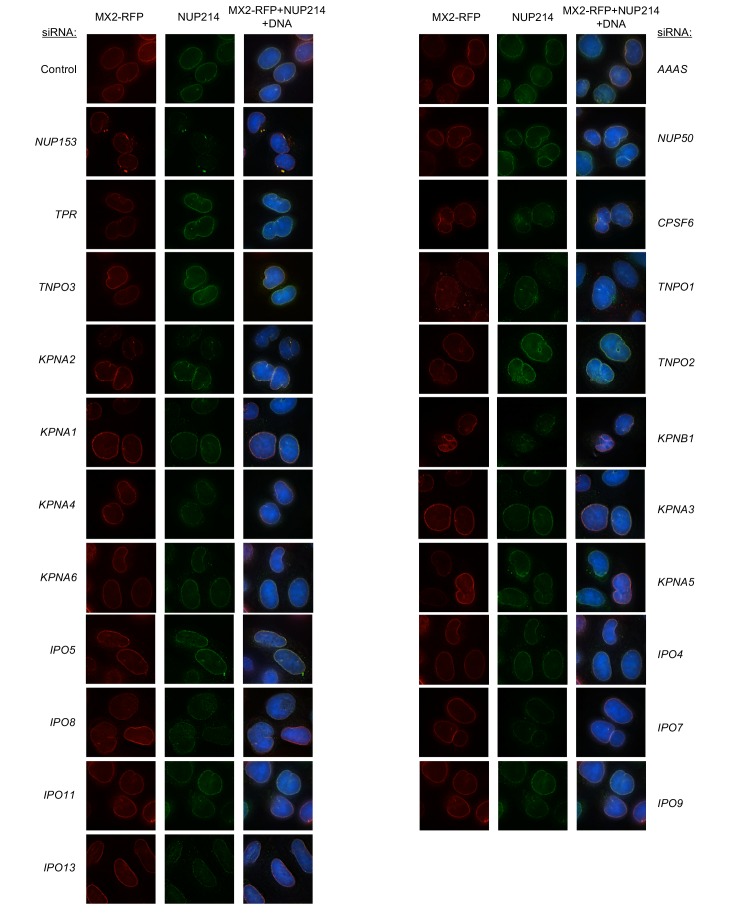
Localization of MX2 and NUP214 following transfection with Nup/NTR targeting siRNA continued. Deconvolution microscopic images (single optical sections) of HeLa cells expressing MX2-RFP (red, stably transduced with a doxycycline inducible vector), immunoflourescently stained NUP214 (green), and Hoescht-stained DNA. Cells were fixed and stained 64 hr after transfection with the indicated siRNA. Optical sections are approximately through the center of the vertical dimension on the nucleus. Representative of two independent experiments, with at least three images acquired per experiment.

**Figure 6—figure supplement 11.**
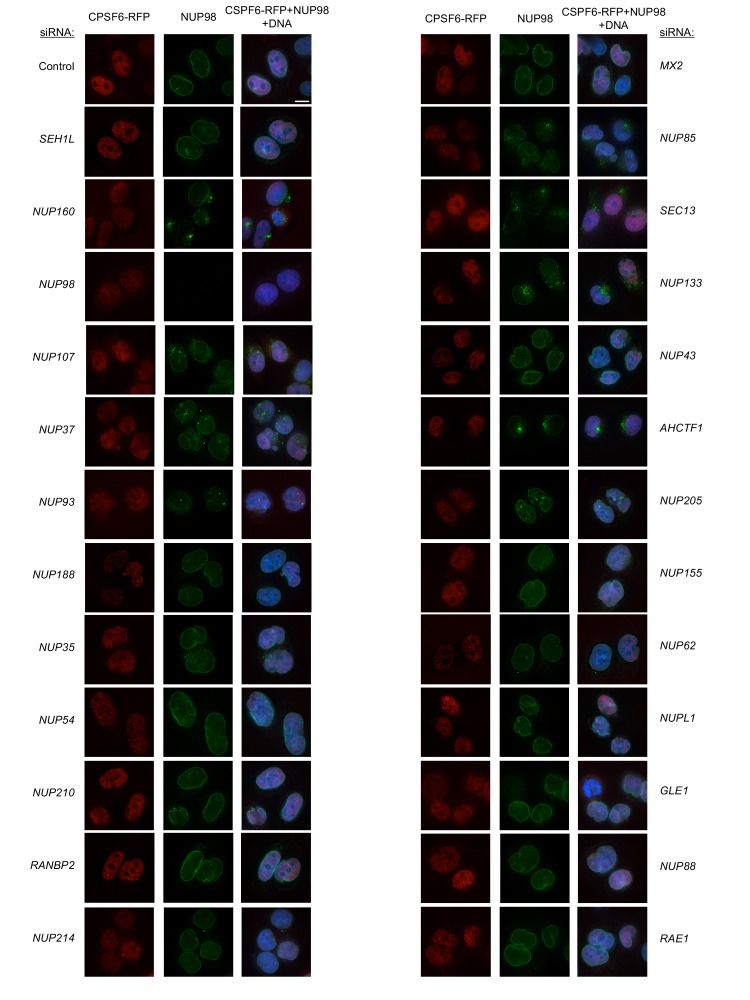
Localization of CPSF6 and NUP98 in HeLa cells following transfection with Nup/NTR targeting siRNA. Deconvolution microscopic images (single optical sections) of HeLa cells expressing CPSF6-RFP (red, stably transduced with a doxycycline inducible vector), immunoflourescently stained NUP98 (green), and Hoescht-stained DNA. Cells were fixed and stained 64 hr after transfection with the indicated siRNA. Optical sections are approximately through the center of the vertical dimension on the nucleus. Scale bar = 10 μm. Representative of two independent experiments, with at least three images acquired per experiment.

**Figure 6—figure supplement 12.**
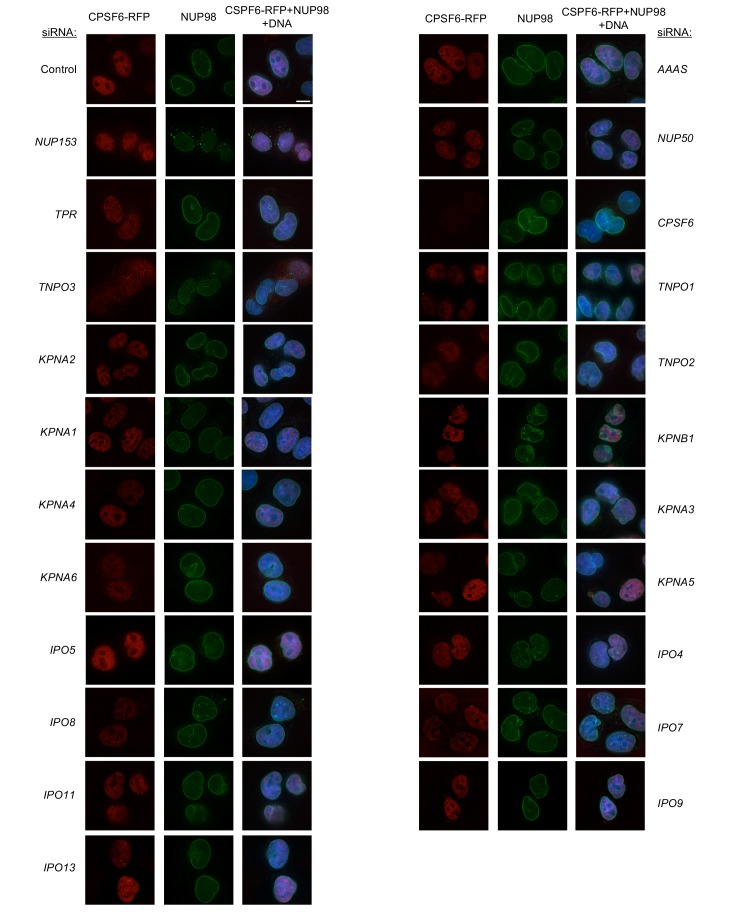
Localization of CPSF6 and NUP98 in HeLa cells following transfection with Nup/NTR targeting siRNA continued. Deconvolution microscopic images (single optical sections) of HeLa cells expressing CPSF6-RFP (red, stably transduced with a doxycycline inducible vector), immunoflourescently stained NUP98 (green), and Hoescht-stained DNA. Cells were fixed and stained 64 hr after transfection with the indicated siRNA. Optical sections are approximately through the center of the vertical dimension on the nucleus. Scale bar = 10 μm. Representative of two independent experiments, with at least three images acquired per experiment.

**Figure 6—figure supplement 13.**
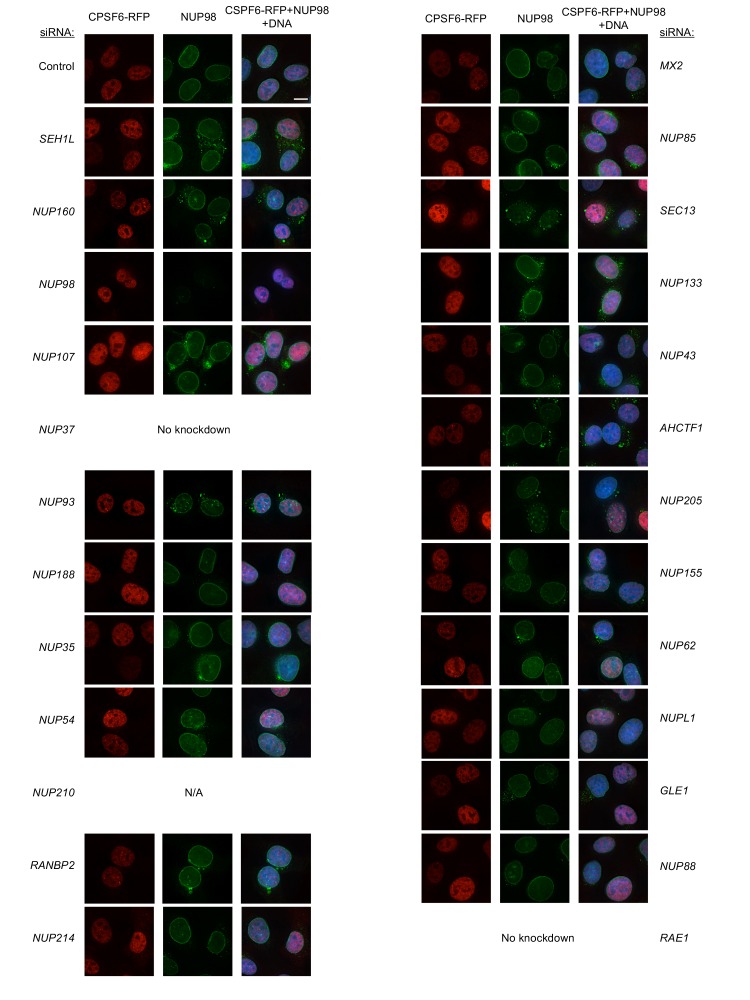
Localization of CPSF6 and NUP98 in HT1080 cells following transfection with Nup/NTR targeting siRNA. Deconvolution microscopic images (single optical sections) of HT1080 cells expressing CPSF6-RFP (red, stably transduced with a doxycycline inducible vector), immunoflourescently stained NUP98 (green), and Hoescht-stained DNA. Cells were fixed and stained 64 hr after transfection with the indicated siRNA. Optical sections are approximately through the center of the vertical dimension on the nucleus. Scale bar = 10 μm. Representative of two independent experiments, with at least three images acquired per experiment.

**Figure 6—figure supplement 14.**
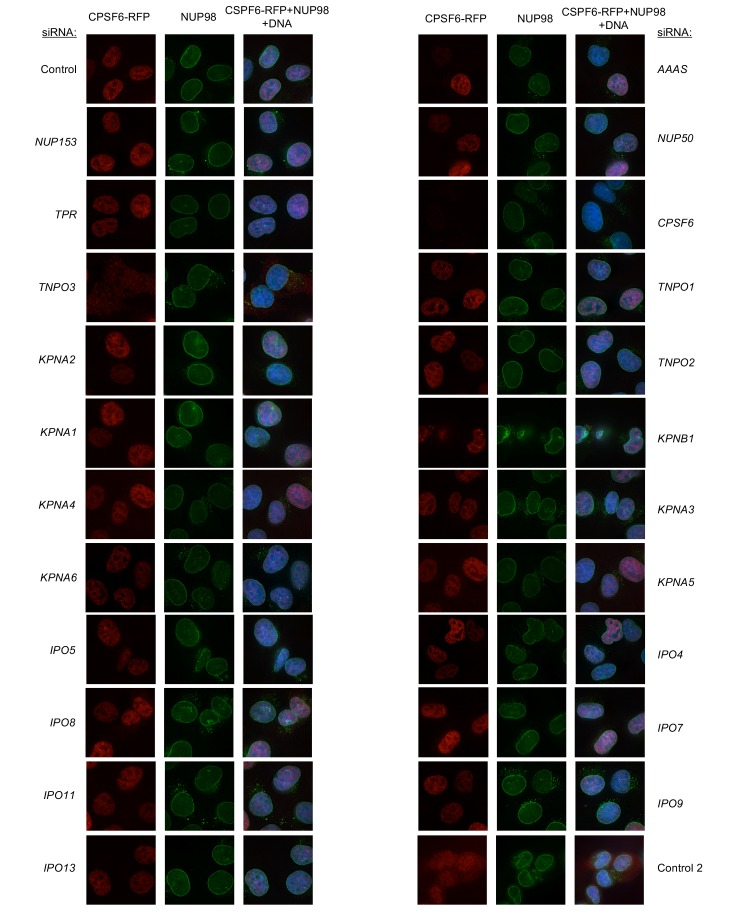
Localization of CPSF6 and NUP98 in HT1080 cells following transfection with Nup/NTR targeting siRNA continued. Deconvolution microscopic images (single optical sections) of HT1080 cells expressing CPSF6-RFP (red, stably transduced with a doxycycline inducible vector), immunoflourescently stained NUP98 (green), and Hoescht-stained DNA. Cells were fixed and stained 64 hr after transfection with the indicated siRNA. Optical sections are approximately through the center of the vertical dimension on the nucleus. Representative of two independent experiments, with at least three images acquired per experiment.

**Figure 6—figure supplement 15.**
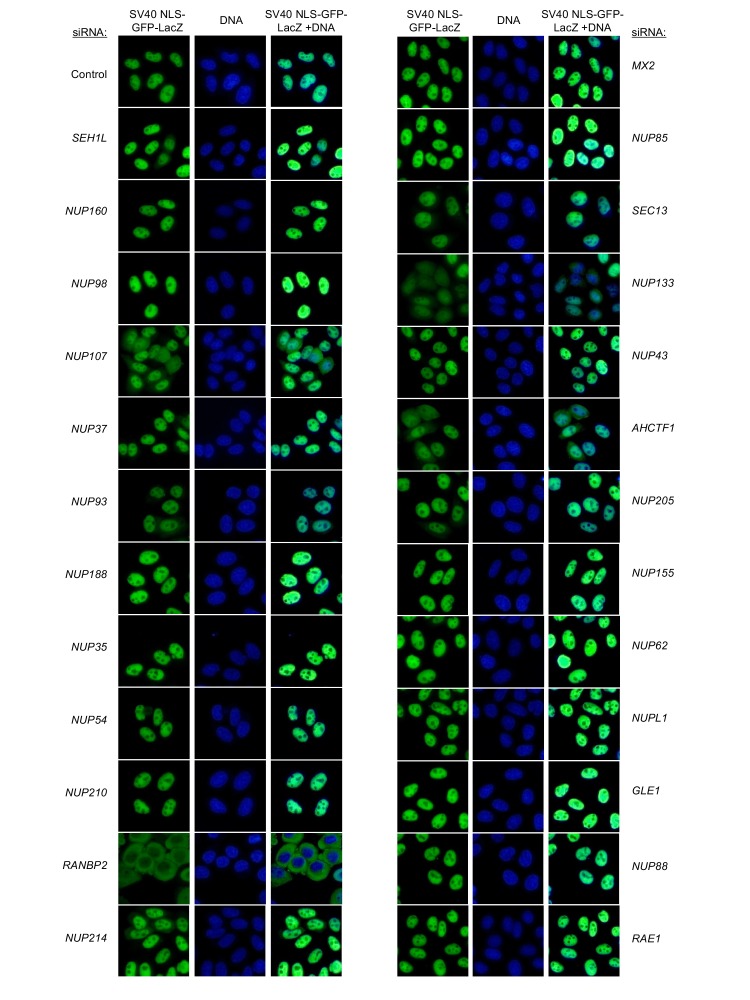
Localization of SV40 NLS-GFP-LacZ following transfection with Nup/NTR targeting siRNA. HeLa cells stably transduced with SV40 NLS-GFP-LacZ fusion protein (green) and doxycycline-inducible MX2-RFP in the absence of doxycycline fixed and stained with Hoescht 64 hr after transfection with the indicated siRNA. Representative of two independent experiments, with at least three images acquired per experiment.

**Figure 6—figure supplement 16.**
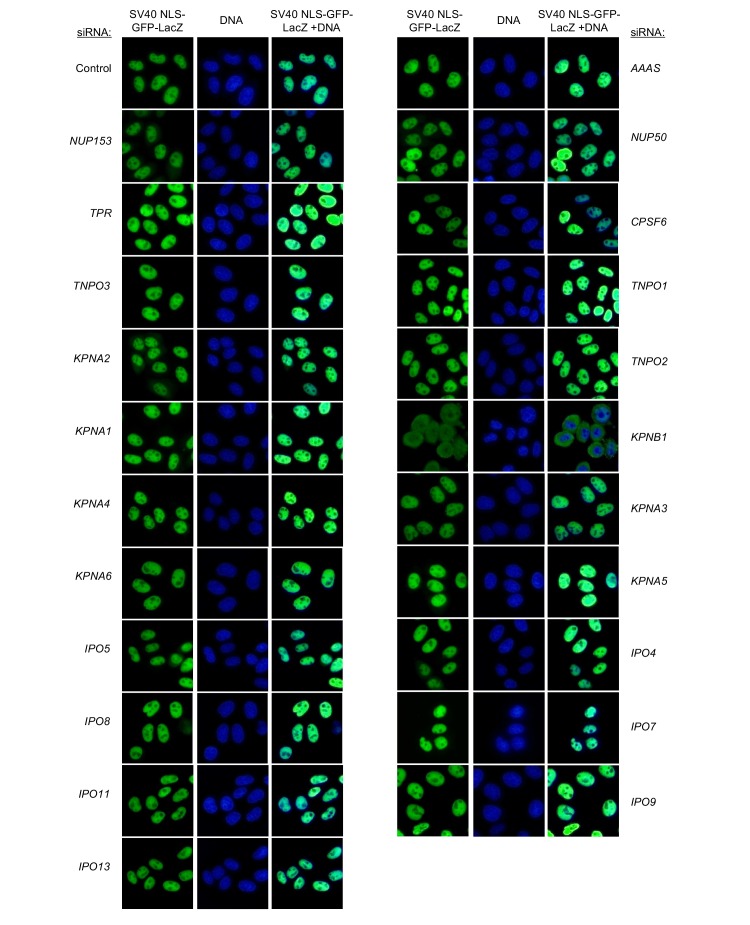
Localization of SV40 NLS-GFP-LacZ following transfection with Nup/NTR targeting siRNA continued. HeLa cells stably transduced with SV40 NLS-GFP-LacZ fusion protein (green) and doxycycline-inducible MX2-RFP in the absence of doxycycline fixed and stained with Hoescht 64 hr after transfection with the indicated siRNA. Representative of two independent experiments, with at least three images acquired per experiment.

**Figure 6—figure supplement 17.**
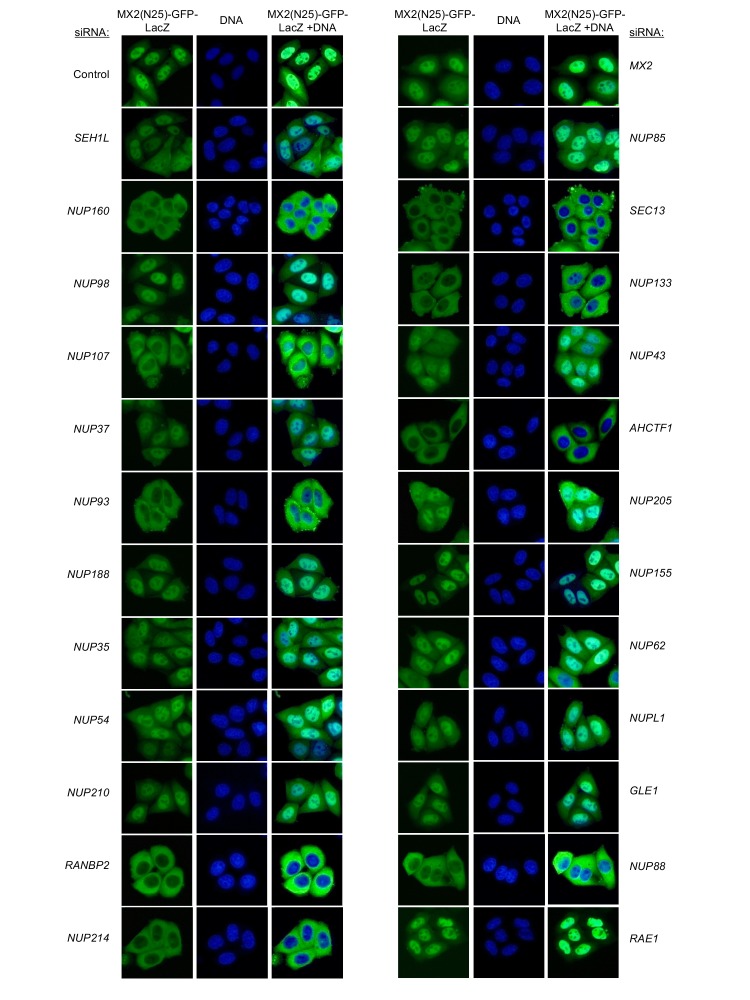
Localization of MX2(N25)-GFP-LacZ following transfection with Nup/NTR targeting siRNA. HeLa cells stably transduced with MX2(N25)-GFP-LacZ fusion protein (green) and doxycycline-inducible MX2-RFP in the absence of doxycycline fixed and stained with Hoescht 64 hr after transfection with the indicated siRNA. Representative of two independent experiments, with at least three images acquired per experiment.

**Figure 6—figure supplement 18.**
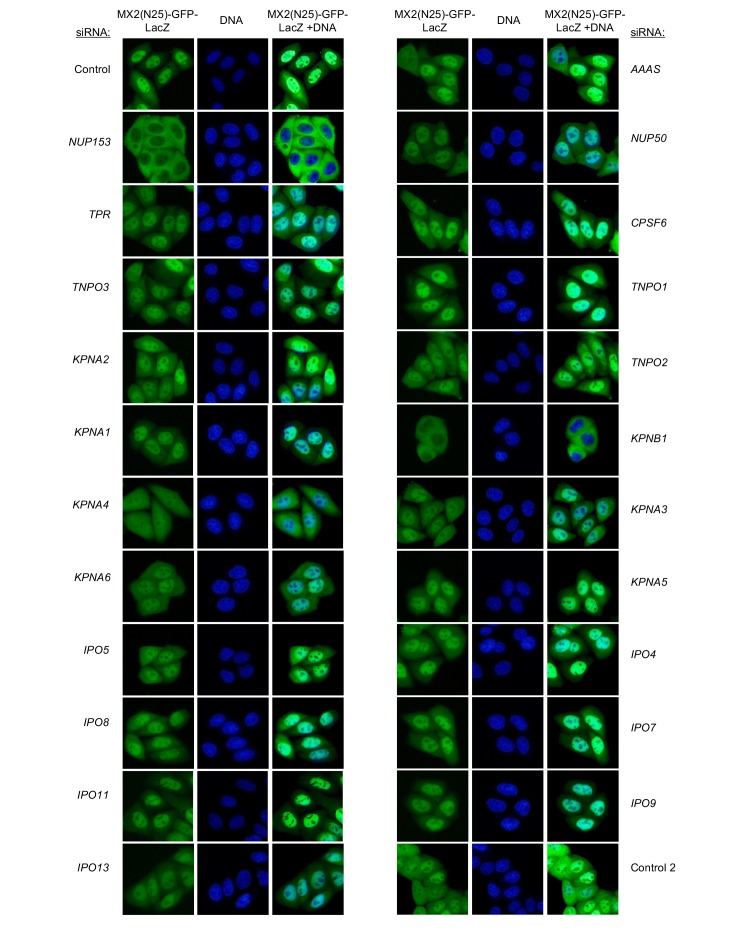
Localization of MX2(N25)-GFP-LacZ following transfection with Nup/NTR targeting siRNA continued. HeLa cells stably transduced with MX2(N25)-GFP-LacZ fusion protein (green) and doxycycline-inducible MX2-RFP in the absence of doxycycline fixed and stained with Hoescht 64 hr after transfection with the indicated siRNA. Representative of two independent experiments, with at least three images acquired per experiment.

**Figure 6—figure supplement 19.**
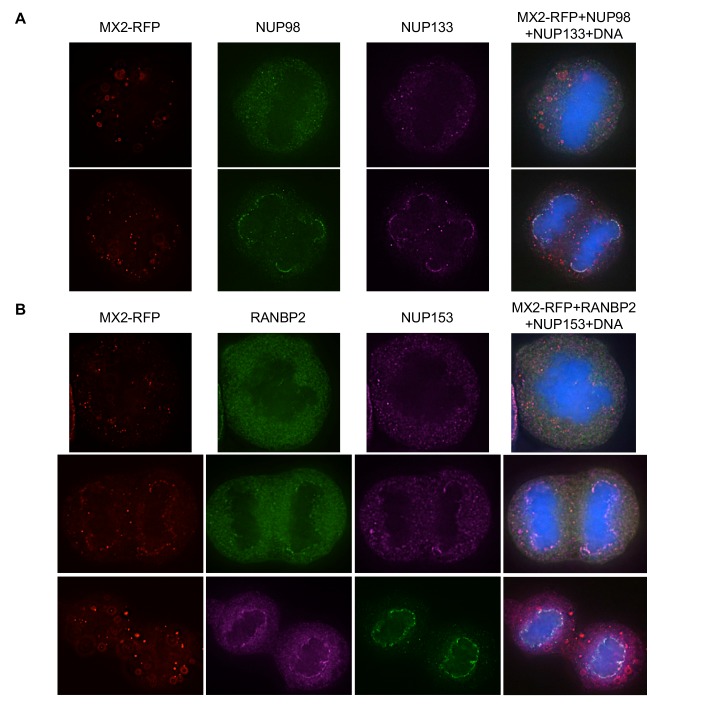
Localization of MX2 and Nups in mitotic HeLa cells. Deconvolution microscopic images (single optical sections) of HeLa cells expressing MX2-RFP (red, stably transduced with a doxycycline inducible vector). Representative of three independent experiments, with at least three images acquired per experiment. (**A**) Immunoflourescently stained NUP98 (green), NUP133 (purple), and Hoescht-stained DNA. Top- early- mitotic cell with condensed chromatin; bottom- late-mitotic cell with separating chromosomes. (**B**) Immunoflourescently stained RANBP2 (top, middle-green; bottom-purple), NUP153 (top, middle-purple; bottom-green), and Hoescht-stained DNA. Top- early- mitotic cell with condensed chromatin; middle and bottom- late-mitotic cells with separating chromosomes.

**Figure 6—figure supplement 20.**
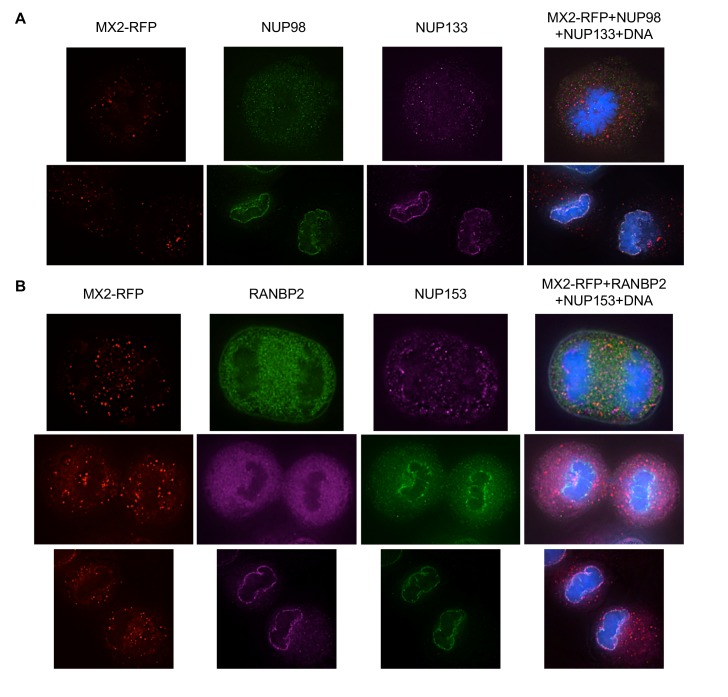
Localization of MX2 and Nups in mitotic HT1080 cells. Deconvolution microscopic images (single optical sections) of HT1080 cells expressing MX2-RFP (red, stably transduced with a doxycycline inducible vector). Representative of three independent experiments, with at least three images acquired per experiment. (**A**) Immunoflourescently stained NUP98 (green), NUP133 (purple), and Hoescht-stained DNA. Top- early- mitotic cell with condensed chromatin; bottom- late-mitotic cell with separating chromosomes. (**B**) Immunoflourescently stained RANBP2 (top-green; middle, bottom-purple), NUP153 (top-purple; middle, bottom-green), and Hoescht-stained DNA. Cells in anaphase through telophase/cytokinesis from top to bottom.

The effects of Nup knockdown on the localization of other Nups were diverse. Strikingly, NUP153 remained localized at the nuclear envelope following knockdown of each of the other Nups. This finding is likely a reflection of the fact that NUP153 recruitment to the nuclear envelope is an early event during nuclear pore formation, and its incorporation into the nuclear envelope is not therefore expected to be dependent on other Nups (Figure 6 and Figure 6—figure supplements 1–6) (Bodoor et al., 1999; Vollmer et al., 2015). In contrast, several Nup siRNA knockdowns caused mislocalization of RANBP2, NUP214, NUP62, and/or NUP98 (Figure 6, Figure 6—figure supplements 1–14). Depletion of the Nups known to participate in the early stages of NPC formation (e.g. ELYS and NUP153), or those known to form the structural scaffold of the nuclear pore complex (e.g. NUP107 and NUP133), or those with the clear effects on cell viability (e.g. NUP93 and NUP205), had the most obvious and pervasive effects on the localization of other Nups. Conversely, the knockdown of several Nups (e.g. NUP188, NUP214, and NUP88) had no effect on the apparent localization of the other Nups, despite having clear effects on their protein levels. This finding suggests that some Nups are destabilized in the absence of their NPC binding partners. Importantly, the aforementioned results highlight the pleiotropic effects associated with the depletion of some individual Nups (e.g. NUP153) and indicate that cautious interpretation of targeted Nup knockdown or knockout experiments in general is warranted. In general, the knockdown of an individual Nup often changed the level of other Nups and the composition of NPCs in ways that were not easily predicted.

### Effects of depleting individual Nups on NLS function

To assess nuclear pore function under conditions of Nup knockdown, we generated cell lines expressing GFP-LacZ fusion proteins appended with one of two different NLS sequences. As expected, the unmodified GFP-LacZ fusion was excluded from the nucleus, while a GFP-LacZ appended with a canonical SV40 large T-antigen NLS appeared almost entirely localized to the nucleus (Figure 6—figure supplements 15 and 16). When appended to GFP-LacZ, the N-terminal 25 amino acids of MX2 acted as an NLS, and caused MX2(25)NLS-GFP-LacZ to localize to the interior of the nucleus (Figure 6—figure supplements 17 and 18). Interestingly, the nuclear interior localization of MX2(25)NLS-GFP-LacZ contrasted with the nuclear pore localization of MX2 itself, and with the nuclear pore localization of MX2(25)-Mx1 (Goujon et al., 2014), suggesting that some property of MX2 and MX1 confers nuclear pore entrapment on a signal that would direct transport of an otherwise inert cargo to the nuclear interior.

Depletion of Nups and NTRs revealed that most perturbations allowed residual nuclear transport to proceed. Indeed, the nuclear accumulation of the SV40 NLS-GFP-LacZ was unaffected or only marginally affected by most Nup or NTR depletions. The exceptions to this were NUP107, NUP133, and ELYS/ACHTF1 whose depletion caused some reduction in nuclear accumulation, while RANBP2 and KPNB1 depletion caused nearly complete nuclear exclusion of SV40 NLS-GFP-LacZ. In the case of MX2(25)NLS-GFP-LacZ, nuclear accumulation was robust, but not as complete as was the case for SV40 NLS-GFP-LacZ. Moreover, a larger number of Nup depletions cause partial or apparently complete inhibition of MX2(25)NLS-GFP-LacZ nuclear accumulation. For example, NUP214, NUP88, or NUP153 depletion caused near complete exclusion of MX2(25)NLS-GFP-LacZ from the nucleus, but did not affect nuclear accumulation of SV40 NLS-GFP-LacZ (Figure 6—figure supplements 1 and 5–8).

These experiments indicate that at least some nuclear pores survive and retain transport activity following the knockdown of most individual Nups. Moreover, nuclear transport driven by two different NLS sequences exhibited marked differences in sensitivity to perturbation by siRNA-mediated alterations of Nup or NTR levels.

### Nup composition or conformation controls NPC recruitment of MX2

Because individual Nup depletions could affect recruitment of other Nups as well as NPC function, we next determined whether the Nup depletions affected recruitment of MX2 to the NPC. To accomplish this, we used a doxycycline-inducible C-terminally tagged MX2-tagRFP expression construct. Additionally, because perturbation of nuclear transport has been shown to cause relocalization of CPSF6 and reveal a mis-localization dependent antiviral activity (De Iaco et al., 2013; Fricke et al., 2013; Hori et al., 2013; Lee et al., 2010; Price et al., 2012), we also monitored the localization of CPSF6-RFP in the same way. In control siRNA treated cells, the MX2-RFP fusion was concentrated at the nuclear envelope (Figure 6), and the CPSF6-RFP fusion was found only within the nuclear interior in most cells (Figure 6—figure supplements 11–14). However, cytoplasmic localization of CPSF6 was observed in some (~5%) control siRNA treated cells (Figure 6—figure supplement 14), indicating that CPSF6 may not be confined to the nucleus at all times. Moreover, MX2 sometimes appeared in small aggregates in the cytoplasm of unmanipulated HT1080 cells, in addition to localizing at the nuclear envelope (Figure 6—figure supplement 4).

Several Nup knockdowns (SEH1, NUP85, NUP160, SEC13, NUP98, NUP107, NUP133, NUP43, ELYS, NUP93, NUP205, NUP155, RANBP2, and NUP153), and one NTR knockdown (TNPO1) resulted in a dramatic mislocalization of MX2 (Figure 6 and Figure 6—figure supplements 1–10). There were no evident differences between HeLa and HT1080 cells in the Nup requirements for MX2 localization to nuclear pores. Consistent with the finding that Nup knockdowns did not induce a generalized global block to nuclear transport (see above), CPSF6 remained localized within the nucleus following all Nup/NTR depletions, with the exception of TNPO3 [TNPO3 is known to be required for nuclear localization of CPSF6 (Figure 6—figure supplements 1 and 11–14) (De Iaco et al., 2013; Fricke et al., 2013).

Interestingly, MX2 exhibited similar Nup requirements for recruitment to the NPC as did RANBP2 and NUP62 (Figure 6—figure supplement 1). Additionally, MX2 required RANBP2 (but not NUP62) for NPC recruitment. However, since we were not able to observe the subcellular localization of all Nups in these experiments, it is also possible that additional Nups have the same requirements for recruitment to the NPC as do RANBP2, NUP62, and MX2.

Because the recruitment of MX2 to NPCs was clearly dependent on certain Nups, we also examined the localization of MX2 in mitotic cells. In mitotic cells lacking a discernable nuclear envelope, the MX2-RFP fusion appeared as aggregates dispersed throughout the cell (Figure 6—figure supplements 19 and 20). Observation of cells in the later stages of mitosis, in which the nuclear envelope reforms and NPC assembly occurs, showed that MX2 remained dispersed throughout the cell, and was only visible at the nuclear envelope in cells exhibiting the terminal stages of NPC formation (Figure 6—figure supplements 19 and 20). In particular, MX2 remained dispersed in the cytosol when NUP153, NUP133, and NUP98 had been recruited to the nuclear envelope, which occurs during anaphase (reviewed in (Burke and Ellenberg, 2002; Schellhaus et al., 2016). RANBP2 recruitment to the NPC is a late event and follows the establishment of the structural pore and the central channel (reviewed in (Burke and Ellenberg, 2002; Schellhaus et al., 2016) and MX2 was only visualized at the nuclear envelope subsequent to the recruitment of RANBP2. These findings suggest that MX2 recruitment is specifically dependent of the presence of a Nup (possibly RANBP2) that is incorporated late in the assembly of the NPC. Alternatively, MX2 might interact with the NPC only as an assembled structure, rather than with a single Nup component.

**Figure 7.**
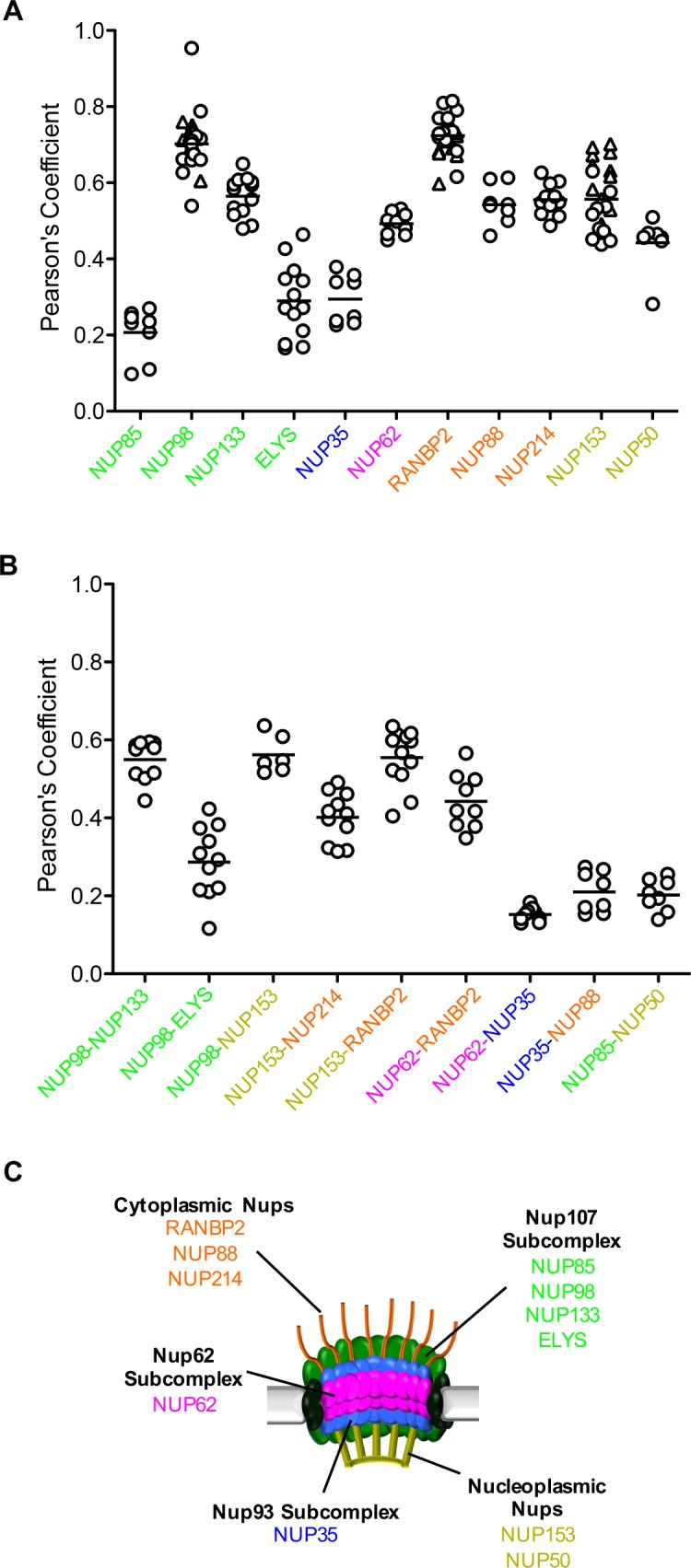
Localization of MX2 and Nups. (**A**) Pearson’s coefficient for co-localization of MX2 with indicated Nups and (B) Nups with one another of deconvolution microscopic images of HeLa cells expressing MX2-RFP and immunoflourescently stained with Nups (color coded as in Figure 4A). Each data point represents an individual cell and the horizontal bar is the mean (n ≥ 6). For NUP98, RANBP2, and NUP153, triangles and circles in A) represent cells stained with secondary antibodies coupled to different flourophores (AlexaFlour-488 and AlexaFlour-647).

Although we were not able to resolve individual nuclear pores via immunofluorescence, we could observe differences in the co-localization of MX2 with a number of Nups in interphase cells (Figure 7A and Figure 7—figure supplements 1–3). In particular, the MX2-RFP fusion was most strongly co-localized with NUP98, NUP133, NUP62, RANBP2, NUP88, NUP214, NUP153, and NUP50, but showed significantly less co-localization with NUP85, ELYS, and NUP35. These differences were also reflected in the co-localization of Nups with one another (Figure 7B and Figure 7—figure supplements 1–3). For example, NUP98, NUP133, NUP153, NUP214, RANBP2, and NUP62 colocalized well with each other and less well with ELYS, NUP35, and NUP85. These findings could be explained by varied composition of individual nuclear pores, and/or new pores assembling during interphase, in which some, but not all Nups are present within a nascent NPC. The details of interphase NPC assembly are not fully understood, but a number of important features distinguish interphase and mitotic NPC assembly (see discussion). It is therefore conceivable that nuclear pores at various stages of formation could be observed within a single cell, and possible that pores formed during interphase could be compositionally or conformationally distinct from those formed post-mitosis. It is also possible that the apparent heterogeneity in NPC observed in immunofluorescence reflects the occlusion of Nup antibody epitopes in a subset of NPCs. Epitopes could be inaccessible to antibodies as a result of conformational variability (such as during transport or pore formation) and may therefore not actually be reflective of compositional differences. In either case, these results suggest that NPC heterogeneity, in some form, affects the recruitment of MX2.

**Figure 7—figure supplement 1.**
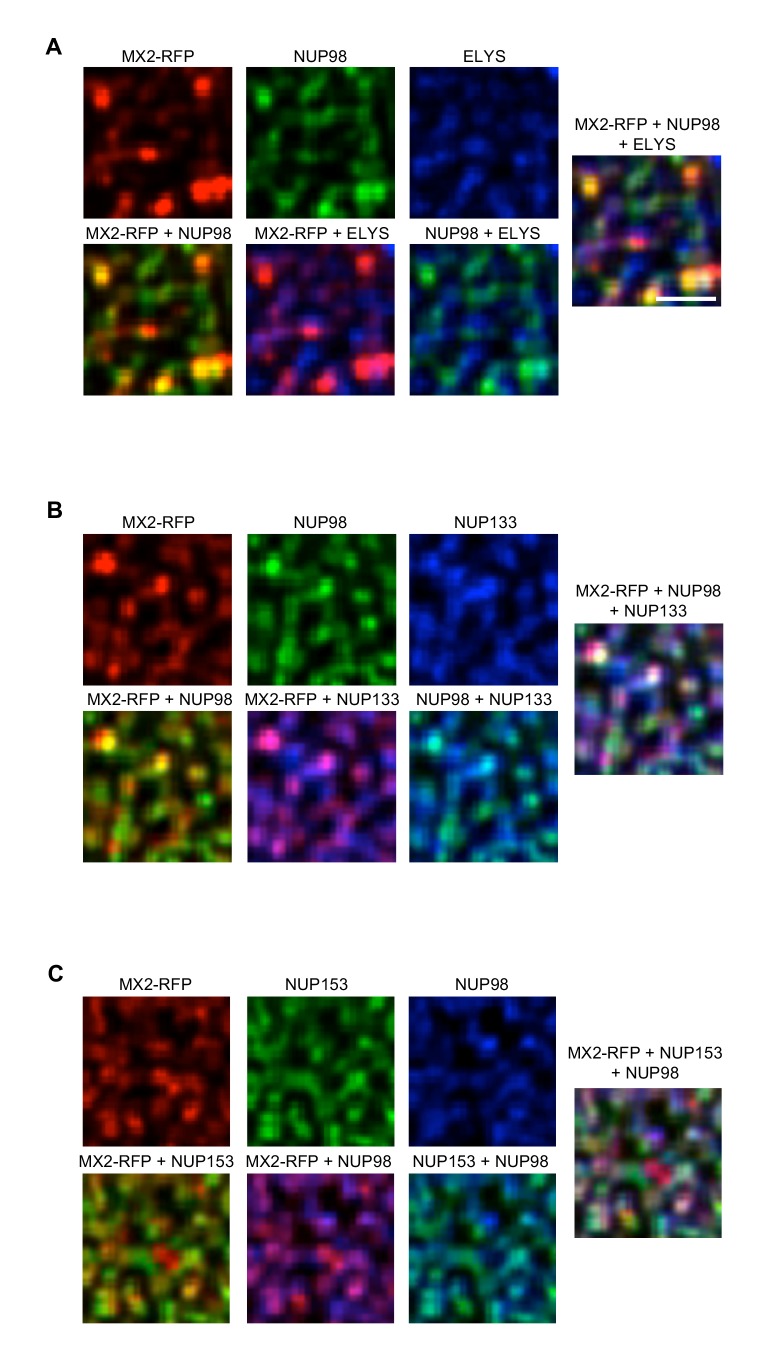
Localization of MX2 and Nups. Deconvolution microscopic images (single optical sections approximately coincident with the dorsal surface of the nucleus) of HeLa cells expressing MX2-RFP (red, stably transduced with a doxycycline inducible vector). Immunoflourescently stained (A) NUP98 (green), ELYS (blue); (**B**) NUP98 (green), NUP133 (blue); (**C**) NUP153 (green), NUP98 (blue). Green pseudocolored images were stained with AlexaFlour-488 secondary, blue pseudocolored images were stained with AlexaFlour-647 secondary. Scale bar = 10 μM. Representative of three independent experiments, with at least four images acquired per experiment.

**Figure 7—figure supplement 2.**
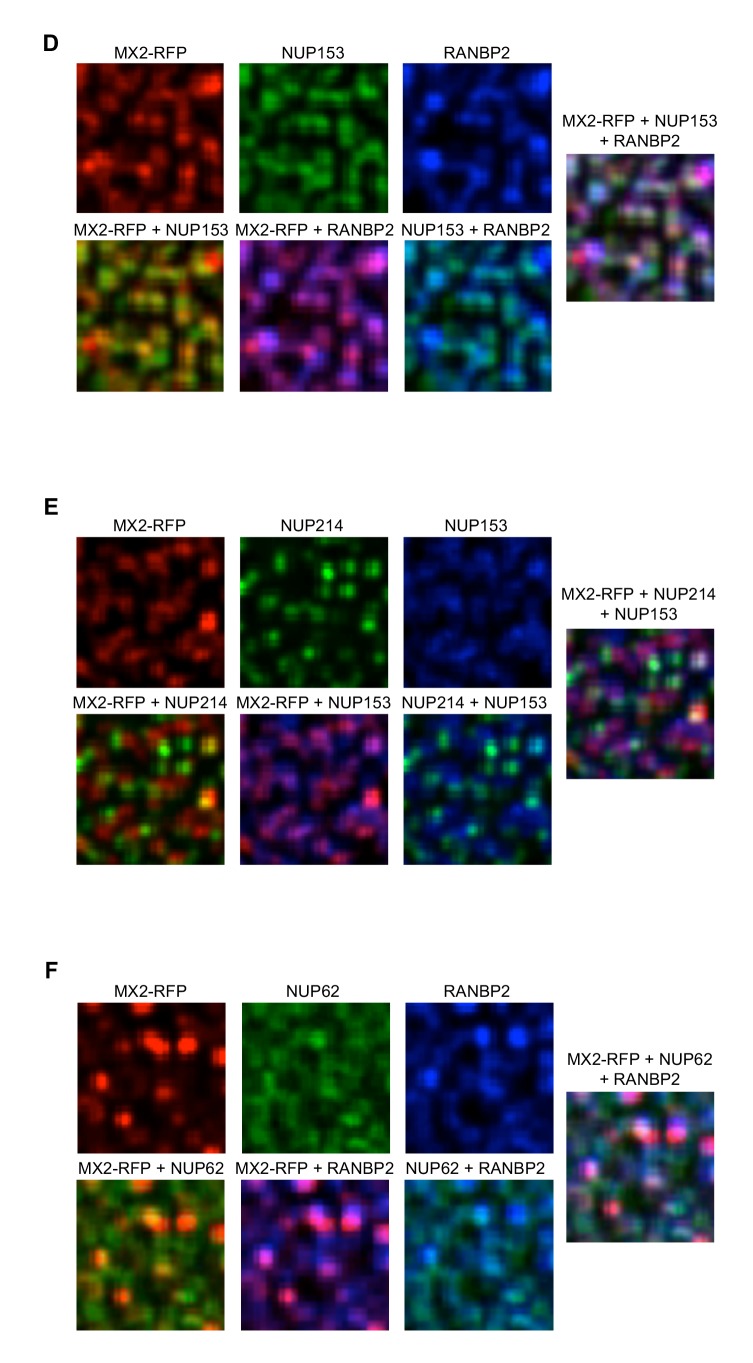
Localization of MX2 and Nups. Deconvolution microscopic images (single optical sections approximately coincident with the dorsal surface of the nucleus) of HeLa cells expressing MX2-RFP (red, stably transduced with a doxycycline inducible vector). Immunoflourescently stained (**D**) stained NUP153 (green), RANBP2 (blue); (**E**) NUP214 (green), NUP153 (blue); (**F**) NUP62 (green), RANBP2 (blue). Green pseudocolored images were stained with AlexaFlour-488 secondary (or NUP62-FITC conjugated primary), blue pseudocolored images were stained with AlexaFlour-647 secondary. Scale bar = 10 μM. Representative of three independent experiments, with at least four images acquired per experiment.

**Figure 7—figure supplement 3.**
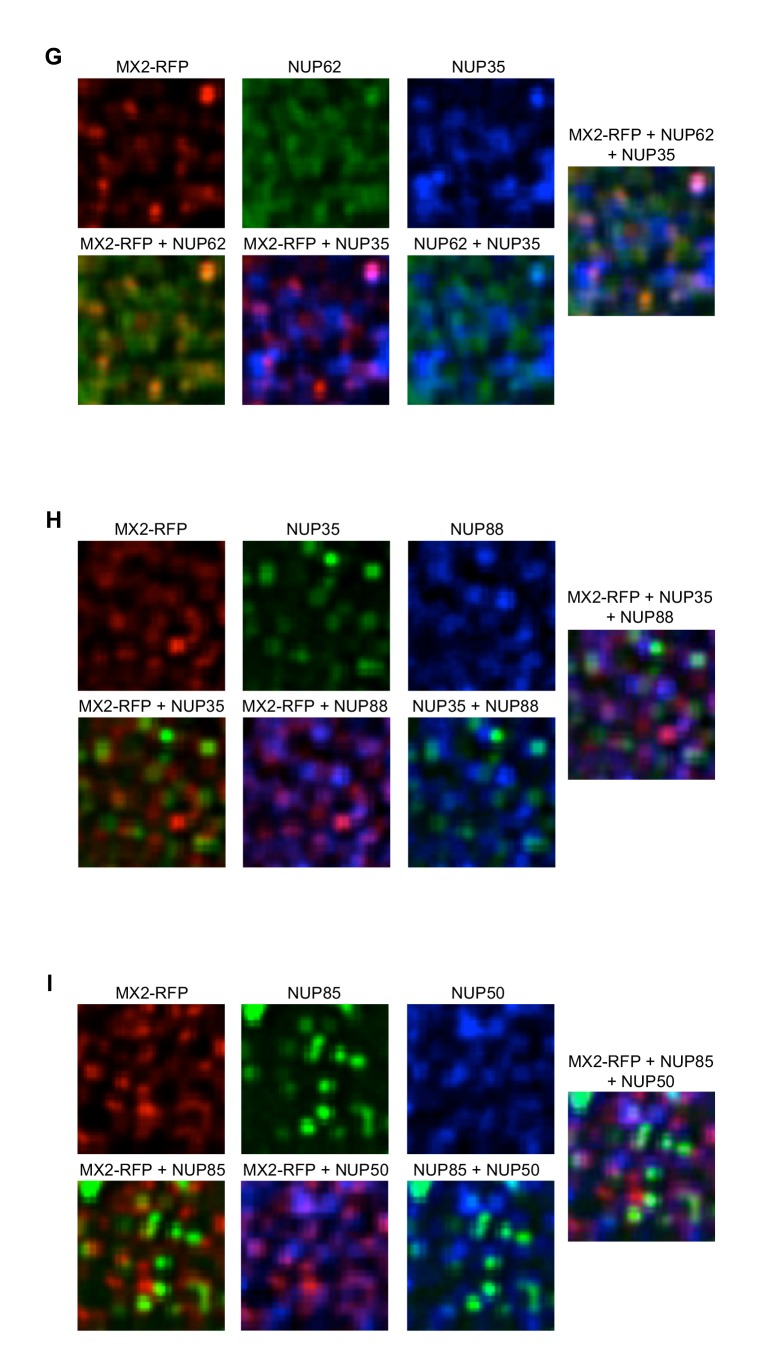
Localization of MX2 and Nups. Deconvolution microscopic images (single optical sections approximately coincident with the dorsal surface of the nucleus) of HeLa cells expressing MX2-RFP (red, stably transduced with a doxycycline inducible vector). Immunoflourescently stained (G) NUP62 (green), NUP35 (blue); (**H**) NUP35 (green), NUP88 (blue); (**I**) NUP85 (green), NUP50 (blue). Green pseudocolored images were stained with AlexaFlour-488 secondary (or NUP62-FITC conjugated primary), blue pseudocolored images were stained with AlexaFlour-647 secondary. Scale bar = 10 μM. Representative of three independent experiments, with at least four images acquired per experiment.

### Variable effects of Nup and NTR depletions on HIV-1 infection

**Figure 8.**
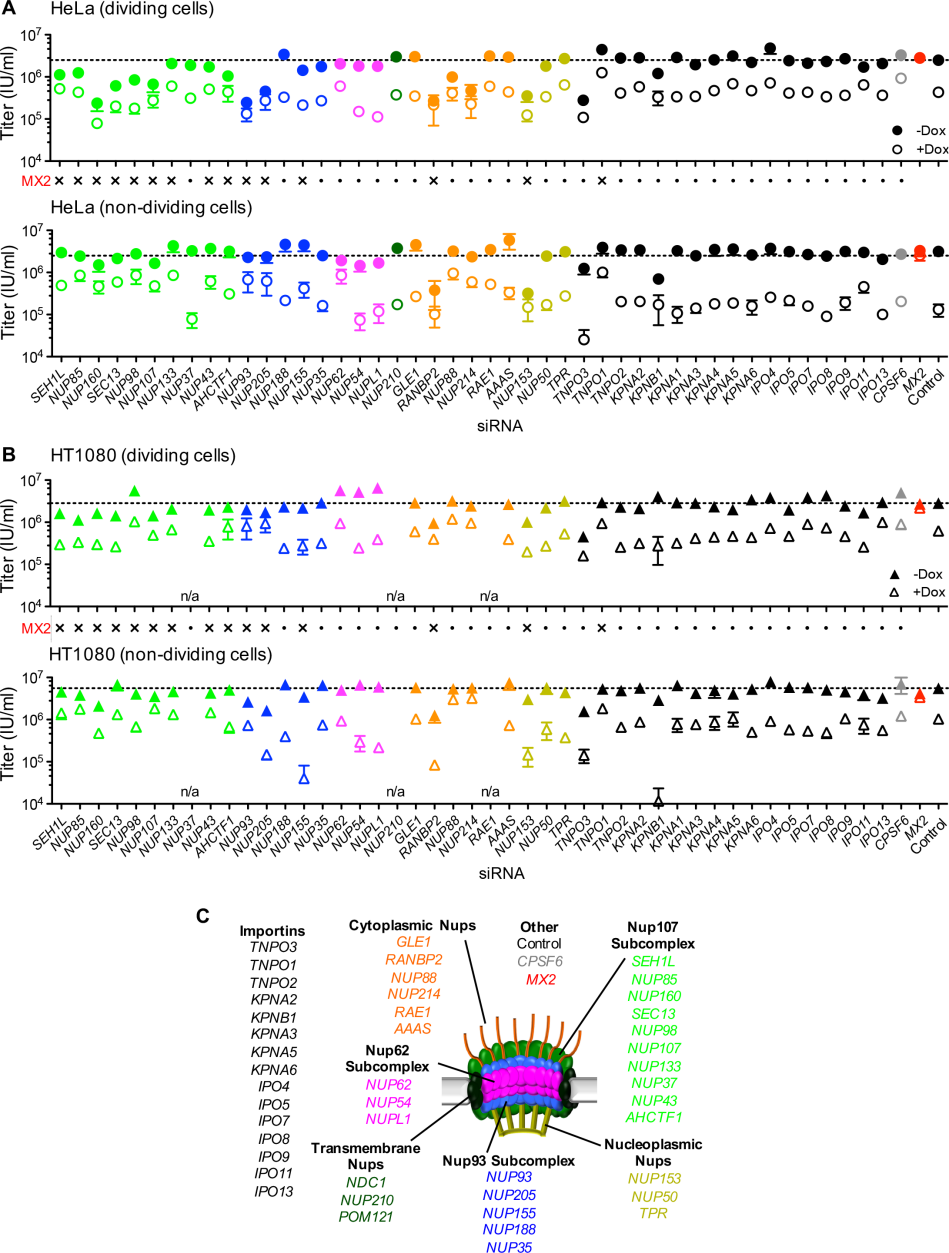
Effect of Nup and NTR depletion on HIV-1 infection and antiviral activity of MX2. (**A**) Infectivity of HIV-1 GFP reporter virus in dividing (top) and non-dividing (bottom) HeLa cells stably transduced with doxycycline-inducible MX2 in the presence (open circles) or absence (filled circles) of doxycycline 64 hr after transfection with siRNA (color coded by subcomplex as in Figure 4A). Middle, summary of localization of MX2 from immunoflouresence images in Figure 6. Aberrant localization following siRNA transfection in ≥~80% of cells is indicated by an ‘x’ and normal localization is indicated by a dot. Titers are mean ±sem, n = 3 technical replicates, representative of four independent experiments. (**B**) Infectivity of HIV-1 GFP reporter virus in dividing (top) and non-dividing (bottom) HT1080 cells stably transduced with doxycycline-inducible MX2 in the presence (open triangles) or absence (filled triangles) of doxycycline. Cells were infected 64 hr after transfection with siRNA (color coded by subcomplex as in Figure 4A). Middle, summary of localization of MX2 from immunoflouresence images in Figure 6. Aberrant MX2 localization following siRNA transfection in ≥~80% of cells is indicated by an ‘x’ and normal localization is indicated by a dot. Titers are mean ±sem, n = 3 technical replicates, representative of three independent experiments. n/a – not included due to insufficient knockdown (NUP37, RAE1) or not expressed (NUP210). (**C**) Schematic representation of the nuclear pore complex and target genes included in siRNA library color coded by subcomplex, as in Figure 4A.

To determine the effects of NPC perturbation on HIV-1 infection and MX2 activity, cells in which each individual Nup had been targeted with an siRNA were infected with a GFP-reporter virus. Depletion of a number of Nups affected HIV-1_WT_ infectivity, particularly in dividing HeLa cells (Figure 8A top). Indeed, with the notable exceptions of RANBP2 and NUP153, Nup depletions generally had a smaller effect on HIV-1_WT_ infection in non-dividing as compared to dividing HeLa cells (Figure 8A). A possible explanation for this could be the reduced pleiotropic effects of Nup depletion in non-dividing HeLa cells (Figure 5), due to the absence of cell division. Nevertheless, most knockdowns had significant effects on the levels of multiple Nups in both dividing and non-dividing cells. The effect of Nup depletion on HIV-1 infection did not always correlate with CA binding; for example, NUP62 knock-down had little effect on HIV-1 infectivity, despite a strong interaction with the CA tubes, while Nup107 bound only weakly to CA tubes (Figure 3), although its depletion did inhibit HIV-1 infection (Figure 8), presumably through effects on other Nups or perturbation of the overall structure of the NPC.

**Figure 8—figure supplement 1.**
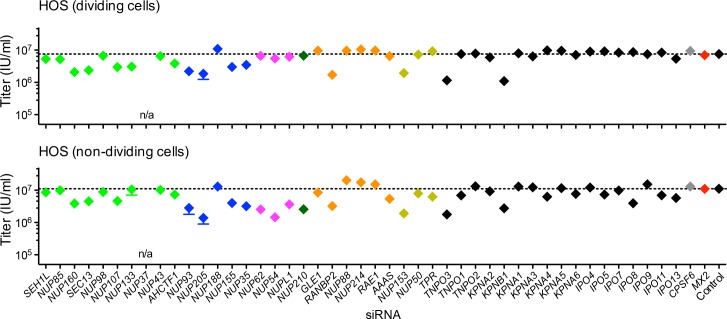
Effect of Nup and NTR depletion on HIV-1 infection in HOS cells. (**A**) Infectivity of HIV-1 GFP reporter virus in dividing (top) and non-dividing (bottom) HOS cells 64 hr after transfection with siRNA (color coded by subcomplex as in Figure 4A). Titers are mean ±sem, n = 3 technical replicates, representative of three independent experiments.

**Figure 8—figure supplement 2.**
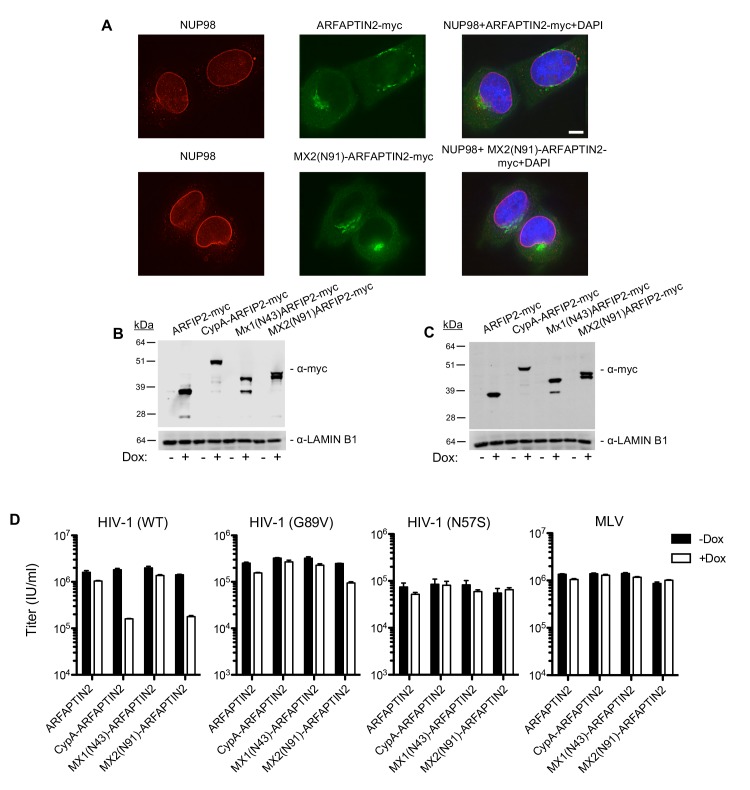
The amino-terminal domain of MX2 fused to a heterologous protein exhibits antiviral activity but with altered Nup requirements. (**A**) Deconvolution microscopic images (single optical sections) of immunoflourescently stained myc-tagged ARFAPTIN2 or MX2(N91)-ARFAPTIN2 fusion proteins (green), NUP98 (red), and DAPI-stained DNA. Optical sections are approximately through the center of the vertical dimension on the nucleus. Scale bar = 5 μm. (**B–C**) Western blot analysis of ARFAPTIN2 fusion protein expression (α-myc and α-LAMIN B1) in B) HeLa or C) HT1080 cells in the presence or absence of doxycycline. (**D**) Infection of HeLa cells stably transduced with doxycycline-inducible ARFAPTIN2 myc-tagged fusion proteins with various GFP reporter viruses in the presence (white bars) or absence (black bars) of doxycycline. Titers are mean ± sem, n = 3 technical replicates, representative of three independent experiments.

**Figure 8—figure supplement 3.**
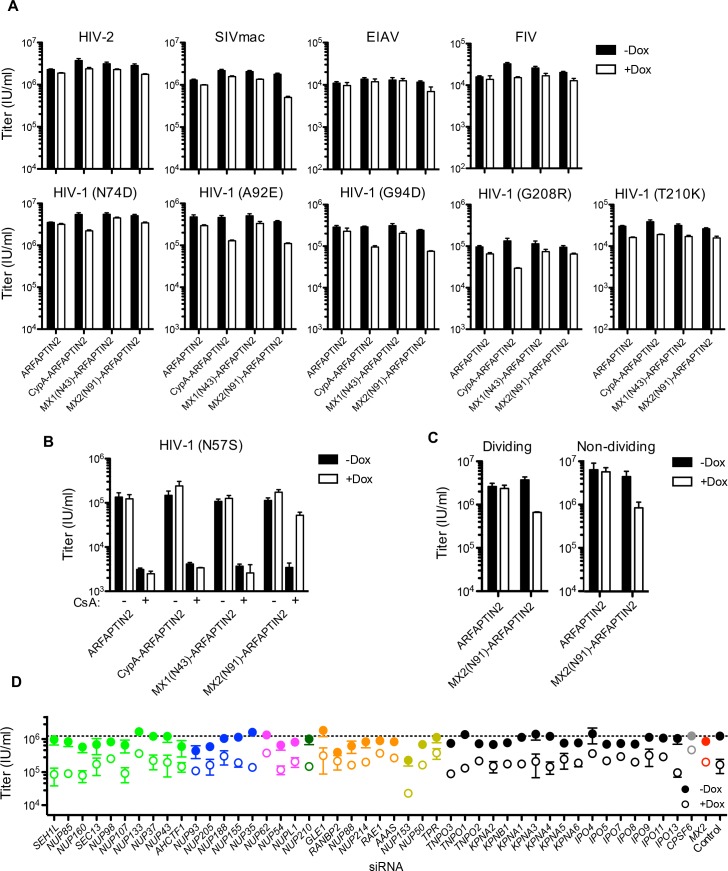
Effect of ARFAPTIN2 fusion protein expression on lentivirus and HIV-1 CA mutant infection. (**A**) Infection of HeLa cells stably transduced with doxycycline-inducible arfaptin2 myc-tagged fusion proteins with various GFP reporter viruses in the presence (white bars) or absence (black bars) of doxycycline. Titers are mean ± sem, n = 3 technical replicates, representative of three independent experiments. (**B**) Infectivity of HIV-1 N57S mutant GFP reporter virus in HT1080 cells stably transduced with arfaptin2 fusion proteins in the presence (white bars) or absence (black bars) of doxycycline and the presence or absence of CsA. Titers are mean ± sem, n = 3 technical replicates, representative of three independent experiments. (C) Infectivity of HIV-1-GFP reporter virus in dividing or non-dividing (aphidicolin treated) HeLa cells stably transduced with doxycycline-inducible arfaptin2 or MX2(N91)-arfaptin2 in the presence (open circles) or absence (filled circles) of doxycycline. Titers are mean ± sem, n = 3 technical replicates and are representative of three independent experiments. (**D**) Infectivity of HIV-1 GFP reporter virus in growth arrested (aphidicolin treated) HeLa cells stably transduced with doxycycline-inducible MX2(N91)-ARFAPTIN2 in the presence (open circles) or absence (filled circles) of doxycycline. Cells were infected 64 hr after transfection with siRNA (color coded by subcomplex as in Figure 4A). Titers are mean ± sem, n = 3 technical replicates, representative of three independent experiments.

**Figure 8—figure supplement 4.**
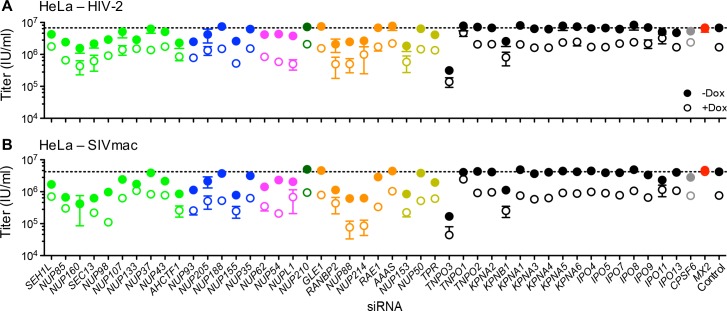
Effect of Nup and NTR depletion on primate lentivirus infection and MX2 sensitivity. Infectivity of (A) HIV-2 or (B) SIVmac GFP reporter virus in HeLa cells stably transduced with doxycycline-inducible MX2 in the presence (open circles) or absence (filled circles) of doxycycline. Cells were infected 64 hr after transfection with siRNA (color coded by subcomplex as in Figure 4A). Titers are mean ± sem, n = 3 technical replicates, representative of three independent experiments.

**Figure 8—figure supplement 5.**
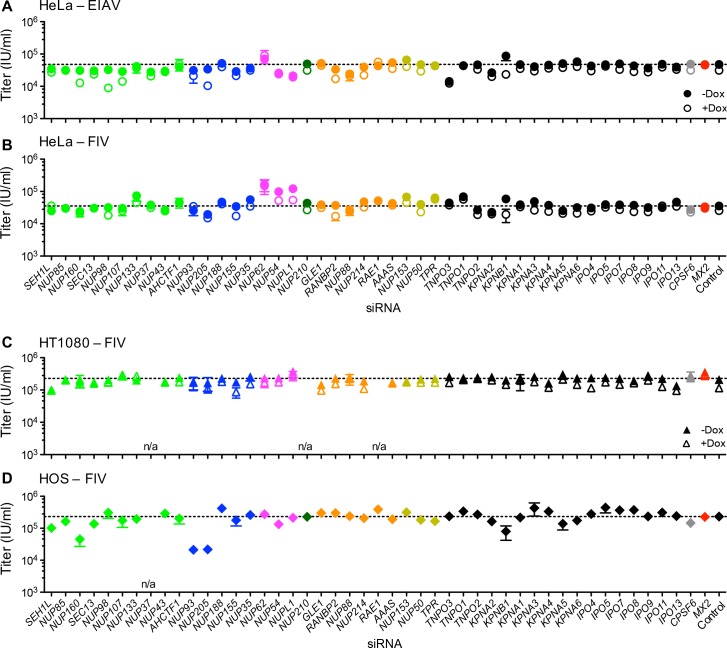
Effect of Nup and NTR depletion on non-primate lentivirus infection and MX2 sensitivity. Infectivity of (**A**) EIAV or (**B-D**) FIV GFP reporter virus in (**A-B**) HeLa or (**C**) HT1080 cells stably transduced with doxycycline-inducible MX2 in the presence (open symbols) or absence (filled symbols) of doxycycline, or (**D**) HOS cells. Cells were infected 64 hr after transfection with siRNA (color coded by subcomplex as in Figure 4A). Titers are mean ± sem, n = 3 technical replicates, representative of three independent experiments. n/a – not included due to insufficient knockdown (NUP37, RAE1) or not expressed (NUP210).

Comparison of the effects of Nup depletion on HIV-1 infection in HeLa, HT1080, and HOS cells revealed several notable differences, despite similar knockdown efficiencies (Figure 8 and Figure 8—figure supplement 1). Overall, HIV-1 appeared less sensitive to Nup depletion in HT1080 cells than in HeLa and HOS cells. Notably, the widely reported effects of RANBP2 and NUP153 on HIV-1 infection of HeLa cells (Figure 8A) were less evident in HT1080 cells (Figure 8B). Moreover, the larger effects of Nup depletion in dividing versus non-dividing HeLa cells were not observed in either HT1080 or HOS cells. In fact, several Nup depletions (NUP205, NUP62, NUP54, and NUPL1) had larger effects on HIV-1 infection in non-dividing as compared to dividing HOS cells (Figure 8—figure supplement 1).

### Nups are required for MX2 antiviral activity

A number of Nup depletions caused reduction in the anti-viral activity of MX2. Some depletions affected both the infectivity of HIV-1, and the antiviral activity of MX2 while other depletions did not reduce HIV-1 infectivity but did reduce MX2 activity. Curiously, there was not a clear correlation between whether a knockdown caused apparent perturbation of the subcellular localization of MX2 and whether it caused loss of antiviral activity (Figure 8A).

In HeLa cells, the effects of Nup depletion on the antiviral activity of MX2 were more pronounced in non-dividing compared to dividing cells (Figure 8A). Moreover, some Nup depletions (SEH1, NUP85, NUP160, SEC13, NUP98, NUP107, ELYS/ACHTF1, NUP93, NUP205, NUP88, and NUP214) that reduced both HIV-1 infection and MX2 activity in dividing HeLa cells, affected MX2 activity but not HIV-1 infection in non-dividing HeLa cells. A number of differences were evident in the Nup requirements for MX2 activity in HeLa and HT1080 cells. This was despite the fact that both cell lines exhibit similar Nup requirements for the subcellular localization of MX2 (Figures 6 and 8). Specifically, some Nup depletions that reduced the antiviral activity of MX2 in HeLa cells, did not affect its activity in HT1080 cells. Moreover, several Nup/NTR depletions (e.g. NUP155, NUP54, NUPL1, RANBP2, NUP153, and KPNB1) resulted in a dramatic increase in MX2 antiviral activity, specifically in non-dividing HT1080 cells, while having no effect or diminishing activity in HeLa cells. Overall, both HIV-1 infection and MX2 activity were affected by Nup perturbation, and these effects were clearly cell-type and cell-cycle dependent.

### Distinct Nup requirements for the antiviral activity of an MX2 amino-terminal domain fused to heterologous protein

Analyses of interspecies chimeric proteins have revealed that sequences near the MX2 N-terminus dictate anti-viral specificity (Busnadiego et al., 2014; Goujon et al., 2015), suggesting that this domain is responsible for binding to the HIV-1 capsid. Additionally, the MX2 N-terminus contains the NLS and is required for MX2 targeting to nuclear pores. The N-terminal 91 amino acids of MX2, that contain both the specificity determinants and the NLS, have been shown to confer anti-HIV-1 activity when transferred to proteins that form multimers (MX1 and FV1), but do not normally possess anti-HIV-1 activity (Goujon et al., 2015, 2014).

To determine whether the Nup requirement for MX2 activity was defined solely by the CA recognition determinants and NLS in the N-terminal domain, we appended the N-terminal 91 amino acids of MX2 to the dimeric cellular protein ARFAPTIN2. A similar dimeric fusion protein consisting of an HIV-1 CA-binding domain (CypA) fused to ARFAPTIN 2 (CypA-ARFAPTIN 2) has previously been shown to inhibit HIV-1 infection (Yap et al., 2007). Unlike MX2 and an MX2-MX1 chimera (Goujon et al., 2014), the MX2(N91)-ARFAPTIN 2 was not localized at the NPC, but rather appeared to localize at the trans Golgi network, similar to authentic ARFAPTIN2 (Peter et al., 2004) (Figure 8—figure supplement 2A).

HIV-1_WT_ was inhibited by MX2(N91)-ARFAPTIN2 and by a positive control CypA-ARFAPTIN2 fusion protein, but not ARFAPTIN2 alone or a fusion containing the N-terminal domain of MX1 (MX1(N43)-ARFAPTIN2) (Figure 8—figure supplement 2B–D). Other lentiviruses and HIV-1 CA mutants were partially or completely resistant to restriction by the MX2(N91)-ARFAPTIN2 fusion in a manner largely reflecting their relative sensitivity to full-length MX2 (Figure 8—figure supplements 2D and 3A). However, unlike full-length MX2, the MX2(N91)-ARFAPTIN2 fusion did not increase infectivity of HIV-1_G89V_, instead it marginally reduced HIV-1_G89V_ infection, Conversely, like authentic MX2, the MX2(N91)-ARFAPTIN2 fusion was able to rescue the HIV-1_N57S_ CA mutant from inhibition by CsA in HT1080 cells (Figure 8—figure supplement 3A,B). Moreover, the antiviral activity of the MX2(N91)-Arfaptin2 fusion was not enhanced by growth arrest in HeLa cells (Figure 8—figure supplement 3C). Overall, therefore, MX2(N91)-ARFAPTIN2 shared some of the properties of MX2 but differed in others.

Notably, the requirements for Nups/NTRs in the antiviral activity of MX2(N91)- ARFAPTIN2 differed from those for MX2. Inhibition of HIV-1 by MX2(N91)- ARFAPTIN2 was reduced by some Nup depletions, but depletion of several other Nup/NTRs that impaired MX2 activity (e.g. NUP107, NUP153, and TNPO1; Figure 8A) did not affect MX2(N91)- ARFAPTIN2 activity (Figure 8—figure supplement 3C). These observations suggest that MX2(N91)-ARFAPTIN2 occluded access of HIV-1 to nuclear DNA in an at least partly different manner to the authentic MX2 protein. Potentially these disparities could be due to differences in subcellular localization, or the result of distinctive interactions with NPC components.

### Distinct Nup requirements for infection and MX2 activity in the context of lentiviruses other than HIV-1

Since the sensitivities of the primate lentiviruses, HIV-2 and SIVmac, and the nonprimate lentiviruses, EIAV and FIV, to MX2 are distinct from HIV-1, we also investigated how Nup depletion altered their infectivity and susceptibility to MX2 activity. HIV-2 and SIVmac infection were inhibited by many Nup depletions and both were modestly sensitive to depletions of a number of Nups that did not affect HIV-1 infection (e.g. NUP155, TPR, NUP62, NUP54, NUPL1 (Figure 8—figure supplement 4). The MX2 sensitivity of HIV-2 and SIVmac was also differently affected by certain Nup depletions (e.g. NUP88, NUP214) as compared to HIV-1 (Figure 8—figure supplement 4).

Nup depletions generally had lesser effects on EIAV and FIV infection of HeLa cells as compared to primate lentiviruses (Figure 8—figure supplement 5A–B). Importantly, the reduced sensitivity of these viruses to Nup depletion indicates that sensitivity of the primate lentiviruses, HIV-1, HIV-2, and SIVmac (Figure 8, Figure 8—figure supplement 4) was not merely the result of non-specific effects on cell viability or physiology, but more likely due to selective effects on nucleocytoplasmic trafficking. Surprisingly, some Nup depletions that targeted members of the Nup62 subcomplex (NUP62, NUP54, NUPL1) increased FIV infection in HeLa cells. The majority of Nup depletions had little effect on MX2 resistance of FIV or EIAV, but some depletions marginally sensitized EIAV or FIV to inhibition by MX2 in HeLa cells (NUP205, NUP155, NUP54, NUPL1, KPNB1) (Figure 8—figure supplement 5A–B). FIV infectivity and MX2 resistance were also unaffected by most Nup depletions in HT1080 cells, but a small number of knockdowns (NUP93, NUP205, NUP160) were significantly deleterious to FIV infection in HOS cells (Figure 8—figure supplement 5C–D). These results highlight differences among lentiviruses in their Nup requirements for infection of human cells. However, these findings should not necessarily be interpreted as revealing the Nup requirements of FIV and EIAV in their natural hosts. The apparent resistance of FIV and EIAV to Nup depletion in human cells could reflect an inability to engage preferred Nups in human cells due to sequence differences from their natural hosts.

### Effects of Nup and NTR depletions on the infectivity, and MX2 and CypA sensitivity of HIV-1 CA mutants

**Figure 9.**
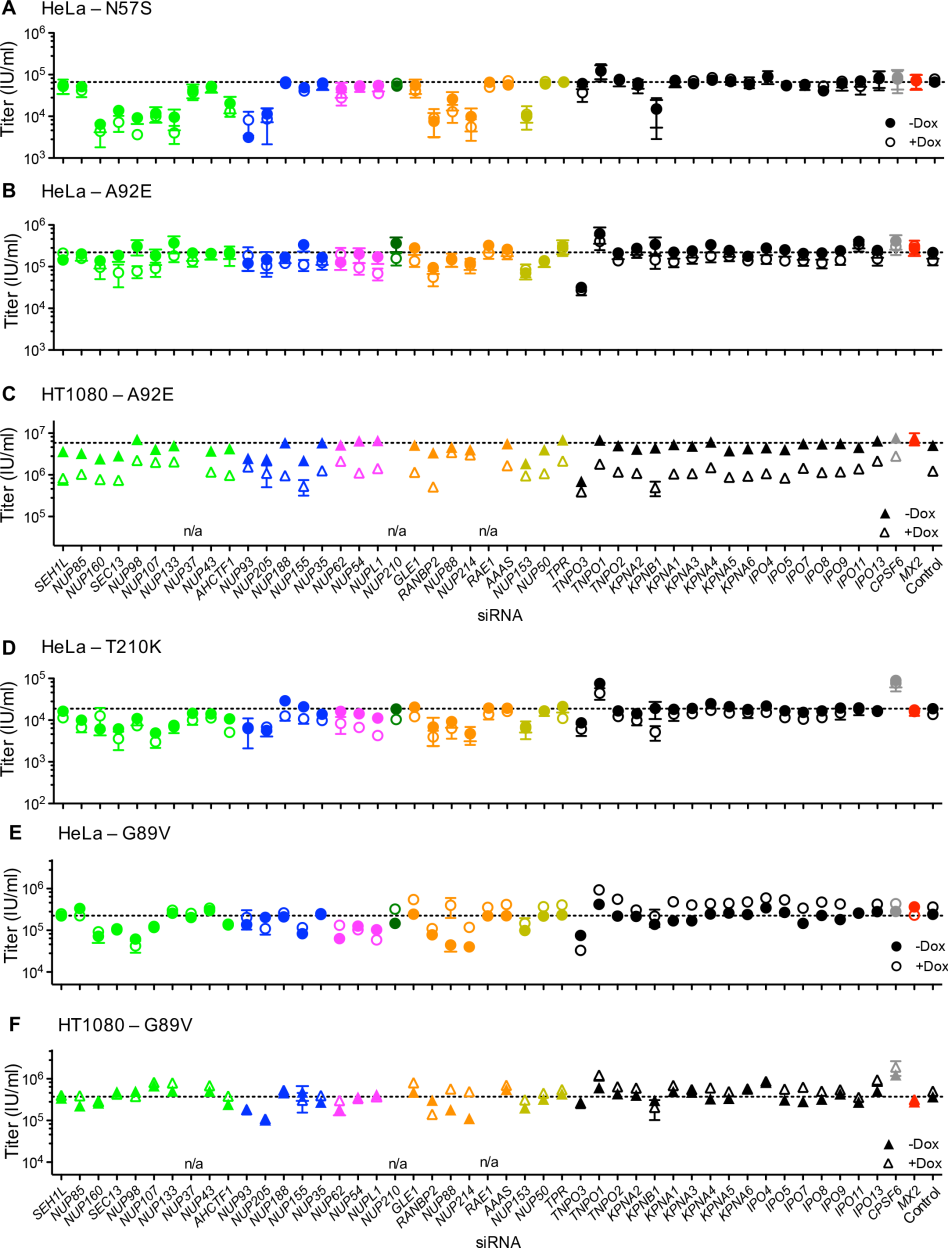
Effect of Nup and NTR depletion on HIV-1 CA mutant infection and MX2 sensitivity. Infectivity of HIV-1 CA mutant GFP reporter viruses in HeLa or HT1080 cells stably transduced with doxycycline-inducible MX2 in the presence (open symbols) or absence (filled symbols) of doxycycline, 64 hr after transfection with siRNA (color coded by subcomplex as in Figure 4A). Titers are mean ± sem, n ≥ 3 technical replicates, representative of three independent experiments. n/a – not included due to insufficient knockdown (NUP37, RAE1) or not expressed (NUP210).

Because the effects of CA mutation on HIV-1 infection and MX2 sensitivity varied with cell-type, we next investigated the effects of Nup depletion on selected HIV-1 CA mutants. Overall, there was substantial variability in the effects of Nup depletion on infection by CA mutants, as well as differential effects on CA mutants across the three cell lines (Figure 9 and Figure 9—figure supplement 1).

**Figure 9—figure supplement 1.**
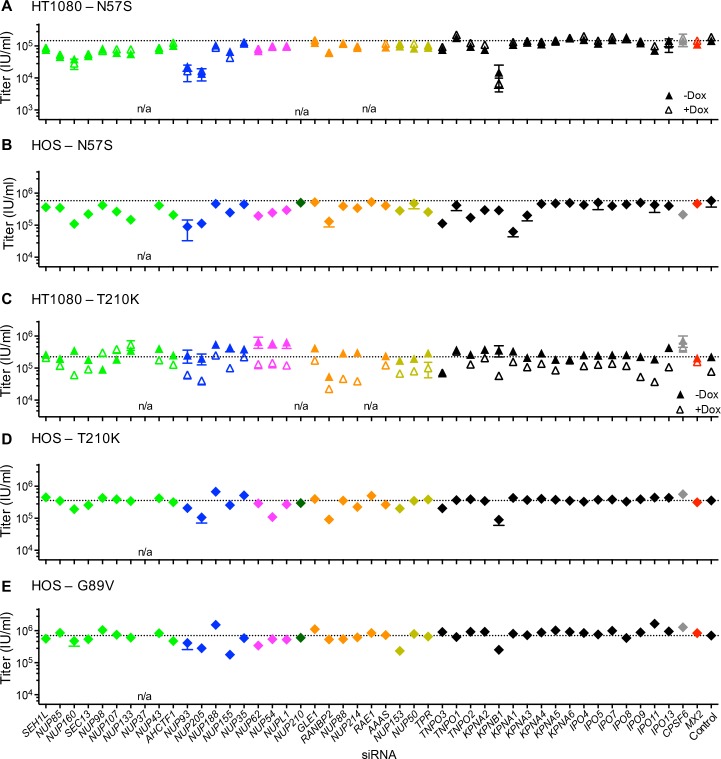
Effect of Nup and NTR depletion on HIV-1 CA mutant infection and MX2 sensitivity. Infectivity of HIV-1 CA mutant GFP reporter viruses in HOS cells or HT1080 cells stably transduced with doxycycline-inducible MX2 in the presence (open symbols) or absence (filled symbols) of doxycycline. Cells were infected 64 hr after transfection with siRNA (color coded by subcomplex as in Figure 4A). Titers are mean ± sem, n = 3 technical replicates, representative of three independent experiments. n/a – not included due to insufficient knockdown (NUP37, RAE1) or not expressed (NUP210).

The cell cycle-dependent CA mutant, HIV-1_N57S'_ should access target cell chromosomal DNA selectively during cell division and is therefore not expected to require intact nuclear pores for infection. However, HIV-1_N57S_ was highly dependent upon certain Nups (Figure 9A and Figure 9—figure supplement 1A and B). Indeed, the HIV-1_N57S_ mutant was sensitive to depletion of NUP133, particularly in HeLa cells, while WT virus was not affected by this perturbation (Figures 8A and 9A). Conversely, in several cases, the CA mutants exhibited reduced sensitivity to Nup depletion compared to the WT virus, particularly in HeLa cells. Note that these CA mutants exhibited reduced infectiousness compared to the WT virus, and so their reduced sensitivity to Nup depletion could reflect an inability to exploit Nups in the same way as HIV-1_WT_ rather than acquisition of the ability to use alternative pathways. Consistent with the idea that HIV-1 CA mutants fail to exploit Nups in the same way as HIV1_WT_, the HIV-1_T210K_, HIV-1_A92E_, and HIV-1_N57S_ CA mutants that were partially or completely resistant to MX2 in dividing HeLa cells, remained largely unaffected by MX2 upon Nup/NTR depletion in dividing HeLa cells (Figure 9A,B and D). Conversely, the HIV-1_A92E_ CA mutant that exhibits HIV1_WT_ levels of infectivity in HT1080 cells was sensitive to MX2 in HT1080 cells. This MX2 sensitivity was reduced by some Nup/NTR depletions (e.g. NUP93, NUP88, NUP214) (Figure 9C). Finally, the modest increase of HIV-1_G89V_ infectivity exerted by MX2 could be either amplified by some Nup depletions (e.g. NUP88, NUP214) or diminished by other Nup depletions (e.g. NUP54, NUPL1, RANBP2) (Figure 9E–F).

**Figure 10.**
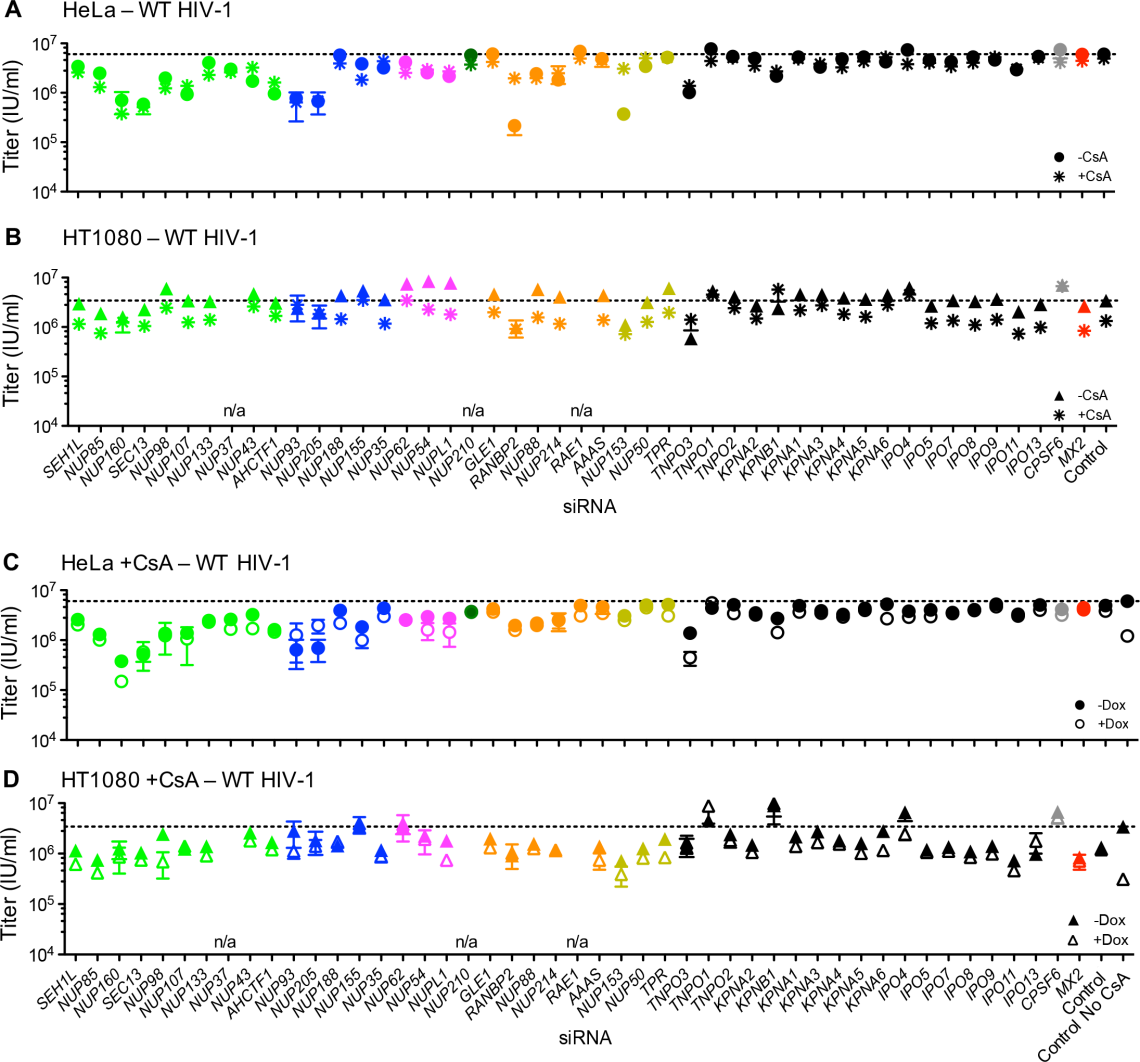
Effect of Nup and NTR depletion on HIV-1 infection and MX2 sensitivity in the presence of CsA. (**A–B**) Infectivity of HIV-1 GFP reporter virus in HeLa or HT1080 cells stably transduced with doxycycline-inducible MX2 in the absence of doxycycline and in the presence (asterisks) or absence (circles, HeLa or triangles, HT1080) of CsA. Cells were infected 64 hr after transfection with siRNA (color coded by subcomplex as in Figure 4A). (**C–D**) Infectivity of HIV-1 GFP reporter virus in HeLa or HT1080 cells stably transduced with doxycycline-inducible MX2 in the presence of CsA and in the presence (open symbols) or absence (filled symbols) of doxycycline. Cells were infected 64 hr after transfection with siRNA (color coded by subcomplex as in Figure 4A). Titers are mean ± sem, n = 3 technical replicates, representative of three independent experiments. n/a – not included due to insufficient knockdown (NUP37, RAE1) or not expressed (NUP210).

In most cases, CsA addition did not modify the effect of Nup perturbation on WT HIV-1 in HeLa cells. The exception to this general finding was that CsA dramatically reduced the deleterious effects of RANBP2 and NUP153 depletion on WT HIV-1 infection of HeLa cells (Figure 10A). Put another way, CsA rescued WT HIV-1 infection of HeLa cells when RANBP2 or NUP153 were depleted. Conversely, in HT1080 cells, CsA modestly reduced HIV-1 infectivity, and several Nup/NTR depletions (e.g. NUP93, NUP205, NUP155, RANBP2, NUP153, TNPO3, TNPO1, KPNB1, CPSF6) eliminated this effect (Figure 10B). The ability of CsA to rescue HIV-1 from the antiviral activity of MX2 was also partly dependent upon Nups; HIV-1 was rendered marginally sensitive to MX2 in CsA-treated HeLa or HT1080 cells when some Nups/NTRs were depleted (e.g. NUP160, NUP98, NUPL1, TNPO3, KPNB1) (Figure 10C–D).

**Figure 11.**
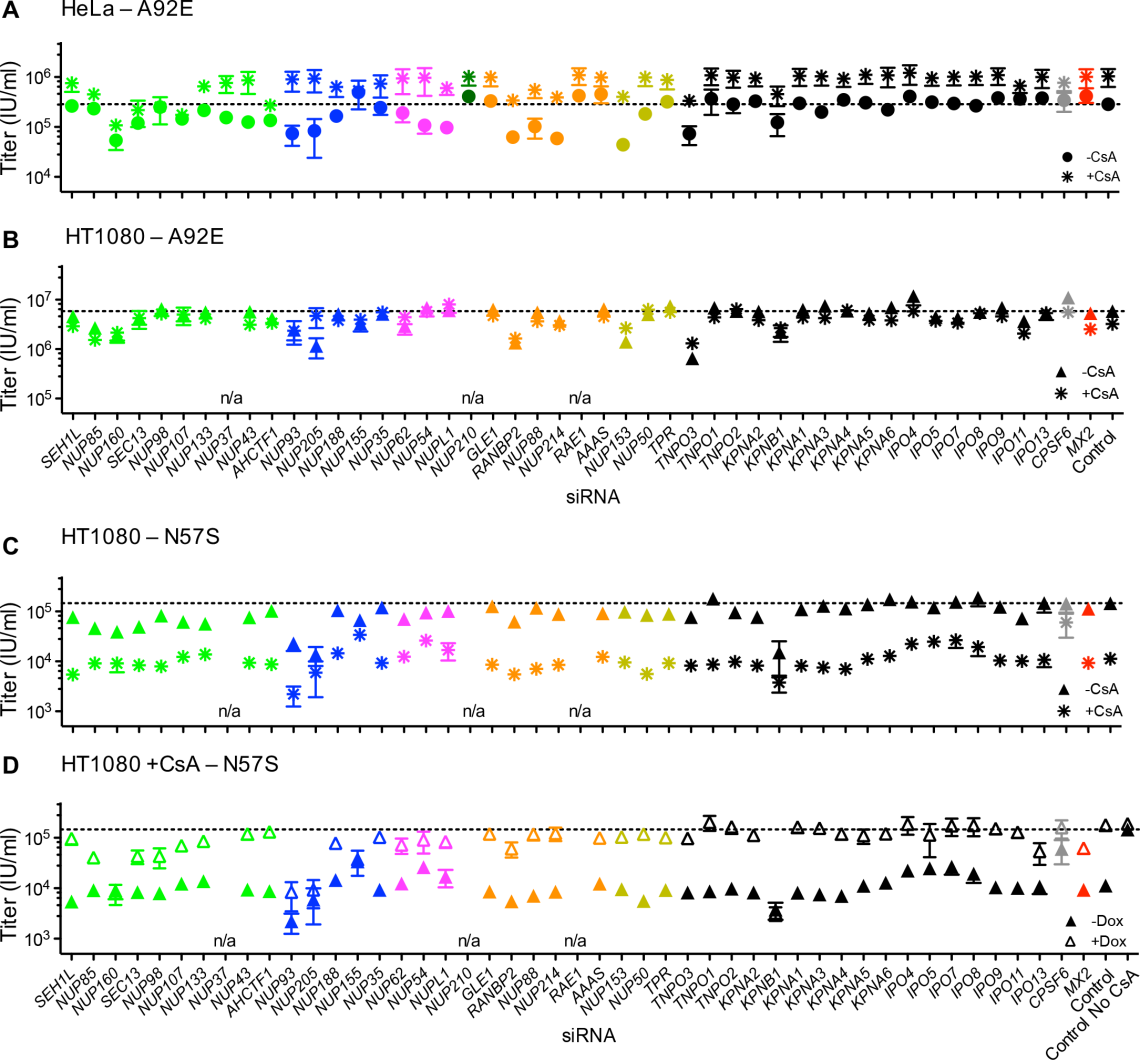
Effect of Nup and NTR depletion on HIV-1 A92E and N57S CA mutant infection in the presence of CsA. (**A–C**) Infectivity of HIV-1 A92E or N57S GFP reporter viruses in HeLa or HT1080 cells stably transduced with doxycycline-inducible MX2 in the absence of doxycycline and in the presence (asterisks) or absence (circles, HeLa or triangles, HT1080) of 5 μM CsA. Cells were infected 64 hr after transfection with siRNA (color coded by subcomplex as in Figure 4A). X-axis legend for A-B is below graph B, x-axis legend for C-D is below graph D. (**D**) Infectivity of HIV-1 N57S GFP reporter virus in HT1080 cells stably transduced with doxycycline-inducible MX2 in the presence of CsA and in the presence (open symbols) or absence (filled symbols) of doxycycline. Cells were infected 64 hr after transfection with siRNA (color coded by subcomplex as in Figure 4A). Titers are mean ± sem, n = 3 technical replicates, representative of three independent experiments. n/a – not included due to insufficient knockdown (NUP37, RAE1) or not expressed (NUP210).

Because the effect of CsA on HIV-1 infection was modified by CA mutations in different ways depending on the identity of the target cell, we tested whether Nup depletion altered the effects of CsA and/or MX2 on HIV-1 CA mutants. HIV-1_A92E_ is ‘CsA-dependent’ in HeLa cells, i.e. its infectivity is increased by CsA addition (Hatziioannou et al., 2005; Sokolskaja et al., 2004). The CsA dependence of HIV-1_A92E_ infection in HeLa cells was exaggerated by some Nup knockdowns (e.g. NUP93, NUP205, NUP54, NUP153) while other knockdowns reduced or eliminated the enhancing effects of CsA (e.g. NUP98, NUP107, NUP155) (Figure 11A). Conversely, the lack of a CsA effect on the HIV-1_A92E_ in HT1080 cells (Figure 1 and Figure 1—figure supplement 2) was not modified by Nup/NTR depletion, with one exception; specifically, NUP205 depletion caused HT1080 cells to exhibit the CsA-dependent phenotype characteristic of HeLa cells (Figure 11B). Notably, NUP205 was also among the Nups whose depletion exaggerated the CsA dependence exhibited by the HIV-1_A92E_ mutant in HeLa cells (Figure 11A).

Infection by HIV-1_N57S_ was strongly inhibited by CsA in HT1080 cells (Figure 1) and Nup depletions partly (e.g. NUP62, NUP54, NUPL1) or nearly completely (e.g. NUP155, NUP205) abolished this CsA sensitivity, (Figure 11C–D). CsA-inhibited HIV-1_N57S_ infection was rescued by MX2 (Figure 1). Nup/NTR knockdowns (e.g. NUP93, NUP205, KPNB1) inhibited this rescue activity. Surprisingly, MX2 knockdown itself did not completely eliminate its ability to rescue HIV-1_N57S_ from inhibition by CsA. This finding suggests that sub-detectable levels of MX2 are required for this effect. Indeed, under these MX2 knockdown conditions, MX2 antiviral activity was abolished (Figure 8B), and doxycycline addition alone did not rescue HIV-1_N57S_ infection (Figure 8—figure supplement 3B).

Overall, the positive and negative effects of CypA and MX2 on HIV-1 infection were clearly dependent on Nups. Moreover, there was significant overlap in the identity of Nups and NTRs that mediate MX2 and CypA effects on HIV-1 infection.

### MX2 can inhibit nuclear transport of nonviral cargos in an import pathway dependent manner

The variable effects of Nup perturbation on infection by HIV-1_WT_ and CA according to cell-line, cell-cycle and CsA treatment seemed inconsistent with the notion that HIV-1 exploits a single pathway (as defined by a particular set of Nups) to access host DNA. Moreover, different NLS sequences exhibited different Nup requirements (Figure 6—figure supplements 1 and 15–18) suggesting that different nuclear entry pathways have distinct dependencies on a given set of Nups. These findings, coupled with the fact that MX2 preferentially colocalized with certain Nups (Figure 7), led us to explore the hypothesis that MX2 preferentially inhibits specific nuclear import pathways, in particular those favored by HIV-1.

**Figure 12.**
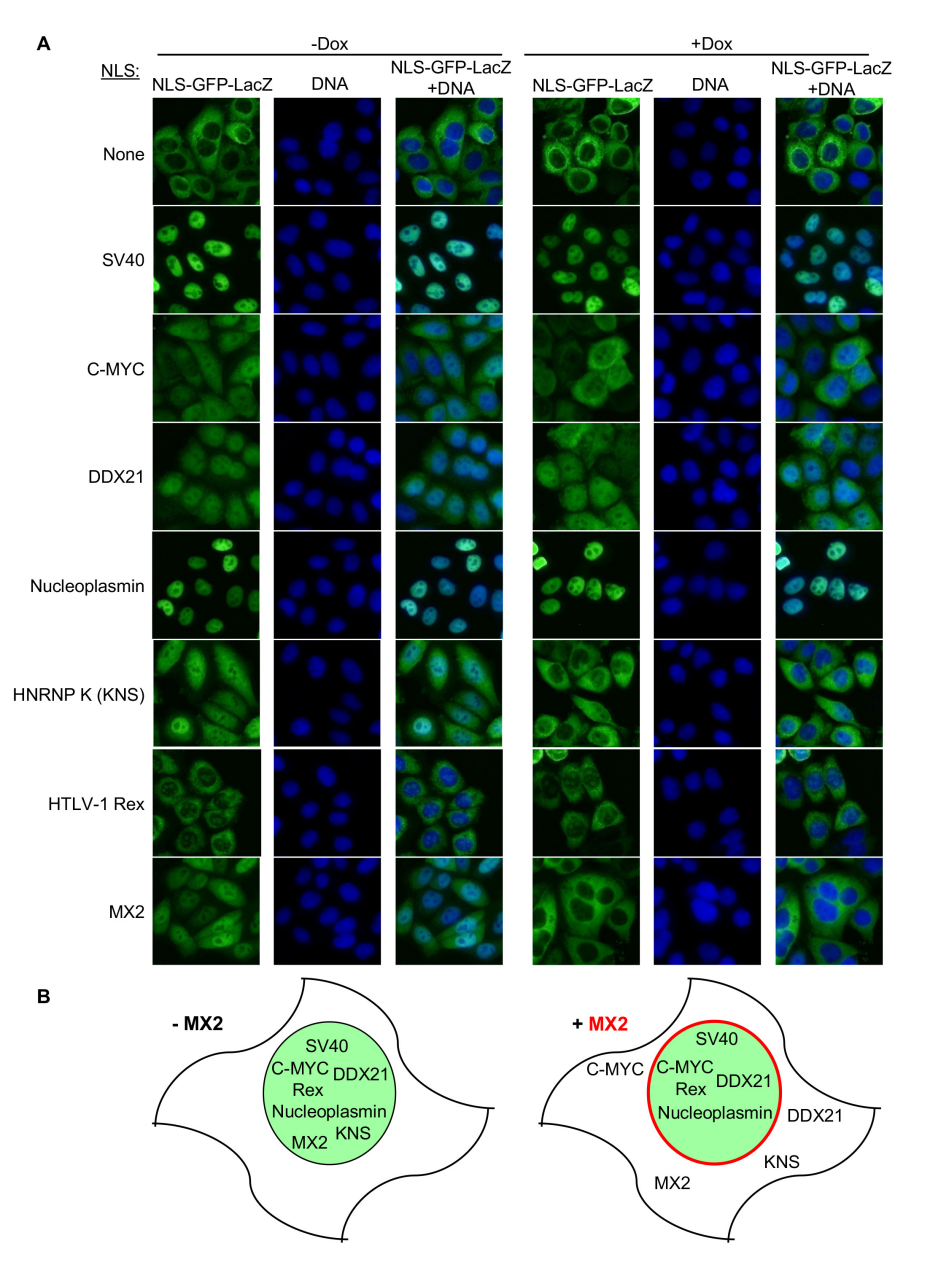
MX2 can inhibit nuclear transport of nonviral cargos in an import pathway dependent manner. (**A**) HeLa cells stably transduced with vectors expressing the indicated NLS-GFP-LacZ fusion protein (green) and doxycycline-inducible MX2-RFP, were untreated or treated with doxycycline to induce MX2-RFP prior to being fixed and stained with Hoechst. Representative of three independent experiments, with at least three images acquired per experiment. (**B**) Schematic representing NLS-GFP-LacZ fusion location in the presence or absence of MX2-RFP (indicated in red).

Therefore, we next tested whether MX2 could disrupt specific cellular nuclear import pathways as defined by different NLS sequences. To this end, we attached various NLS signals (Table 1) to a GFP-LacZ fusion protein and monitored whether MX2 expression affected the localization of each NLS-GFP-LacZ variant (Figure 12). Appended NLS sequences caused nuclear accumulation of GFP-LacZ to varying degrees. MX2 did not affect the nuclear import driven by canonical NLS sequences found in SV40, Nucleoplasmin, or HTLV-1 Rex, and only marginally inhibited DDX21 NLS driven import (Figure 12) suggesting that MX2 does not markedly inhibit nuclear import pathways used by these NLS sequences. In contrast, the accumulation GFP-LacZ in the nucleus driven by NLS signals from C-MYC, and especially HNRNP K, as well as the NLS from MX2 itself, was clearly inhibited by MX2. Thus, these data indicate that MX2 does not appear to induce a global block to nuclear import but can inhibit the movement of a non-viral cargo protein from the cytoplasm to the nucleus, in a manner that is highly dependent on the nature of the NLS, and by inference, the ‘pathway’ taken into the nucleus.

**Table 1.** Nuclear localization signals fused to GFP-LacZ

**Parent protein**	**NLS-type**	**Reference(s)**	**Cloning method**
SV40 Large T Antigen	Monopartite class 1	(Mattaj and Englmeier, 1998)	oligonucleotide annealing
C-MYC	Monopartite class 2	(Kosugi et al., 2009)	oligonucleotide annealing
DDX21	Monopartite class 3	(Kosugi et al., 2009)	oligonucleotide annealing
Nucleoplasmin	Bipartite	(Mattaj and Englmeier, 1998)	oligonucleotide annealing
HNRNP K (KNS)	n/a	(Michael et al., 1997)	PCR amplification
HTLV-1 Rex	n/a	(Siomi et al., 1988; Slamon et al., 1988)	oligonucleotide annealing
MX2	n/a	(Melén et al., 1996)	PCR amplification

## Discussion

While many viruses enter the nucleus of their host cells, retroviruses have an additional requirement to integrate into the host chromatin to enable their replication. Nups may therefore be required both for passage across the nuclear envelope, and for access to the host chromatin. Herein, we employed a panel of HIV-1 CA mutants, inhibitors of cell cycle progression and CypA:CA interactions, along with a systematic knock-down of Nups and NTRs to comprehensively investigate the functional interplay between Nups, MX2, CypA, and the viral capsid during HIV-1 infection.

Findings described herein that are crucial for interpreting the effects of individual Nup/NTR knockdown experiments include: (i) the HIV-1 CA physically binds to multiple Nup proteins, (ii) the levels of individual Nups vary considerably among different cell types and apparently among individual NPCs, (iii) depletion of one Nup often has pleiotropic effects, changing the levels and localization of other NPC components. These findings lead to the conclusion that assigning a direct role of a single Nup in HIV-1 infection or MX2 activity likely cannot be unambiguously achieved using a knockdown/knockout approach. Nevertheless, we could clearly demonstrate circumstances in which manipulating Nup levels (and presumably NPC composition) affected HIV-1 infection and MX2 activity. There was an unexpected and striking lack of consistency in the apparent Nup requirements for infection by HIV-1_WT_ and HIV-1 CA mutants among three cell lines. Our data strongly suggest that variable Nup levels and NPC composition very likely underlie the variable effects of CA mutations, as well as CsA and CypA on HIV-1 infection. For example, CsA susceptibility and dependence (that clearly varies according to CA sequence and target cell type) could be induced or abolished by manipulating NPC composition.

Such findings are best explained by invoking the existence of multiple different, perhaps overlapping, pathways (‘pathways’ defined as the utilization of a particular set of Nups) that direct HIV-1 preintegration complexes through the NPC and to host chromatin. These pathways would be differentially available for use in different cell types (as dictated by Nup levels), and used with different efficiencies according to CA sequence and CA:CypA interaction. Differential CPSF6 binding to CA mutants (and the impact of CypA thereon) likely adds a further layer of complexity. Our finding that the cell cycle-dependent HIV-1_N57S_ CA mutant retains a requirement for Nups during infection indicates that Nup-dependent pathways could facilitate HIV-1 infection even when the nuclear envelope is dismantled during mitosis. In fact, accumulating evidence suggests that Nups play key roles in the life of cells during mitosis, including maintenance of centrosome association with the nuclear envelope, mitotic spindle formation, chromosome segregation, as well as NPC reassembly at mitotic exit (reviewed in (Wozniak et al., 2010)). HIV-1 may therefore access host DNA during mitosis by interacting with chromatin-associated Nups prior to reassembly of the nuclear envelope.

While the cell lines used herein are not the natural targets of HIV-1 infection, our results suggest that the in vivo utilization of Nups by HIV-1 and MX2 could vary among cell types (e.g. T cells vs. macrophages or naïve vs. memory vs. effector CD4^+^ T cells). As well as variability in the composition of NPCs among cell types, there is also likely to be compositional variation within a given cell type, or even within a single cell. Indeed, NPC assembly between anaphase and telophase occurs within a few minutes and appears to be rate-limited by ELYS-mediated recruitment of the Nup107 subcomplex to chromatin (Dultz et al., 2008; Franz et al., 2007; Gillespie et al., 2007; Rasala et al., 2006). In contrast, NPCs that are assembled during interphase do so over up to an hour and in an ELYS-independent manner (D'Angelo et al., 2009; Doucet and Hetzer, 2010). Although a definitive appraisal of compositional variability of the NPC does not yet exist, as a modular structure with considerable flexibility in the arrangement of the composite elements, conformational variability almost certainly differentiates individual pores and affects their transport capacity [reviewed in (Knockenhauer and Schwartz, 2016)]. Each of these possible sources of variation in NPCs could contribute to inconsistency between Nup requirements for HIV-1 infection and MX2 activity between cell lines and could underlie the increased antiviral activity of MX2 in some non-dividing cells. Finally, it is also possible that sequence variation between Nups and/or NTRs contributes to the dissimilarities among cell lines uncovered herein.

Given the aforementioned complexities in NPC composition, and our finding the MX2 activity is dependent on Nups, understanding how MX2 inhibits HIV-1 infection is similarly complex. While several studies have generated or uncovered MX2 ‘resistant’ HIV-1 CA mutants, (Busnadiego et al., 2014; Goujon et al., 2013; Kane et al., 2013; Liu et al., 2013; Liu et al., 2015) our findings show that MX2 resistance is context dependent, and that none of these ‘MX2-resistant’ mutants are entirely unaffected by MX2. Indeed, we found that CA mutations, cell-type, cell-cycle, and CypA can all modify the effects of MX2 in previously undescribed ways. We found multiple instances in which MX2 acted to increase, rather than inhibit infection of an HIV-1 CA mutant, in a manner which indicates that the outcome of the interaction between MX2 and the viral CA is dependent upon Nups and CypA.

We also found that MX2 can inhibit the import of a non-viral reporter protein directed to the nucleus by cellular NLS sequences. Crucially, MX2 differentially inhibited import driven by different NLS sequences from non-viral proteins, suggesting that MX2 can inhibit certain nuclear transport pathways more effectively than others. Together, these data suggest that the antiviral activity of MX2 is governed by the propensity of HIV-1 to favor particular nuclear entry pathways (‘pathways’ defined by the utilization of particular Nups) and the propensity of MX2 to preferentially inhibit those pathways. Thus, the overall antiviral activity of MX2 would be determined by (i) the availability of the pathways (defined by cell-type and cell-cycle i.e. Nup and NPC levels), and (ii) the propensity of HIV-1 to use the available pathways (defined by HIV-1 CA sequence and interaction of CA-binding host proteins, e.g. CypA or CPSF6).

Although MX2 is clearly dependent upon Nups for antiviral activity, our knockdown data suggest that nuclear pore localization is not sufficient (and perhaps not essential) for antiviral activity. Depletion of some Nups accentuated the antiviral activity of MX2 or accentuated the ability of MX2 to increase infectivity. MX2 co-localized with some Nups to a greater degree than others, raising the possibility that MX2 is enriched at NPCs that are preferentially utilized by HIV-1. Knockdown of a given Nup could deplete the preferred, MX2-enriched NPCs, or eliminate alternative, non-MX2-containing NPCs. Alternatively, MX2-inhibited interactions between Nups and the viral CA required for nuclear entry may not be restricted to the nuclear envelope, and may also occur in the cytoplasm; in fact, CPSF6-dependent RANBP2 interactions with the viral capsid have been reported to occur in the cytoplasm (Dharan et al., 2016). The dependence of the MX2(N91)-ARFAPTIN2 fusion on Nups for antiviral activity, even though MX2(N91)-ARFAPTIN2 is not concentrated at NPCs, is also suggestive of potential cytoplasmic interactions between the viral capsid and Nups.

The crystal structure of MX2 reveals that it forms an extended antiparallel dimer (Fribourgh et al., 2014), and dimerization is critical for viral restriction (Dicks et al., 2016; Fribourgh et al., 2014). Modeling suggests that the stalk domains provide the appropriate spacing to interact with hexamer interfaces in the HIV-1 CA lattice (Fribourgh et al., 2014), However, It is also possible that one MX2 subunit in the dimer interacts with the NPC, while another interacts with the viral CA. Such a model would provide an explanatory basis for the observation that MX2 can enhance or rescue HIV-1 infection in some instances. Alternatively, interactions with the NPC may simply concentrate MX2 at a location where it is likely to encounter the incoming capsid cores. In either case, the outcome of MX2-CA interactions is clearly dependent upon CypA and NPC composition.

A subset of Nup depletions exhibited particularly interesting or complex effects on HIV-1 infection. Their effects on cell-type, cell-cycle, viral CA, and CypA dependent phenotypes in viral infection and MX2 activity suggest that their levels, or their presence in individual NPCs, play key direct or indirect roles in determining the availability of pathways by which HIV-1 accesses the nucleus and/or target cell chromatin.

### NUP155 and the Nup93 complex

NUP155 is part of the Nup93 subcomplex that contains NUP93, NUP205, NUP155, NUP188, and NUP35 (Figure 4A). Structural analyses of human and *C. thermophilum* nuclear pores indicate that NUP155 exists both buried within the inner ring of the nuclear pore, and as a link between the inner and outer rings, where it is exposed in the bridge between the two rings (Kosinski et al., 2016; Lin et al., 2016). Multiple structural conformations of the *C. thermophilum* homologue of NUP155 (NUP170) have been observed (Lin et al., 2016), raising the possibility that differences in NUP155 conformation could underlie structural heterogeneity among individual nuclear pores. Interestingly, the relative levels of individual components of the Nup93 complex varied among cell lines. For example, NUP155 protein levels were low in T-cell and myeloid cell lines compared to the adherent cells. These findings suggest that the composition of the Nup93 complex is variable among cell types. NUP155 depletion had little effect on the levels of other Nups, but likely causes changes in nuclear pore composition, as its depletion induced clear mislocalization of NUP62, NUP214, and RANBP2.

While WT HIV-1 infection of HeLa cells was not impeded by NUP155 depletion, HIV-2 and SIVmac infection was inhibited. NUP155 depletion also caused mislocalization of MX2 in both HeLa and HT1080 cells. Strikingly, while NUP155 depletion marginally reduced MX2 antiviral activity against HIV-1 (~2 fold) in non-dividing HeLa cells, it markedly enhanced (by 17-fold) anti-HIV-1 MX2 activity in non-dividing HT1080 cells (Figure 8). In this respect, NUP155 depletion rendered HT1080 cells more similar to HeLa cells: specifically, MX2 activity was not increased by growth arrest in unmanipulated HT1080 cells, but was enhanced by growth arrest in HeLa cells and NUP155-depleted HT1080 cells.

Perturbation of the NUP155 and the Nup93 complex also impacted the effect of CA mutations and CypA on HIV-1 infection. In particular, NUP155 depletion nearly abolished the CsA-dependent phenotype of HIV-1_A92E_ in HeLa cells, while depletion of certain other Nup93 complex components (specifically NUP93 itself or NUP205) accentuated the CsA dependence of HIV-1_A92E_. Moreover, NUP205 depletion caused HIV-1_A92E_ infection to become CsA-dependent in HT1080 cells. In this respect, NUP205 depletion again made HT1080 cells behave more like HeLa cells. Moreover, the striking ability of CsA to strongly inhibit HIV-1_N57S_ infection in HT1080 cells (that was not evident in HeLa cells) was nearly completely abolished by NUP155 depletion (Figures 1 and C). Additionally, the ability of MX2 to rescue HIV-1_N57S_ infection from inhibition by CsA in HT1080 cells was reduced by NUP93 and NUP205 depletion (Figure 11D). NUP155 did not bind CA tubes in vitro (Figure 3), suggesting that its effects on HIV-1 infection are indirect.

Overall, manipulations of NUP155 and other Nup93 subcomplex components recapitulated, abolished, or otherwise modified several of the key cell-type- and CA-dependent differences in the effects of CypA and MX2 on HIV-1 infection. These results suggest that the Nup93 subcomplex is a key regulator of the functional interaction between the HIV-1 CA and the nuclear pore complex, and that variation in the composition of this complex among cell types or during the cell cycle could underlie several of the discrepant effects of CA mutations, CypA, and MX2 on HIV-1 infection.

### The Nup62 complex

The Nup62 subcomplex in the central channel of the pore consists of NUP62, NUP54, and NUP58 (NUPL1). Little sequence similarity is evident among orthologous members of the Nup62 complex in evolutionarily divergent species, but its overall structure is well conserved, as are multiple interactions among the components (Chug et al., 2014; Stuwe et al., 2015). Additional interactions anchor the Nup62 subcomplex to the nuclear pore scaffold and regulate transport (Chug et al., 2015; Knockenhauer and Schwartz, 2016; Yoshimura et al., 2013). The Nup62 complex forms an elongated, rigid structure, with FG domains extending into the channel of the pore forming a barrier on an elastic anchor that may allow for long-range movements to accommodate large cargos (Chug et al., 2015).

NUP62 depletion caused the levels of several other Nups to be moderately reduced, and depletion of any single member of the Nup62 complex dramatically reduced the levels of the other two members (Figure 5 and Figure 5—figure supplements 1–6). However, NUP62 depletion did not result in the mislocalization of any of the other Nups tested. NUP62 depletion did not significantly inhibit HIV-1_WT_ infection of HeLa or HT1080 cells but caused a four-fold decrease of infection of non-dividing HOS cells. In a few instances, (e.g. HIV-1_WT_ infection of HT1080 cells and FIV infection of HeLa cells) depletion of Nup62 complex components modestly increased infection.

While NUP62 depletion did not cause mislocalization of MX2, its antiviral activity was reduced in dividing NUP62-depleted HeLa cells and, strikingly, nearly abolished in non-dividing NUP62-depleted HeLa cells (Figure 8A). Unlike HIV-1_WT_, HIV-1_G89V_ infection was modestly reduced (four-fold) upon NUP62 knockdown in HeLa cells, but HIV-1_G89V_ infection was restored by MX2 expression in NUP62 depleted HeLa cells (Figure 9E). Depletion of other NUP62 complex components had different effects on MX2 activity. Specifically, NUP54 and NUP58 depletion enhanced MX2 antiviral activity against HIV-1_WT_ in both HeLa and HT1080 cells (Figure 8). In contrast to the striking effects on MX2 activity, manipulation of the Nup62 complex had only modest effects on CypA/CsA modulation of HIV-1 infection (Figures 10 and 11).

That manipulation of the Nup62 complex had both positive and negative effects on MX2 activity against HIV-1, without affecting MX2 localization, suggests that it participates in HIV-1 infection. Consistent with this idea, NUP62 was precipitated with CA tubes, and in fact was depleted from cell lysates by CA tubes, suggesting that it interacts physically (either directly or indirectly) with CA. NUP62 has also been reported to bind HIV-1 integrase and play a significant role in viral integration in C8166 cells (Ao et al., 2012) suggesting that depending on the cell type, NUP62 may play a role in both nuclear entry and viral integration.

Structural analysis indicates that the NUP62 complex forms an elongated, rigid structure, with FG domains extending into the channel of the pore forming a barrier on an elastic anchor that may allow for long-range movements to accommodate large cargos (Chug et al., 2015). One possibility is that MX2 affects the movement of this trimeric complex within the channel of the pore, or the allosteric interactions between the Nup62 complex members (Koh and Blobel, 2015a) thereby altering CA interactions with the NPC.

### RANBP2

RANBP2 encodes multiple domains, including several FG repeats, four Ran-binding domains, a SUMO E3-ligase domain, a RANGAP interacting domain, and a CypA homology domain (Knockenhauer and Schwartz, 2016). It is the major component of the filaments on the cytoplasmic face of the NPC and has been implicated in a number of cellular activities other than nuclear import (Chatel and Fahrenkrog, 2012). While RANBP2 has previously been reported to enhance HIV-1 infection, the domains required and the mechanism by which RANBP2 facilitates HIV-1 infection remain controversial (Dharan et al., 2016; Di Nunzio et al., 2012; Meehan et al., 2014; Schaller et al., 2011; Zhang et al., 2010)(reviewed in (Matreyek and Engelman, 2013). In agreement with previous reports, we found that RANBP2 knockdown inhibited HIV-1_WT_ infection of HeLa cells. In HT1080 and HOS cells however, effects of RANBP2 knockdown on HIV-1_WT_ were more modest (3-fold) (Figure 8 and Figure 8—figure supplement 1).

RANBP2 depletion caused MX2 mislocalization and strongly reduced or eliminated its antiviral activity in HeLa cells. Conversely, RANBP2 depletion in non-dividing HT1080 cells had the opposite effect and increased MX2 antiviral activity, similar to NUP155 depletion (Figure 8). Moreover, while HIV-1_G89V_ infection was slightly increased by MX2 in unmanipulated HT1080 cells, RANBP2 depletion caused HIV-1_G89V_ to be inhibited by MX2 (Figure 9F).

RANBP2 depletion changed some, but not all, of the effects of CypA on HIV-1 infection. Similar to previous findings (Schaller et al., 2011), we found that RANBP2 depletion caused HIV-1_WT_ to become CsA-dependent in HeLa cells. RANBP2 depletion also eliminated the modest (three-fold) reduction in HIV-1_WT_ infection of CsA-treated HT1080 cells (Figure 10). Conversely, the enhancing effects of CsA addition on HIV-1_A92E_ infectivity in HeLa cells, and the profound inhibitory effect of CsA on HIV-1_N57S_ infection in HT1080 cells were maintained when RANBP2 was depleted (Figure 11). Thus, the effects of CsA addition in these contexts are not mediated by the cyclophilin homology domain of RANBP2 (Schaller et al., 2011). Furthermore, the deleterious effects of CsA on the antiviral activity of MX2, and the ability of MX2 to rescue HIV-1_N57S_ from inhibition by CsA in HT1080 cells were maintained in RANBP2 depleted cells (Figure 11C–D).

A recent report has also implicated RANBP2 in stabilization of the structure of the cytoplasmic ring of the Nup107 (Y) complex, adding an essential scaffolding function to this protein, which thereby affects the local conformation of the scaffold of the pore (von Appen et al., 2015). As a dynamic interactor with the NPC, it is then conceivable that in addition to direct interactions with the viral CA, RANBP2-dependent conformational changes create heterogeneity in NPCs that influence the interaction between the viral CA, MX2, and Nups.

### NUP88 and NUP214

NUP88 is localized to the cytoplasmic face of the NPC and forms a stable, interdependent subcomplex with NUP214 (Fornerod et al., 1997). Accordingly, depletion of either NUP88 or NUP214 dramatically reduced levels of NUP88 and NUP214, as measured by western blotting or immunofluorescence (Figure 5 and Figure 6—figure supplement 9 ) NUP88 and NUP214 have been reported to mediate attachment of RANBP2 to the NPC (Bernad et al., 2004). However, no mislocalization of RANBP2 or any of other Nups tested was observed upon NUP88 or NUP214 depletion (Figure 6—figure supplements 1–14). Nevertheless, perturbation of RANBP2-NUP88 interactions could potentially affect the overall configuration of nuclear pores.

Effects of NUP88 and NUP214 depletion on HIV-1 infection and MX2 activity were generally similar. Accordingly, NUP88 and NUP214 depletion reduced HIV-1, HIV-2, and especially SIVmac infection in dividing HeLa cells. Conversely these depletions did not affect HIV-1 infection of non-dividing HeLa cells, nor infection by HIV-1 of HT1080 or HOS cells or infection by nonprimate lentiviruses (Figure 8 and Figure 8—figure supplement 1; Figure 8—figure supplement 4; Figure 8—figure supplement 5).

NUP88 and NUP214 were not required for the localization of MX2 at the NPC but depletion of these proteins reduced the antiviral activity of MX2 against HIV-1_WT_ in both HeLa and HT1080 cells, irrespective of the cell cycle (Figure 8). However, MX2 activity against HIV-2 or SIVmac was unaffected. In the case of HIV-1_G89V_, whose infection was inhibited by NUP88 and NUP214 depletion in HeLa cells, expression of MX2 completely restored infection (Figure 9E–F). Thus, altering nuclear pore composition via NUP88/214 depletion clearly modified the effect of MX2 on infection in a CA dependent manner, but had little effect on the action of CsA/CypA. While not required for HIV-1_WT_ infection, NUP88/214 could play a direct role, as NUP88 bound to CA tubes and was depleted from cell lysates following incubation with CA tubes.

### NUP153

NUP153 is one of three Nups that form the basket-like structure that extends from the NPC into the nuclear interior. NUP153 directly interacts with the Nup107 complex, and is essential both for NPC formation during interphase, and for the recruitment of several Nups upon mitotic exit (Burke and Ellenberg, 2002; Vollmer et al., 2015). NUP153 was unique among the Nups examined in that it remained localized to the nuclear envelope following depletion of any other Nup (Figure 6 and Figure 6—figure supplements 1–6). This finding is consistent with the notion that NUP153 is the first Nup recruited to the chromosomes during late anaphase (Bodoor et al., 1999), and directly recruited to the inner nuclear membrane during interphase (Vollmer et al., 2015). This lack of dependence on other Nups further suggests that NUP153 helps to initiate the formation of nuclear pores. In addition to interactions with the NPC scaffold, NUP153 directly interacts with Ran, as well as with transport receptors, including TNPO1 (Nakielny et al., 1999). NUP153 may therefore facilitate nuclear import cycles by accelerating the dissociation of cargo-transport receptor complexes. Interestingly, NUP153 interaction with the NPC is dynamic; it shuttles and/or extends between the nuclear and cytoplasmic faces of the NPC (Nakielny et al., 1999) and is only transiently associated with a given NPC (Rabut et al., 2004b).

Like RANBP2, NUP153 has been reported to specifically enhance HIV-1 infection. Indeed, the C-terminal FG-rich region of NUP153 binds to HIV-1 CA, and has also been reported to bind integrase (Di Nunzio et al., 2013; Matreyek et al., 2013; Woodward et al., 2009). NUP153 depletion has also been shown to perturb integration site selection (Koh et al., 2013; Marini et al., 2015). NUP153 depletion profoundly perturbed the localization of each of the Nups tested and caused reductions in the overall levels of some Nups, presumably due to its key role in initiating nuclear pore formation. It was surprising, therefore, that while NUP153 depletion reduced HIV-1_WT_ infection of HeLa and HOS cells, the effect of NUP153 depletion on HIV-1_WT_ infection of HT1080 cells was marginal (Figure 8 and Figure 8—figure supplement 1). Mutations in HIV-1 CA had variously altered effects of NUP153 depletion on infection; most strikingly, HIV-1_G89V_ infection was unaffected by NUP153 depletion. Similarly, EIAV and FIV were unaffected by NUP153 depletion (Figure 8—figure supplement 5 and Figure 9).

Although NUP153 depletion caused MX2 mislocalization in both HT1080 and HeLa cells, MX2 antiviral activity was only reduced by NUP153 depletion in HeLa cells. In fact, in common with NUP155 and RANBP2 depletion, NUP153 depletion increased MX2 activity in growth arrested HT1080 cells. Thus knockdown of three different Nups caused HT1080 cells to mimic the phenotype of HeLa, HOS, and K562 cells, in the sense that MX2 activity was enhanced by growth arrest (Figure 1, Figure 8, and (Kane et al., 2013)).

The effects of NUP153 depletion on HIV-1 infection in CsA treated cells were also similar to the effects of RANBP2 depletion, rendering HIV-1_WT_ infection CsA dependent (as previously reported (Matreyek and Engelman, 2011; Schaller et al., 2011)) and exaggerating the CsA dependence of HIV-1_A94E_ in HeLa cells. Conversely, NUP153 depletion had only a minor effect on the moderate CsA sensitivity of HIV-1_WT_ or the extreme CsA sensitivity exhibited by HIV-1_N57S_ in HT1080 cells. Moreover, the ability of MX2 to rescue HIV-1_N57S_ from inhibition by CsA in HT1080 cells was unaffected by NUP153 depletion (Figures 10 and 11).

The increased antiviral activity of MX2 upon depletion of some Nups, specifically in non-dividing HT1080 cells, along with the importance of NUP153 in initiating interphase NPC formation suggest the possibility that some depletions preferentially deplete nascent pores. Existing pores, while smaller in number, may retain function but may be more readily occupied by MX2. Likewise, perhaps differences in the fraction of NPCs that are assembled in interphase could underlie the cell-type dependent differences in MX2 activity observed in in non-dividing cells.

### CPSF6 and TNPO3

TNPO3 is a nuclear import factor for serine-arginine-rich (SR) proteins involved in RNA splicing (Lai et al., 2001). TNPO3 was identified as a potential HIV-1 cofactor in genome-wide RNAi screens, and then later reported to directly bind the viral integrase and CA in vitro (reviewed in (Matreyek and Engelman, 2013). More recent studies indicate that the effect of TNPO3 depletion on HIV-1 infection is mediated by redistribution of the SR protein CPSF6 from the nucleus to the cytoplasm (De Iaco et al., 2013; Fricke et al., 2013). CPSF6 directly interacts with the viral CA via the same binding pocket as NUP153 (Lee et al., 2010; Matreyek et al., 2013; Price et al., 2012), and when re-localized to the cytoplasm, inhibits HIV-1 infection (Lee et al., 2010). Although CPSF6 depletion does not significantly affect HIV-1_WT_ infection in vitro (Lee et al., 2010), selective pressure appears to maintain CPSF6-CA interactions in vivo (Henning et al., 2014; Saito et al., 2016). Several potential explanations for maintenance of CPSF6-CA interactions have been proposed, including promoting interactions with other host factors such as NUP153 and RANBP2 (Henning et al., 2014; Price et al., 2012), evasion of cytosolic immune sensors (Rasaiyaah et al., 2013), and the targeting of integration to transcriptionally active chromatin (Rasheedi et al., 2016; Sowd et al., 2016).

We observed several CA-dependent, cell cycle-dependent, and cell-type dependent differences in sensitivity to TNPO3 and/or CPSF6 depletion that suggested additional cellular factors may influence their effect on HIV-1 infection. For example, CPSF6 depletion marginally reduced the anti-viral activity of MX2 in HeLa, but not HT1080 cells, irrespective of cell division (Figure 8). Conversely, while TNPO3 depletion reduced antiviral activity of MX2 in dividing HeLa and HOS cells, TNPO3 depletion enhanced MX2 antiviral activity in non-dividing cells of both types (Figure 8 and Figure 8—figure supplement 1). The CPSF6 binding interface is also conserved in HIV-2 and SIVmac (Price et al., 2012), and we found that these viruses were particularly sensitive (more so than HIV-1) to TNPO3 depletion (Figure 8—figure supplement 4), further supporting the importance of this interaction for primate lentivirus infection.

While CPSF6 depletion has little effect on WT HIV-1 infection in these in vitro assays, infection by HIV-1_T210K_ and HIV-1_G89V_ was increased by CPSF6 depletion, in a cell-type specific manner. Conversely, HIV-1_N57S_ infection was inhibited by either TNPO3 or CPSF6 knockdown, only in HOS cells (Figure 9 and Figure 9—figure supplement 1). Notably, CPSF6 depletion modified the effect of CsA in a cell type dependent manner. CPSF6 depletion caused HIV-1_WT_ to be marginally more infectious in HT1080 cells and completely resistant to inhibition by CsA (Figure 10B). Moreover, the CsA-dependence of HIV-1_A92E_ in HeLa cells and CsA-sensitivity of HIV-1_N57S_ in HT1080 cells was markedly reduced by CPSF6 knockdown. Curiously, the effects of CPSF6 depletion were reminiscent of the effects of NUP155 depletion, in that they similarly reduced the respective CsA-induced enhancement or inhibition of HIV-1_WT_, HIV-1_A92E_, and HIV-1_N57S_ infection (Figures 10 and 11). However, the effects of NUP155 were not the result of changes in CPSF6 localization, which was unaltered by NUP155 knockdown.

The effects of CPSF6 on HIV-1_N57S_ mutant were surprising given that studies indicate that N57 is critical for interaction with CPSF6 (Matreyek et al., 2013; Price et al., 2012). Furthermore, since NUP153 binds CA in this pocket, it was expected that HIV-1_N57S_ would be NUP153-independent, as has been shown for HIV-1_N57A_ in HOS cells (Matreyek et al., 2013). However, while HIV-1_N57S_ infection was indeed largely insensitive to NUP153 depletion in HOS and HT1080 cells, HIV-1_WT_ and HIV-1_N57S_ were equivalently sensitive to NUP153 knockdown in HeLa cells (Figure 8A, Figure 8—figure supplement 1, Figure 9, and Figure 9—figure supplement 1). Collectively, these data suggest that NUP153 or CPSF6 might have roles in HIV-1 infection beyond their interactions with their binding pocket in CA.

### KPNB1

Depletion of KPNB1 had pleitropic effects in the levels of other Nups, and also appeared to cause growth arrest (Figure 5—figure supplements 7 and 8). Despite the pleiotropic effects, KPNB1 depletion only moderately inhibited HIV-1 infection in HeLa cells, in a manner that was accentuated by aphidicolin treatment. KPNB1 depletion had a smaller effect on HIV-1_WT_ infection in HT1080 cells but caused a large accentuation of MX2 activity. Indeed HIV-1 infection was inhibited by ~200 fold in non-dividing HT1080 cells depleted of KPNB1. Notably, depletion of KPNB1 slightly sensitized EIAV and FIV to MX2. HIV-1_N57S_ was strikingly inhibited by KPNB1 depletion, and the ability of MX2 to reverse CypA induced inhibition of HIV-1_N57S_ infection was abolished by KPNB1 depletion (Figure 8, Figure 8—figure supplement 5, Figure 9A, Figure 9—figure supplement 1B–C, and Figure 11C). KPNB1 has been shown to interact with NUPL1 (NUP58) and NUP153, thereby affecting the conformation of the Nup62 complex in the central channel and altering the permeability of the pore in a Ran-dependent manner (Koh and Blobel, 2015b; Lowe et al., 2015). These reports in combination with our findings suggest the possibility that depletion of KPNB1 could alter the structure of the NPC in a cell-type dependent manner.

Exploration of the utilization of cellular machinery by viruses and the mechanisms by which cells attempt to directly inhibit viral replication has historically been of great value in revealing not only the molecular details of viral replication, but also the normal workings of the cell. Our data highlight the complexity of the interaction between HIV-1, the NPC, and other cellular proteins that interact with the HIV-1 CA and reveal heterogeneity in nucleocytoplasmic trafficking both within and between cells that influences viral infection and susceptibility to an innate immune effector. These findings should inform future efforts to uncover the precise nature of NPC heterogeneity and the interaction between HIV-1 and cellular proteins involved in viral nuclear entry.

## Materials and methods

### Plasmid construction

The LKO-derived tetracycline-inducible lentiviral expression vector was modified from the previously described pLKO.dCMV.TetO/R (pLKOΔ) (Busnadiego et al., 2014). The unique *Not*I site in pLKOΔ-MycHsMX2-IP was destroyed and the myc-tag and MX2 ORF were removed and replaced with an *Sfi*I*-EcoR*I*-Not*I*-Sfi*I multiple cloning site by oligonucleotide annealing using *Nhe*I *and E*co*R*V sites. pLKOΔ-blasti was constructed by transfer of a unique *Xma*I/*Not*I fragment (containing sequences for the internal ribosome entry site [IRES] and blasticidin-resistance cassette) from CSIB (Kane et al., 2013) into pLKOΔ replacing the IRES and puromycin-resistance cassette. Untagged MX2 was then inserted into pLKOΔ vector using *Sfi*I.

Doxycycline-inducible expression vector containing an MX2-tagRFP (pLKOΔ) fusion protein was generated by PCR amplification of HsMX2 with the following primers: MX2 (from pCSIB-MX2 (Kane et al., 2013)) 5’-CTC TGG CCG AGA GGG CCA TGT CTA AGG CCC ACA AGC CTT G-3’ and 5’-ATA AAG AAT GCG GCC GCC GTG GAT CTC TTT GCT GGA GAA-3’; tagRFP (from pLKOΔ-MyctagRFP (Busnadiego et al., 2014)) 5’-ATA AGA GCG GCC GCC ATG AGC GAG CTG ATT AAG GAG AA-3’ and 5’-CTC TGG CCA GAG AGG CCT CAC TTC TGC CCC AGT TTG CTA G-3’ using *Sfi*I and *Not*I sites. pLKOΔ-blasti containing tagRFP with directional in-frame *Sfi*I sites upstream of the start codon were generated by PCR amplification with the following primers: tagRFP 5’-ATA AGA ATG CGG CCG CCG CTT GCT AGC CTC TGG CCG AGA GGG CCC TCT CTC TCT GGC CTC TCT GGC CAG AGC GAG CTG ATT AAG GA GAA CA-3’ and 5’-ATA AAG AAT GCG GCC GCC GTC TAG ATA TCT CAC TTG TGC CCC AGT TTG C-3’; and insertion using *Nhe*I and *EcoR*V sites. pLKOΔ-blasti expressing a CSPF6-tagRFP fusion protein was then generated by PCR amplification of CPSF6 (Open Biosystems MHS4771-99611074) with the following primers: 5’-CTC TGG CCA GAG AGG CAT GGC GGA CGG CGT GGA C-3’ and 5’-CTC TGG CCA GAG AGG CCA CGA TGA CGA TAT TCG CG-3’ and inserted using *Sfi*I.

pLKOΔ containing C-terminally myc tagged ARFAPTIN2 with directional in-frame *Sfi*I sites upstream of the start codon was generated by PCR amplification of ARFIP2 (from pLX304-ARFIP2 (Yang et al., 2011)) with the following primers: 5’-AAG CTT GCT AGC ACC TGG GCC GAG AGG GCC GTT AAC GTC GAC GGC CTC TCT GGC CTG ACG GAC GGG ATC CTA GGG-3’ and 5’-ATT CTA GAT ATC GAC TCA CAG ATC CTC TTC AGA GAT GAG TTT CTG CTC CTG CTC CTC TAG CCA GGA GG-3’ using *Nhe*I and *EcoR*V sites. N-terminal fusions containing CypA [from *Macacca mulatta* TRIMCyp], the N-terminal 43 amino acids of Mx1, and the N-terminal amino acids of MX2 were generated using *Sfi*I sites following PCR amplification with the following primers: CypA 5’-CTC TGG CCG AGA GGG CCA TGG TCA ATC CTA CTG TGT TCT TCG-3’ and 5’-CTC TGG CCA GAG AGG CCT TCG AGT TGT CCA CAG TCA GC-3’; Mx1N43 5’-CTC TGG CCG AGA GGG CCA TGG TTG TTT CCG AAG TGG AC-3’ and 5’-CTC TGG CCG AGA GGG CCG CTG CAC AGG TTG TTC TCA GC-3’; MX2N91 5’-CTC TGG CCG AGA GGG CCA TGT CTA AGG CCC ACA AGC CTT G-3’ and 3’- CTC TGG CCA GAG AGG CCG CTG TAC AGG TTG TTC TCG GG-3’.

NLS-GFP-LacZ fusions were expressed using an LHCX based retroviral vector (Invitrogen). A GFP-LacZ fusion containing a multiple cloning site immediately following the start codon was generated by PCR amplification with the following primers: eGFP 5’-ATA AGA ATA AGC TTC CAC CAA TGG GCC GAG AGG GCC GAA GCC ATT CCT CGA GGG CCG AGA GGG CCG TGA GCA AGG GCG AGG AGC TG-3’ and 5’-ATA AGA ATG GCG GCC GCC TTG TAC AGC TCG TCC ATG CC-3’; LacZ 5’-ATA AGA ATG CGG CCG GGC GTC GTT TTA CAA CGT CGT GAC T-3’ and 5’-ATA AGA ATG TTA ACT TAT TTT TGA CAC CAG ACC AAC T-3’ and inserted using *Hind*III, *Not*I, and *Hpa*I. NLS sequences were then inserted via oligonucleotide annealing or PCR amplification and insertion using *Sfi*I as outlined in Table 1.

An HIV-1 GFP reporter virus containing the N74D capsid mutation (HIV-1_N74D_) was generated by overlap PCR and insertion into pNHGCapNM as described previously (Rihn et al., 2013) using *Not*I and *Mlu*I sites with the following primers: CapNM 5’-GTA AGA AAA AGG CAC AGC AAG CGG CCG CTG-3’ and 5’-CTT GGC TCA TTG CTT CAG CCA AAA CGC GTG-3’; N74D 5’-GTT AAA AGA GAC CAT CGA TGA GGA AGC TGC AG-3’ and 5’-CTG CAG CTT CCT CAT CGA TGG TCT CTT TTA AC-3’.

### Cell lines

The adherent human HEK 293T (CVCL_0063), HeLa (CVCL_0030)HOS (CVCL_0312), and HT1080 (CVCL_0317) cell lines were maintained in Dulbecco’s Modified Eagles Medium (DMEM) with 10% fetal calf serum (FCS) and gentamicin. The suspension T cell [MT4 (CVCL_2632), CEM X174 (CVCL_X615), SupT1(CVCL_1714), Jurkat (CVCL_0367), H9(CVCL_1240)] and monocytic cell [THP-1 (CVCL_0006), K-562 (CVCL_0004), U937 (CVCL_0007)] lines were maintained in Roswell Park Memorial Institute Medium (RPMI) with 10% FCS and gentamicin. All cells were purchased from ATCC or provided by the NIH AIDS Reagent Program (MT4 and CEM X174) and were assumed to authenticated by their supplier and were not further characterized. Cells were monitored for retroviral contamination by SYBR-Green based PCR RT assay. Mycoplasma testing was not specifically performed, but many cell lines were used in immunofluorescence assays with Hoechst staining that would have revealed the presence of mycoplasma. Derivatives of HeLa and HT1080 cells containing doxycycline-inducible MX2, or fusion proteins were generated by transduction with LKO-derived lentiviral vectors (Busnadiego et al., 2014) followed by selection in 1 μg ml^−1^ puromycin or 5 μg ml^−1^ blasticidin (Sigma-Aldrich). Vector stocks for transduction were generated by co-transfection of 293 T cells with a VSV-G expression plasmid, an HIV-1_NL4-3_ Gag-Pol expression plasmid, and a CSIB or LKO-derived vector, or an MLV Gag-Pol expression plasmid and an LHCX-derived vector using polyethyleneimine (PolySciences). Expression was induced in pLKO transduced cell lines through an overnight treatment with 500 ng/ml doxycycline hyclate (Sigma-Aldrich) prior to challenge with retroviruses or retroviral vectors.

### Primary cell isolation

Human peripheral blood mononuclear cells were isolated from blood by gradient centrifugation in Lymphocyte Separation Medium (Corning). For macrophage isolation, 10^8^ cells/dish were plated in 10 cm dishes in serum free RPMI (Gibco) for four hours, followed by aspiration to remove non-adherent cells and replacement of medium with 10% FCS supplemented with 100 ng/ml GMCSF (Gibco) for 72 hr (media was replaced once at 24 hr). For CD4^+^ T cell isolation, 3 × 10^6^ cell/mls were grown in RPMI medium supplemented with 10% FCS and 50 u/ml IL-2 (PreproTech) for 72 hr followed by purification with the Human CD4^+^ T cell Enrichment Kit (Stem Cell Technologies). Cells were lysed in NuPage LDS buffer (Novex), and the appropriate dilutions for western blotting were determined by comparison of LAMIN B1 levels between MT4, THP-1, and primary cell lysates.

### Viruses

All viruses were generated by transfection of 293 T cells using using polyethyleneimine (PolySciences). GFP reporter proviral plasmids HIV-1_NL4-3_ΔEnv-GFP (HIV-1, HIV-1_G89V_, HIV-1_A92E_, and G94D CA mutants (Hatziioannou et al., 2004)), NHGCapNM (wild-type, HIV-1_N57S_, HIV-1_N74D_, G208R, and HIV-1_T210K_ CA mutants (this investigation and (Rihn et al., 2013)), HIV-2_ROD_ΔEnv-GFP, SIV_MAC_ΔEnv-GFP (Hatziioannou et al., 2003) 10 μg of proviral plasmid was co-transfected with 1 μg of VSV-G expression plasmid. For MLV, EIAV, and FIV, three plasmid vector systems (Kemler et al., 2002; Mitrophanous et al., 1999; Neil et al., 2001; Soneoka et al., 1995) were also used to generate GFP reporter viruses, whereby 5 μg of Gag-Pol, 5 μg of packeagable genome, and 1 μg of VSV-G expression plasmids were co-transfected. Levels of reverse transcriptase in viral stocks were quantified using a one-step SYBR-Green based PCR RT assay as previously described (Del Prete et al., 2017; Pizzato et al., 2009).

### Infection assays

Infectivity was measured in HOS, HeLa, or HT1080 cells seeded in 96-well plates at 5 × 10^3^ cells per well and inoculated with serial-dilutions of VSV-G pseudotyped GFP reporter viruses in the presence of 4 μg ml^−1^ polybrene (Sigma-Aldrich). Two days post-infection, cells were trypsinized and fixed in 2% paraformaldehyde. For experiments in which infection of dividing and non-dividing cells was compared, cells were treated with 1 μg ml^−1^aphidicolin (Sigma-Aldrich) for 16 hr before infection. Where indicated, cyclosporine A (Sandoz) was added to the cultures at the time of infection at 5 μM. Infected cells (%GFP positive of viable cells) were enumerated by FACS analysis using a CyFlow cytometer (Partec) coupled to a Hypercyte Autosampler (Intellicyt).

### RNA interference

HOS, HeLa, or HT1080 cells were reverse transfected with 25 pmol of siRNA (Table 2; SMARTpool, Dharmacon) using Lipofectamine RNAiMax (Invitrogen) at a concentration of 3 × 10^4^ cells/ml in 6- or 12-well plates. Non-transfected cells, transfection reagent alone, and non-targeting siRNA were used as controls, no significant difference in viral infectivity or MX2-restriction was observed for each of these controls, as such, for each experiment, only the non-targeting siRNA control is shown. 48 hr after transfection, cells were trypsinized, diluted 1:2.5 and re-plated in 24- or 96-well plates and treated with doxycycline and/or aphidicolin followed by infection with GFP reporter viruses, fixation for immunofluorescence, or lysis for western blotting 16 hr later (See Figure 4A).

**Table 2.** ON-TARGET SMARTpool siRNA utilized in this investigation

Gene symbol	Gene ID
*AAAS*	8086
*AHCTF1*	25909
*CPSF6*	11052
*GLE1*	2733
*IPO11*	51194
*IPO13*	9670
*IPO4*	79711
*IPO5*	3843
*IPO7*	10527
*IPO8*	10526
*IPO9*	55705
*KPNA1*	3836
*KPNA2*	3838
*KPNA3*	3839
*KPNA4*	3840
*KPNA5*	3841
*KPNA6*	23633
*KPNB1*	3837
*MX2*	4600
*NDC1*	55706
*NUP107*	57122
*NUP133*	55746
*NUP153*	9972
*NUP155*	9631
*NUP160*	23279
*NUP188*	23511
*NUP205*	23165
*NUP210*	23225
*NUP214*	8021
*NUP35*	129401
*NUP37*	79023
*NUP43*	348995
*NUP50*	10762
*NUP54*	53371
*NUP62*	23636
*NUP85*	79902
*NUP88*	4927
*NUP93*	9688
*NUP98*	4928
*NUPL1*	9818
*POM121*	9883
*RAE1*	8480
*RANBP2*	5903
*SEC13*	6396
*SEH1L*	81929
*TNPO1*	3842
*TNPO2*	30000
*TNPO3*	23534
*TPR*	7175

### Western blotting

Cell suspensions were lysed in NuPage LDS (Novex) or SDS sample buffer, followed by sonication, and separated by electrophoresis on NuPage 4–12% Bis-Tris gels or 3–8% Tris-Acetate gels (Novex) and blotted onto polyvinylidene fluoride (PDVF, BioRad Laboratories) or nitrocellulose (GE Healthcare). Membranes were incubated with the antibodies listed in Table 3, followed by incubation with goat anti-rabbit-HRP or goat anti-mouse-HRP secondary antibodies (Jackson ImmunoResearch) or goat-anti-mouse IRDye680RD or IRDye800CW (LI-COR Biosciences). SeeBlue and HiMark Pre-stained Protein Standards (Thermo Fisher) were used for Bis-Tris and Tris-Acetate gels respectively. Blots were developed with SuperSignal West Femto Maximum Sensitivity Substrate (Thermo Scientific) and imaged/quantified on a C-Digit or LI-COR Odyssey scanner (LI-COR Biosciences).

**Table 3.** Antibodies utilized in this investigation

**Reactivity**	**Species**	**Company**	**Catalog number**
ALADIN	rabbit	Novus Biologicals	NBP2-21596
CPSF6	rabbit	ProteinTech	15489–1-AP
CPSF6	rabbit	Abcam	ab175237
ELYS	mouse	Abcam	ab53540
GAPDH	mouse	Santa Cruz	sc-32233
KPNA1	rabbit	ProteinTech	18137–1-AP
KPNA2	rabbit	Abcam	ab84440
LAMIN B1	rabbit	Abcam	ab133741
MX2	rabbit	Novus Biologicals	NBP1-8108
MYCtag	mouse	Millipore	05–724
NDC1	rabbit	Novus Biologicals	NBP1-91603
NUP107	rabbit	Abcam	ab73290
NUP133	mouse	Santa Cruz	376763
NUP153	mouse	Abcam	ab24700
NUP155	rabbit	Abcam	ab157104
NUP160	rabbit	Abcam	ab74147
NUP188	rabbit	Abcam	ab86601
NUP205	rabbit	Abcam	ab157090
NUP210	rabbit	Novus Biologicals	NB100-93336
NUP214	rabbit	Bethyl Labs	IHC-00103
NUP35	rabbit	Bethyl Labs	A301-781A
NUP37	rabbit	Abcam	ab201161
NUP43	rabbit	Bethyl Labs	A303-976A
NUP50	rabbit	Bethyl Labs	A301-783A
NUP54	rabbit	ProteinTech	16232–1 AP
NUP62	mouse	BD Biosciences	610498
NUP62	mouse FITC-conjugated	BD Biosciences	611962
NUP85	mouse	Santa Cruz	376111
NUP88	mouse	BD Biosciences	611896
NUP93	mouse	Abcam	ab53750
NUP98	rabbit	Cell Signaling	C39A3
NUPL1	rabbit	ProteinTech	19907–1-AP
HIV-1 p24	mouse	NIH AIDS Reagent Program	182-H12-5C
POM121	rabbit	Abcam	ab190015
POM121	rabbit	ProteinTech	15645–1-AP
RAE1	rabbit	Abcam	ab124783
RANBP2	rabbit	Abcam	ab64276
SEC13	rabbit	ProteinTech	15397
TNPO1	mouse	Sigma-Aldrich	WH0003842M1
TNPO3	mouse	Abcam	ab54353
TPR	rabbit	Abcam	wh000717m1
TUBULIN	mouse	Sigma-Aldrich	T6074

### Cell-cycle profile analysis

Cell-cycle profiles of HeLa and HOS cells were determined 64 hr post-siRNA transfection, or 16 hr post aphidicolin treatment. HT1080 cells were not amenable to reproducible DNA content staining and were not included in this assay. Cells were trypsinized and fixed in ice cold 70% ethanol followed by staining with FxCycle PI/RNase Staining Solution (Thermo Scientific) or 20 μM DRAQ5 Fluorescent Probe Solution (Thermo Scientific). DNA content was then determined by FACS analysis using an Attune cytometer (Thermo Scientfic) coupled to an autosampler.

### Microscopy

HeLa or HT1080 cells stably transduced with doxycycline-inducible expression vectors [MX2-tagRFP, CPSF6-tagRFP, ARFAPTIN2-myc, or MX2(N91)-ARFAPTIN2-myc] were seeded onto 24-well gelatin-coated glass-bottomed dishes and treated with 500 ng/ml doxycycline 16 hr prior to fixation with 4% paraformaldehyde or −20°C methanol. Cells were then permeabilized with 0.5%Triton X-100 (and 0.05%SDS in some cases) (Thermo Scientific) and immunostained with the indicated antibodies followed by goat anti-mouse or goat anti-rabbit Alexa 488 or Alexa 647 secondary antibodies (Molecular Probes). DNA was stained with Hoescht 33342 (Thermo Scientific). Cells were visualized by deconvolution microscopy as described previously (Jouvenet et al., 2006) or on an EVOS digital microscope [NLS-GFP-LacZ fusions only (Electron Microscopy Sciences)]. Image generation and co-localization analysis were completed with the Imaris software suite (Bitplate). Pearson’s coefficient values were derived by analysis of 3-dimensional image reconstructions of the nuclear surface for 8–12 individual cells.

### CA-binding assay with HIV-1 CA tubes

CA tubes were assembled as described (Cortines et al., 2011; Larue et al., 2012) by incubating the purified protein in a high-salt buffer (25 mM Tris-HCl, pH 8; 2M NaCl; 10 mM β-mercaptoethanol) for ~12 hr. HeLa cells were cultivated with or without 500 ng/µl doxycycline for a minimum of 16 hr. The cells were then lysed for pull-down experiments by adding passive lysis buffer (Promega) supplemented with protease inhibitor cocktail (Roche). NaCl concentration in lysates was adjusted to 2M and lysates were centrifuged at 13,000 x g for 2 min at 4°C. Then the supernatant of the cell lysates were added to the preformed CA tubes and incubated at 4°C for 2 hr. Following centrifugation at 13,000 x g for 2 min at 4°C, the supernatant was saved and the pellet was washed three times with the high-salt buffer. NuPage LDS Sample buffer (Thermo Fisher) supplemented with 50 mM DTT was added to all the samples including pulled-down and unbound fractions and subjected to SDS-PAGE. The proteins of interest were detected by immunoblotting using respective antibodies.

### Statistical analysis

Statistical significance was determined using Excel (two-tailed t test). Where appropriate (eg. comparison of doxycycline treated and untreated samples infected with the same virus dilution), a paired t test was utilized. For comparison of infectivity data in Nup and NTR siRNA transfected cells, viral titers were converted to a percentage of control siRNA transfected cells before application of statistical tests. For comparison of MX2 activity (or CsA effects), viral titers were converted to a fold change in MX2 activity (or CsA effect) as compared to control siRNA transfected cells. a complete set of statistical analyses of all quantitative data in the manuscript is given in Supplemental file 1

## Additional files

Supplementary File 1. Statistical analyses. Tab 1. Figure 1 statistics. P values generated from two-tailed paired t tests for data represented in Figure 1. Tab 2. Nup and NTR knockdown statistics 1. P values generated from two-tailed paired t tests for data represented in Figures 8–11, addressing significance within experiments. Tab 3. Nup and NTR knockdown statistics 2. P values generated from two-tailed unpaired t tests for data represented in Figures 8–11, addressing significance between experiments. Tab 4. Figure 8 additional statistics. P values generated from two-tailed paired t tests for data represented in Figure 8—figure supplements 2D and 3A–C.

Transparent reporting form

### Data availability

All data generated or analysed during this study are included in the manuscript and supporting files.
