# Supplementary material for: Nuclear pore heterogeneity influences HIV-1 infection and the antiviral activity of MX2
Source: eLife. 2018 Aug 7;7:e35738. doi: 10.7554/eLife.35738 (PMC6101944; doi:10.7554/eLife.35738)
Supplement: Transparent reporting form [file elife-35738-transrepform.docx]

***eLife’s* transparent reporting form**

We encourage authors to provide detailed information *within their submission* to facilitate the interpretation and replication of experiments. Authors can upload supporting documentation to indicate the use of appropriate reporting guidelines for health-related research (see [EQUATOR Network](http://www.equator-network.org/%20)), life science research (see the [BioSharing Information Resource](https://biosharing.org/)), or the [ARRIVE guidelines](http://www.plosbiology.org/article/info:doi/10.1371/journal.pbio.1000412) for reporting work involving animal research. Where applicable, authors should refer to any relevant reporting standards documents in this form.

If you have any questions, please consult our Journal Policies and/or contact us: [editorial@elifesciences.org](mailto:editorial@elifesciences.org).

**Sample-size estimation**

- You should state whether an appropriate sample size was computed when the study was being designed
- You should state the statistical method of sample size computation and any required assumptions
- If no explicit power analysis was used, you should describe how you decided what sample (replicate) size (number) to use

Please outline where this information can be found within the submission (e.g., sections or figure legends), or explain why this information doesn’t apply to your submission:

Sample sizes (specifically, numbers of cells utilized for each assay) are indicated in the materials and methods – in the sections headed “Infection assays” and “RNA interference”.

**Replicates**

- You should report how often each experiment was performed
- You should include a definition of biological versus technical replication
- The data obtained should be provided and sufficient information should be provided to indicate the number of independent biological and/or technical replicates
- If you encountered any outliers, you should describe how these were handled
- Criteria for exclusion/inclusion of data should be clearly stated
- High-throughput sequence data should be uploaded before submission, with a private link for reviewers provided (these are available from both GEO and ArrayExpress)

Please outline where this information can be found within the submission (e.g., sections or figure legends), or explain why this information doesn’t apply to your submission:

Number of times each experiment was performed is indicated in each figure legend. Number of technical replicates for each experiment is also indicated in figure legends. For infectivity assays, experiments were conducted on at least three independent occasions (biological replicates) and triplicates representing three dilutions of GFP-reporter virus within the linear range (technical replicates) are shown in each figure. For microscopy assays, experiments were conducted on at least two independent occasions (biological replicates), with multiple images taken for each experiment (technical replicates) and one representative image for each condition is shown.

**Statistical reporting**

- Statistical analysis methods should be described and justified
- Raw data should be presented in figures whenever informative to do so (typically when N per group is less than 10)
- For each experiment, you should identify the statistical tests used, exact values of N, definitions of center, methods of multiple test correction, and dispersion and precision measures (e.g., mean, median, SD, SEM, confidence intervals; and, for the major substantive results, a measure of effect size (e.g., Pearson's r, Cohen's d)
- Report exact p-values wherever possible alongside the summary statistics and 95% confidence intervals. These should be reported for all key questions and not only when the p-value is less than 0.05.

Please outline where this information can be found within the submission (e.g., sections or figure legends), or explain why this information doesn’t apply to your submission:

For infectivity assays, data are presented as a mean, with error bars indicating the SEM, and is indicated in each figure legend.

Statistical analysis for all infectivity assays is included as a supplemental file (Supplemental File 1) and were conducted as indicated in the materials and methods.

Figure 7 includes Pearson’s coefficients for co-localization of proteins examined by immunofluorescence as indicated in the figure legend, and the methodology used to determine the coefficient is described in the Materials and Methods.

(For large datasets, or papers with a very large number of statistical tests, you may upload a single table file with tests, Ns, etc., with reference to sections in the manuscript.)

**Group allocation**

- Indicate how samples were allocated into experimental groups (in the case of clinical studies, please specify allocation to treatment method); if randomization was used, please also state if restricted randomization was applied
- Indicate if masking was used during group allocation, data collection and/or data analysis

Please outline where this information can be found within the submission (e.g., sections or figure legends), or explain why this information doesn’t apply to your submission:

Experimental groups were determined by the virus (including various “wild-type”GFP reporter viruses, and capsid mutant viruses), cell type, and other conditions (ie. siRNA transfection, aphidicolin treatment, cyclosporine A addition, Mx2 expression, Arfaptin2 fusion protein expression) and is clearly indicated in the figures and figure legends.

**Additional data files (“source data”)**

- We encourage you to upload relevant additional data files, such as numerical data that are represented as a graph in a figure, or as a summary table
- Where provided, these should be in the most useful format, and they can be uploaded as “Source data” files linked to a main figure or table
- Include model definition files including the full list of parameters used
- Include code used for data analysis (e.g., R, MatLab)
- Avoid stating that data files are “available upon request”

Please indicate the figures or tables for which source data files have been provided:

Source data for microscopy analysis is included as Figure 6- figure supplements and Figure 7- figure supplements.

Source data for quantification of protein expression upon siRNA transfection is included as Figure 5- figure supplements 3 – 6.
