# Supplementary material for: Nuclear pore heterogeneity influences HIV-1 infection and the antiviral activity of MX2
Source: eLife. 2018 Aug 7;7:e35738. doi: 10.7554/eLife.35738 (PMC6101944; doi:10.7554/eLife.35738)
Supplement: Peer review history

# Decision letter

**DOI:** 10.7554/eLife.35738.064

**Affiliations:** Icahn School of Medicine at Mount SinaiUnited States

In the interests of transparency, eLife includes the editorial decision letter and accompanying author responses. A lightly edited version of the letter sent to the authors after peer review is shown, indicating the most substantive concerns; minor comments are not usually included.

Thank you for submitting your article "Nuclear pore heterogeneity influences HIV-1 infection and the antiviral activity of Mx2" for consideration by *eLife*. Your article has been reviewed by three peer reviewers, and the evaluation has been overseen by a Reviewing Editor and Wenhui Li as the Senior Editor. The following individuals involved in review of your submission have agreed to reveal their identity: Olivier Schwartz (Reviewer #3).

The reviewers have discussed the reviews with one another and the Reviewing Editor has drafted this decision to help you prepare a revised submission

Summary:

This paper by Bieniasz and colleagues represents a tremendous effort towards understanding the complex interplay between HIV-1, MxB, CypA, and the nuclear pore complex. All three reviewers commented on the exceptionally comprehensive nature of the work ("Herculean in terms of its informational content"), which will likely make it an instant "classic" after publication.

The work presented will, indeed, help explain many of the inconsistencies in the literature. It importantly attacks the issue of the nuclear pore complex (NPC) in HIV infection and Mx2 antiviral activity. It does a great job tying together different aspects of the literature amassed over the prior 10+ years in terms of CA, the NPC, and HIV infection.

The authors assess antiviral activity of Mx2 with WT HIV and numerous CA mutants as a function of cellular CypA as well as NUP and NTR content using three cell-line models (HeLa, HT1080, and HOS), which were chosen based on their sensitivity to siRNA.

The results presented indicate that the impact of Mx2 depends on the cell type, with interesting observations of Mx2 increasing HIV-1 infectivity in some conditions. It is further shown that HIV CA interacts with several nucleoporins. The authors also studied the impact of Nups knockdown on nuclear import of non-viral cargos and on HIV-1 infectivity. It is shown that the effects of the knockdown a given Nup are difficult to interpret, since this silencing may also dysregulate many other Nups. The effects of Nups depletion on HIV infectivity depend on the cell type, the cell cycle, or the presence of CsA. Therefore, HIV may use different nuclear pore complexes composed of different sets of Nups to access the nucleus. These different NPCs vary in different cell types and are differently targeted by Mx2. Experiments to assess whether Mx2 inhibits nuclear location of artificial GFP-lacZ constructs engineered to harbor heterologous NLS sequences are also included. Several different lentiviruses and retroviruses are used in the experiments.

Taken together, this manuscript provides a comprehensive framework that reveals the previously unappreciated importance and variability of nuclear pores on retroviral infection.

Essential revisions:

The reviewers recognize that this is already a very complex study but feel that some additional clarifications would strengthen the overall conclusions. These requests are divided into the following main categories:

1) Improve the readability of the manuscript:

- There are numerous western panels, which authors unfortunately present out of context, at many times just slices of immunoblots. This reporting style is unacceptable at many journals. Minimally, positions of migration standards should be shown alongside all panels.

- Because of the sheer amount of data presented in the figures, the readers will benefit from some schematic guide. The presentation style in Figure 4, specifically the diagram of the nuclear pore, is greatly appreciated. A smaller version of this nuclear pore in each relevant figure would help (like was done with Figure 6—figure supplement 1). More substantial figure legends and perhaps summary tables may also help orient readers and parse all of the information.

2) Address the following considerations regarding the CA binding/pelleting assay used:

- This is not a novel assay but rather a standard Capsid binding assay conducted in physiologically irrelevant high salt (2M) concentration. Please re-phrase.

- The high-salt condition (2M NaCl) may actually have unforeseen consequences on which proteins are seen binding to CA. It could be considered a very stringent assay in terms of electrostatic interactions. In fact, it is somewhat surprising that MxB co-pelleted under this condition, as the interaction likely has a strong electrostatic nature (it is known that the charged RRR at MxB N-terminus is important for binding). This may hint the MxB-capsid interaction is greatly enhanced either by avidity effect (oligomerization) or by very high local concentration when fixed at the NPC.

- However, very high salt could enhance artifactual hydrophobic protein interactions, especially considering the inherent hydrophobicity of FG repeat containing proteins. Others who previously assessed binding of NUP153, e.g., to CA-based nanotubes performed assays under physiologically relevant salt (e.g., PBS), and the binding results here in 2M salt with different Nups do not always correlate with HIV-1 infection profile. Have authors tested binding in PBS? If so, how do these results compare to binding at high salt?

- Minimally, the potential for high salt to enhance binding artifacts should be discussed.

3) Include cell viability controls under different knockdown conditions (optimally, growth rate):

- One expects that depletion of some (several?) of the targeted factors to grossly effect cell physiology, which could be assessed by cell growth rate, viability, cell cycle status, etc. Although authors do a fair amount of controls, including impressive cross-monitoring of all NUPs upon single NUP depletion, they provide basically no data assessing cell fitness outside of virus infection. In subsection “KPNB1” they state KPNB1 knockdown induced growth arrest, so it sounds like they have some of the relevant data. I realize at 35 (many multi-panel) figures we may already be looking at a record, but I nevertheless think it is critical to document impacts on cell physiology caused by the knockdowns.

4) Provide additional information regarding Mx2 experiments:

- Please show Mx2 induction in the different engineered cell lines by western blot.

- Also clarify the statement regarding MxB "enhancement" of HIV infectivity, since MxB expression is returning infectivity to the wild-type level. It only modestly rescues gross infectivity defects in certain CA mutational backgrounds, which is different from promoting infection.

5) Provide some evidence for whether or not primary human cells resemble any of the cell types tested in terms of Nups composition:

- It would have been useful to validate, at least, some of the results in HIV-1-infected primary cells to increase the relevance of the findings. Detailed protein expression profiles of primary human T cells or macrophages from healthy donors would add significance to the detailed NUP profiles reported for the cell lines.

6) Strengthen the statistic components of the manuscript:

- The number of experimental replicates across the board must be clarified. Although authors tabulate number of experimental repeats and variance (error bars) for infection experiments, one shortcoming is that they do not analyze samples cross wise to reveal which of the documented changes are significant (generally assessed as P < 0.05). Though generally important and required for publication, statistics really apply here as authors highlight differential effects of certain NUP depletions on virus infection in different cell types. As but one example in the Results, they highlight comparatively marginal role for RANBP2 in HT1080 and HOS cells as compared to HeLa cells. Are the requirements for RANBP2 in HT1018 and HOS significant or not? This is but one example; paper would be best served if all results are analyzed for significance, and resulting p values reported.

- p value analyses must be included for the innumerable comparisons that they make in text. Minimally, all text callouts to differences between samples, large or small, should be accompanied with stated p value.

- The Authors indicate in legends the number of repeats for the infection experiments, though generally fail to note numbers of times results were seen for other experimental approaches. Please also add this info to legends. In the manuscript heatmaps are used to summarize western blot results, which presumably analyzed several independent westerns. In such cases, indicate number of replicates behind the heatmaps, and that (one assumes) the associated blotting panels show a representative replicate.

# Reply: Author response

**DOI:** 10.7554/eLife.35738.065

We appreciate the positive feedback and constructive comments of the reviewers, as well as the time and effort required to review this long manuscript. We have addressed each individual reviewer comment below and revised the manuscript accordingly.

Among the salient modifications the manuscript is the inclusion of the following new figures/supplements:

· Statistical analyses for all experiments involving viral infectivity (Supplementary file 1)

· Western blot analysis showing Mx2 expression (Figure 1—figure supplement 1B)

· Western blot analysis showing Nup and NTR expression in primary human CD4^+^ T cells and macrophages (Figure 2—figure supplements 2 and 3)

· CA binding assay with HIV-1_N74D_ (Figure 3—figure supplement 1B)

· Cell-cycle profiles following Nup/NTR siRNA transfection (Figure 5—figure supplements 7 and 8)

· Schematic diagram summarizing the effect of Mx2 on NLS-GFP-lacZ nuclear import (Figure 12B)

· Addition of the nuclear pore diagram to several relevant figures, to improve readability highlighting nucleoporins of interest where necessary (Figure 2B, Figure 3B, Figure 5, Figure 7C, and Figure 8C).

Essential revisions:

The reviewers recognize that this is already a very complex study but feel that some additional clarifications would strengthen the overall conclusions. These requests are divided into the following main categories:

1) Improve the readability of the manuscript:

- There are numerous western panels, which authors unfortunately present out of context, at many times just slices of immunoblots. This reporting style is unacceptable at many journals. Minimally, positions of migration standards should be shown alongside all panels.

We have added migration standards to the blots wherever it was feasible to do so without over cluttering the figures. Specifically, migration standards are now shown in Figures 1B, Figure 2A and Figure 2—figure supplement 2, Figure 8—figure supplements 2B and C. Since the same antibodies were used for Figures 3 and 5, migration standards were not included for these figures but we have marked the bands shown in smaller gel slices in Figure 3A, Figure 5—figure supplements 3-7.

- Because of the sheer amount of data presented in the figures, the readers will benefit from some schematic guide. The presentation style in Figure 4, specifically the diagram of the nuclear pore, is greatly appreciated. A smaller version of this nuclear pore in each relevant figure would help (like was done with Figure 6—figure supplement 1). More substantial figure legends and perhaps summary tables may also help orient readers and parse all of the information.

We have included the nuclear pore diagram in several figures (Figure 2B, Figure 3B, Figure 5, Figure 7C, and Figure 8C). In Figures 3 and 7, the diagrams highlight the nucleoporins investigated for CA binding or co-localization, respectively. We have also included a schematic summarizing the effect of Mx2 on nuclear import of NLS-GFP-lacZ fusion proteins (Figure 12B). We have also gone through the figure legends and tried to edit/extend for clarity where possible and appropriate.

2) Address the following considerations regarding the CA binding/pelleting assay used:

- This is not a novel assay but rather a standard Capsid binding assay conducted in physiologically irrelevant high salt (2M) concentration. Please re-phrase.

We apologise for the confusion regarding this point. This assay is distinct from those that have previously been reported, as it uses tubes assembled using only the CA protein, rather than CA-NC and ssDNA. The formation of CA-NC tubes has a propensity to pull-down non-specific charge-charge based interactions due to the presence of the highly charged NC and ssDNA in the assay. We have modified the text to clarify this point (subsection “Multiple Nups bind HIV-1 CA tubes in vitro.”).

- The high-salt condition (2M NaCl) may actually have unforeseen consequences on which proteins are seen binding to CA. It could be considered a very stringent assay in terms of electrostatic interactions. In fact, it is somewhat surprising that MxB co-pelleted under this condition, as the interaction likely has a strong electrostatic nature (it is known that the charged RRR at MxB N-terminus is important for binding). This may hint the MxB-capsid interaction is greatly enhanced either by avidity effect (oligomerization) or by very high local concentration when fixed at the NPC.

We do agree that 2M NaCl required for in vitro formation of CA tubes is represents a deviation from physiological conditions, but this is the only approach that allows the formation of biologically relevant CA tubes, as evidenced by cryo-EM structure of CA in complex with CypA in the presence of 2.25M NaCl (Liu et al., 2016). Furthermore, our assays have reliably revealed biologically relevant interactions (see below). The multiple washes performed after the pull-down assays allow us to remove unintended contaminants (see Figure 3—figure supplement 1).

2M NaCl can stabilize specific hydrophobic protein-protein contacts, but to our knowledge, there is no evidence that this NaCl concentration can trigger non-specific protein-protein interactions. Normally, NaCl in excess of 5M is required to observe unintended assemblies of proteins. It is true that 2M NaCl will interfere with protein-nucleic acid or protein-protein interactions that are primarily driven by charge-charge interactions. However, our experimental conditions are suitable for monitoring of protein-protein binding mediated by both ionic and hydrophobic interactions (such as CA interactions with Nups and Mx2). We have also included an additional figure (Figure 3—figure supplement 1B) which demonstrates CA interactions with CPSF6 are ablated by a single amino acid substitution (N74D, a single charge change).

- However, very high salt could enhance artifactual hydrophobic protein interactions, especially considering the inherent hydrophobicity of FG repeat containing proteins. Others who previously assessed binding of NUP153, e.g., to CA-based nanotubes performed assays under physiologically relevant salt (e.g., PBS), and the binding results here in 2M salt with different Nups do not always correlate with HIV-1 infection profile. Have authors tested binding in PBS? If so, how do these results compare to binding at high salt?

- Minimally, the potential for high salt to enhance binding artifacts should be discussed.

Again, see (Figure 3—figure supplement 1B) regarding the specificity of interactions, and not that the CPSF6-CA interaction involves a FG dipeptide. Additionally, as we noted in the text (subsection “Multiple Nups bind HIV-1 CA tubes *in vitro*.”), due to the multiple complex interactions between Nups, and the dramatic pleiotropic effects of Nup depletion, one would not necessarily expect CA binding to directly correlated with the effect of a specific Nup depletion. Furthermore, Nup88 does not contain FG repeats, indicating that hydrophobic FG-CA interactions are likely insufficient to explain our observations.

It is well established that CA tubes dissociate when NaCl concentrations are lowered to physiological levels, such as those in PBS. We have made similar observations, and this precluded us from conduction pull-down assays under these conditions.

The reasons for selecting high salt conditions are discussed in the revised manuscript.

3) Include cell viability controls under different knockdown conditions (optimally, growth rate):

- One expects that depletion of some (several?) of the targeted factors to grossly effect cell physiology, which could be assessed by cell growth rate, viability, cell cycle status, etc. Although authors do a fair amount of controls, including impressive cross-monitoring of all NUPs upon single NUP depletion, they provide basically no data assessing cell fitness outside of virus infection. In subsection “KPNB1” they state KPNB1 knockdown induced growth arrest, so it sounds like they have some of the relevant data. I realize at 35 (many multi-panel) figures we may already be looking at a record, but I nevertheless think it is critical to document impacts on cell physiology caused by the knockdowns.

In the revised manuscript, we have included cell-cycle profiles of for HeLa and HOS cells upon Nup depletion (Figure 5—figure supplements 7 and 8), which indicate some effect on cell cycle status, as we had previously indicated. Unfortunately, we were not able produce interpretable data from HT1080 in this assay. Additionally, the western blots showing LaminB1 and tubulin following Nup or NTR depletion serve as a proxy for viability of cells at the time of viral infection. Perhaps the most sensitive assay for perturbation of cell physiology is sensitivity of retroviral infection as we note, in the manuscript, FIV and EIAV infection are not dramatically affected by Nup knockdowns. Since each Nup or NTR had distinctive effects on infectivity depending on the virus or CA sequence, and on the nuclear import of Mx2(N25)-GFP-lacZ or SV40(NLS)-GFP-lacZ, and does not affect CPSF6 localization this indicates that the consequences of individual knockdowns were more specific than an aggregate effect on cell physiology.

4) Provide additional information regarding Mx2 experiments:

- Please show Mx2 induction in the different engineered cell lines by western blot.

This data is now included (Figure 1—figure supplement 1).

- Also clarify the statement regarding MxB "enhancement" of HIV infectivity, since MxB expression is returning infectivity to the wild-type level. It only modestly rescues gross infectivity defects in certain CA mutational backgrounds, which is different from promoting infection.

In certain instances, the effect of Mx2 on the infectivity in some instances is well beyond modest, as in the case of N57S infection in CsA treated HT1080 cells. We do however, take the reviewers point and have changed ‘enhancement’ to ‘increase’ or ‘rescue’ in several instances. At no point do we describe these phenotypes as ‘promoting’ infection.

5) Provide some evidence for whether or not primary human cells resemble any of the cell types tested in terms of Nups composition:

- It would have been useful to validate, at least, some of the results in HIV-1-infected primary cells to increase the relevance of the findings. Detailed protein expression profiles of primary human T cells or macrophages from healthy donors would add significance to the detailed NUP profiles reported for the cell lines.

In the revised manuscript, we have included expression Western blot analyses of Nup expression in primary human CD4^+^ T cells and macrophages from two healthy donors in Figure 2—figure supplements 2 and 3. This data reinforces our claim that Nup expression is heterogeneous among various cell types. This data is discussed in subsection “Variation in Nup expression among cell lines”.

6) Strengthen the statistic components of the manuscript:

- The number of experimental replicates across the board must be clarified. Although authors tabulate number of experimental repeats and variance (error bars) for infection experiments, one shortcoming is that they do not analyze samples cross wise to reveal which of the documented changes are significant (generally assessed as P < 0.05). Though generally important and required for publication, statistics really apply here as authors highlight differential effects of certain NUP depletions on virus infection in different cell types. As but one example in the Results, they highlight comparatively marginal role for RANBP2 in HT1080 and HOS cells as compared to HeLa cells. Are the requirements for RANBP2 in HT1018 and HOS significant or not? This is but one example; paper would be best served if all results are analyzed for significance, and resulting p values reported.

- p value analyses must be included for the innumerable comparisons that they make in text. Minimally, all text callouts to differences between samples, large or small, should be accompanied with stated p value.

- The Authors indicate in legends the number of repeats for the infection experiments, though generally fail to note numbers of times results were seen for other experimental approaches. Please also add this info to legends. In the manuscript heatmaps are used to summarize western blot results, which presumably analyzed several independent westerns. In such cases, indicate number of replicates behind the heatmaps, and that (one assumes) the associated blotting panels show a representative replicate.

We have included an additional Supplementary file (Supplementary file 1) which includes the following statistical analyses for all infectivity results:

1) P-values for the infectivity data shown in Figure 1 (Tab: Figure 1 Statistics)

– Effect of Mx2 in dividing and non-dividing cells

– Comparison of dividing and non-dividing cells

– Effect of CsA in dividing and non-dividing cells

– Effect of CsA on Mx2 activity in dividing and non-dividing cells

2) Analysis of significance of effects observed upon Nup/NTR depletion (Tab: Nup NTR Knockdown Statistics 1)

– Effect of siRNA knockdown on infectivity of HIV-1, other lentiviruses, and HIV-1 CA mutants

– Effect of siRNA knockdown on Mx2 activity against HIV-1, other lentiviruses, and HIV-1 CA mutants

– Effect of siRNA knockdown on the effect of CsA on the infection of WT HIV-1, HIV-1_A92E_, and HIV-1_N57S_.

– Effect of siRNA knockdown on Mx2 activity in the presence of CsA

– Effect of siRNA knockdown on Mx2(N91)-Arfaptin2 activity against HIV-1

3) Comparison of effects observed upon Nup/NTR depletion (Tab: Nup NTR Knockdown Statistics 2)

– Comparison of the effect of siRNA depletion on HIV-1, other lentiviruses, and HIV-1 CA mutants in HeLa, HT1080, and HOS cells

– Comparison of the effect of siRNA depletion on WT HIV-1 infection versus other lentiviruses, and HIV-1 CA mutants

– Comparison of the effect of siRNA depletion on Mx2 activity on WT HIV-1 infection versus other lentiviruses, and HIV-1 CA mutants

– Comparison of the effect of siRNA depletion on CsA activity on WT HIV-1 and HIV-1_A92E_ infection

– Comparison on the effect of siRNA depletion on the antiviral activity of Mx2 vs Mx2(N91)-Arfaptin2

Since each figure includes error bars and stated replicates, the effect sizes do not always correlate with the determined p-value, and the addition of p-values would substantially decrease the readability of the manuscript, we hope that this comprehensive file of statistics satisfies this criticism. The phenotypes highlighted for discussion in the text were chosen for effect size, novelty and/or interest based on what has already been reported to play a role in HIV-1 infection. Obviously we could not discuss every single data point in this paper, but any interested reader is free to examine the comprehensive data and statistics now provided.

Regarding the heat maps, they are intended as an alternative visual representation of the numerous blots shown in the accompanying figures, and represent only the blots shown. The figure legends have been modified to clarify this point and indicate how many replicates of blots were conducted for each experiment. We have ensured that each legend for all types of experimentation now indicates how many experiments were conducted and the number of replicates and that the data presented are representative.
